# Supplementary material for: Rapid escape of new SARS-CoV-2 Omicron variants from BA.2-directed antibody responses
Source: Cell Rep. 2023 Mar 7;42(4):112271. doi: 10.1016/j.celrep.2023.112271 (PMC9988707; doi:10.1016/j.celrep.2023.112271)
Supplement: Document S1. Figures S1–S4 and Tables S1–S4 — and supplemental references [file mmc1.pdf]

Cell Reports, Volume *42*

Supplemental information

Rapid escape of new SARS-CoV-2 Omicron variants from BA.2-directed antibody responses

Aiste Dijokaite-Guraliuc, Raksha Das, Daming Zhou, Helen M. Ginn, Chang Liu, Helen M.E. Duyvesteyn, Jiandong Huo, Rungtiwa Nutalai, Piyada Supasa, Muneeswaran Selvaraj, Thushan I. de Silva, Megan Plowright, Thomas A.H. Newman, Hailey Hornsby, Alexander J. Mentzer, Donal Skelly, Thomas G. Ritter, Nigel Temperton, Paul Klenerman, Eleanor Barnes, Susanna J. Dunachie, OPTIC consortium, Cornelius Roemer, Thomas P. Peacock, Neil G. Paterson, Mark A. Williams, David R. Hall, Elizabeth E. Fry, Juthathip Mongkolsapaya, Jingshan Ren, David I. Stuart, and Gavin R. Screaton

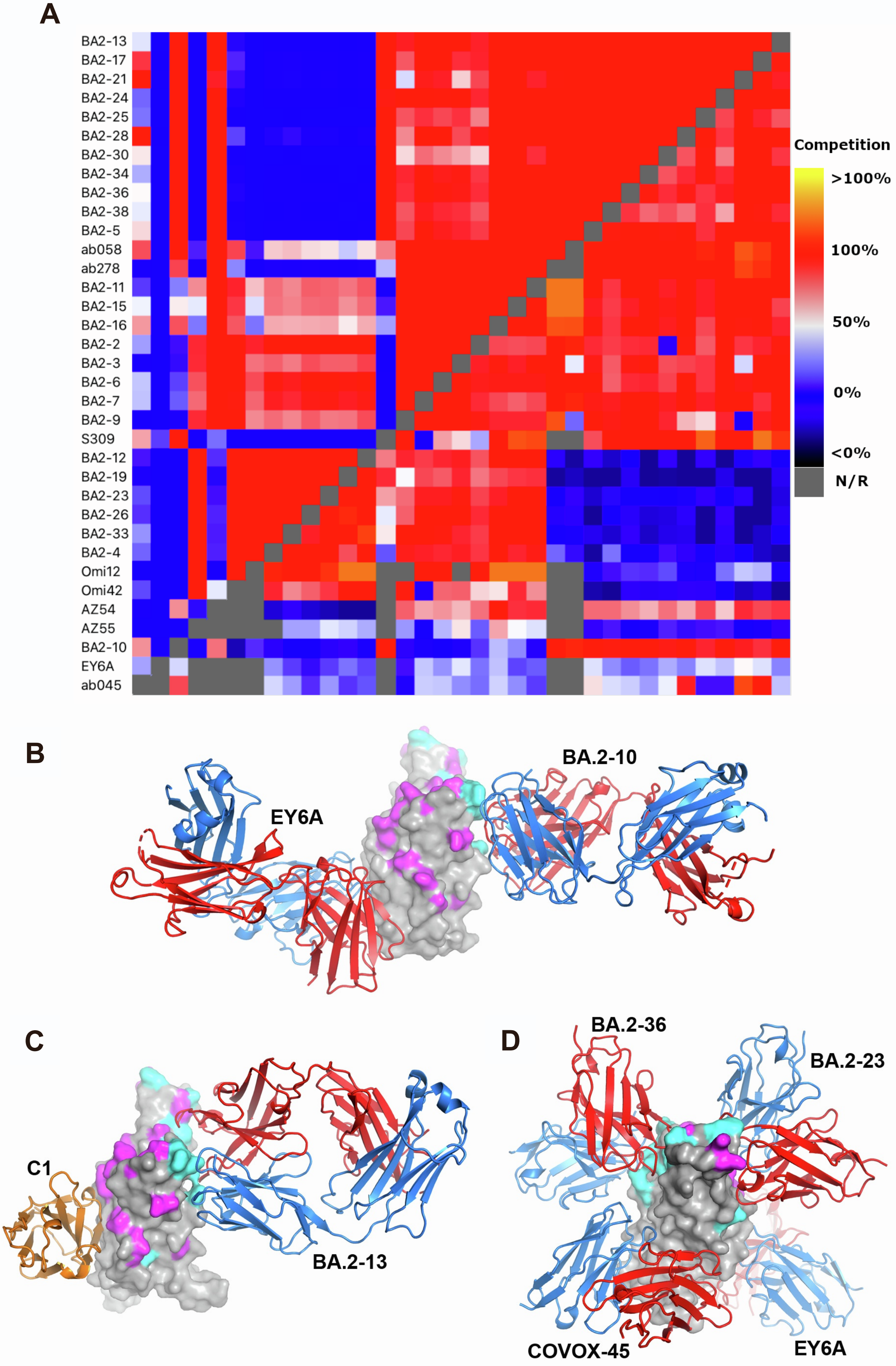

Figure S1. Mapping of all antibodies and ternary and quintuple complex structures. (A) Competition mapping, as measured by BLI (bottom right) and as calculated by *Mabscape* (top left). (B) Delta-RBD with BA.2-10 and EY6A Fabs. (C) Delta-RBD with BA.2-13 Fab and C1 nanobody. (D) Delta-RBD with BA.2-23, BA.2-36, EY6A and COVOX-45 Fabs. RBD is drawn as grey surface representation with BA.4 mutation sites highlighted in magenta and the additional mutation sites of all variants shown in Figure 1A are shown in cyan. Fabs are shown as ribbons with HC in red and LC in blue. The C1 nanobody in (B) is coloured in orange. Related to Figures 4,5 & 6.

Figure S1

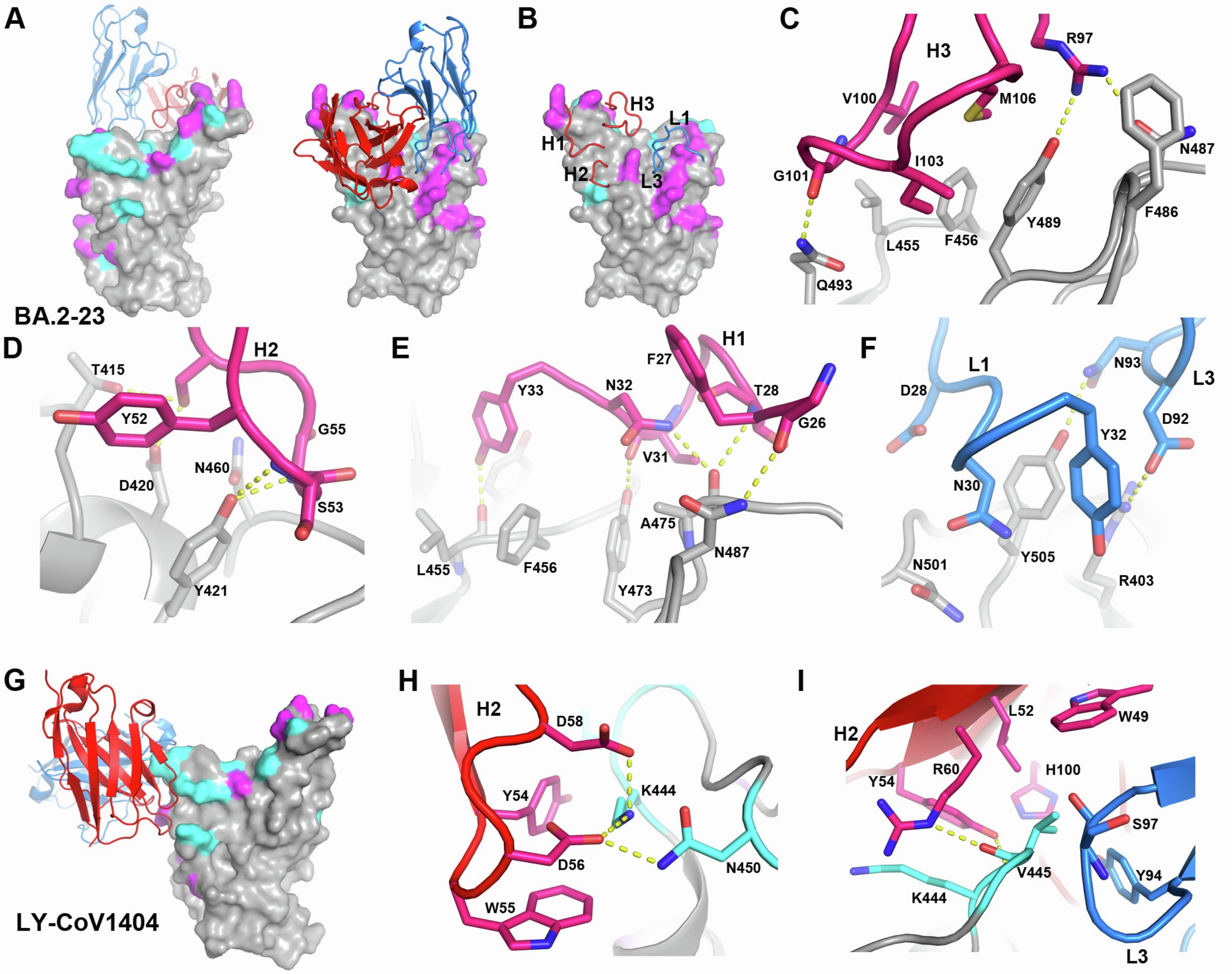

Figure S2. Structure of Delta-RBD and BA.2-23 complex and sensitivity of LY-CoV1404 to Omicron subvariants containing K444T or V450P mutation indicated by the structure of its complex with RBD. (A) overall structure of RBD and BA.2-23 complex. (B)-(F) details of interactions between RBD and BA.2-23. Drawing style and colour scheme are as in Figure 6. (G) Overall structure of RBD/LY-CoV1404 (PDB ID, 7MMO) as viewed from front of the RBD. The drawing style and colour scheme are as in (A). (H) Interactions of K444 of the RBD with CDR-H2 of LY-CoV1404. (I) Contacts between V445 of the RBD and LY-CoV1404. The side chains of the RBD, Fab HC and LC are shown as cyan, red and blue sticks respectively. Hydrogen bonds and salt bridges are shown as yellow broken bonds. Related to Figure 6.

Figure S2

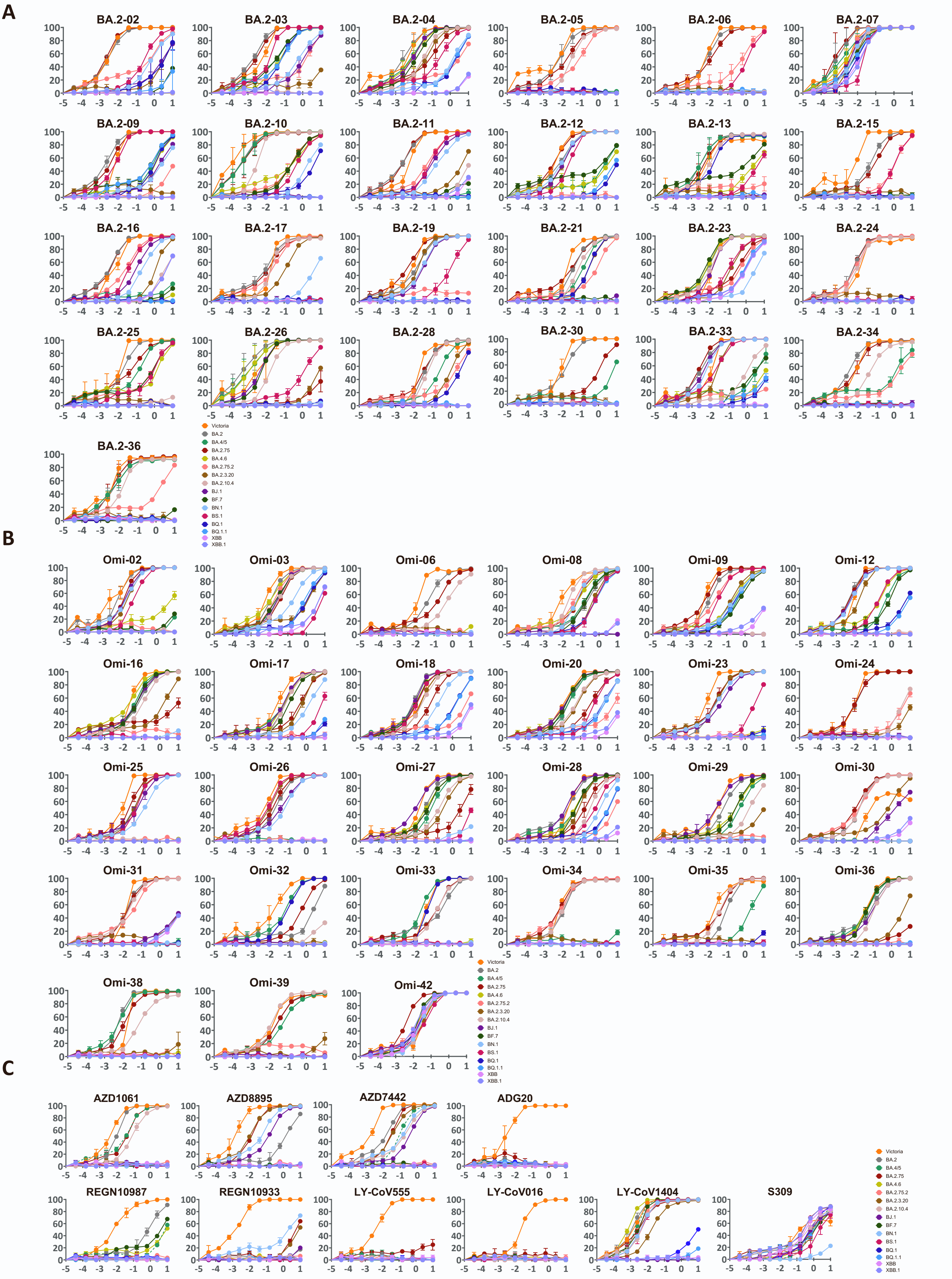

Figure S3. Neutralization assays. Neutralization curves using lentivirus pseudotyped with the S gene of the indicated BA.2 sub-lineages (A) BA.2 mAbs, (B) Omi-mAbs, (C) Commercial mAb. Related to Figure 3. Data for Omi-mAbs and Commercial mAbs against Victoria, BA.2, BA.2.75, BA.4/5, and BA.4.6 previously reported are included for comparison[S4,S5,S6].

Figure S3

Compiled IC50 titres of BA.1 mAb, data for various viruses

mAbs	WT virus								Psudo-virus																
	Victoria[13]	Alpha[13]	Beta[13]	Gamma[13]	Delta[13]	BA.1[13]	BA.1.1[13]	BA.2[13]	Victoria[24]	BA.2[24]	BA.2.12.1[23]	BA.4/5[24]	BA.4.6[41]	BA.2.75[42]	BA.2.75.2	BA.2.3.20	BJ.1	BF.7	BQ.1	BQ.1.1	BS.1	BA.2.10.4	BN.1	XBB	XBB.1
Omi-29 (3-53)	0.044	0.066	0.048	0.040	0.029	0.036	0.052	0.192	0.014	0.056	0.011	0.396	0.170	10.000	10.000	10.000	0.025	0.266	0.266	10.000	10.000	1.738	10.000	10.000	10.000
Omi-36 (3-66)	0.056	0.047	0.018	0.015	0.026	0.038	0.053	0.105	0.022	0.030	0.051	0.024	0.029	10.000	10.000	10.000	0.045	0.043	10.000	10.000	10.000	0.110	10.000	10.000	10.000
Omi-16 (3-66)	0.016	0.022	0.018	0.022	0.016	0.019	0.027	0.067	0.014	0.034	0.017	0.029	0.007	10.000	10.000	1.075	0.025	0.034	0.034	10.000	10.000	0.073	10.000	10.000	10.000
Omi-27 (3-66)	0.026	0.032	0.019	0.017	0.010	0.091	0.239	0.039	0.008	0.034	0.005	0.069	0.023	6.672	10.000	0.215	0.007	0.032	0.032	10.000	10.000	0.140	10.000	10.000	10.000
Omi-17 (3-66)	0.066	0.098	0.021	0.021	0.074	0.028	0.026	0.095	0.023	0.060	0.022	0.028	0.039	0.255	10.000	0.347	0.030	0.100	0.100	10.000	6.675	0.068	1.294	10.000	10.000
Omi-20 (3-66)	0.012	0.023	0.019	0.019	0.008	0.043	0.032	0.022	0.009	0.015	0.007	0.014	0.008	0.178	10.000	0.030	0.009	0.014	1.222	1.479	0.481	0.041	1.017	10.000	10.000
Omi-28 (3-66)	0.028	0.028	0.019	0.033	0.018	0.032	0.075	0.047	0.022	0.008	0.007	0.028	0.035	0.133	10.000	0.053	0.010	0.056	3.382	10.000	0.555	0.323	1.191	10.000	10.000
Omi-18 (3-53)	0.041	0.038	0.018	0.016	0.025	0.006	0.006	0.007	0.008	0.005	0.002	0.005	0.006	0.035	10.000	0.011	0.005	0.007	1.021	1.143	0.009	0.017	0.134	10.000	10.000
Omi-03 (3-53)	0.007	0.012	0.009	0.004	0.004	0.009	0.015	0.028	0.003	0.008	0.005	0.017	0.006	0.017	0.546	0.020	0.014	0.016	0.681	0.541	6.895	0.024	0.088	4.239	4.876
Omi-24 (1-69)	0.005	0.008	0.006	0.010	10.000	0.007	0.035	0.008	0.005	0.007	10.000	10.000	10.000	0.008	10.000	10.000	10.000	10.000	10.000	10.000	10.000	3.889	10.000	10.000	10.000
Omi-31 (1-69)	0.007	0.020	0.011	0.017	10.000	0.010	0.017	0.083	0.376	0.013	10.000	10.000	10.000	0.014	0.012	10.000	10.000	10.000	10.000	10.000	10.000	0.014	10.000	10.000	10.000
Omi-34 (1-69)	0.003	0.041	0.003	0.008	10.000	0.028	0.074	0.014	0.007	0.009	10.000	10.000	10.000	0.005	0.005	10.000	10.000	10.000	10.000	10.000	10.000	0.011	10.000	10.000	10.000
Omi-30 (1-69)	0.109	0.043	0.028	0.038	10.000	0.058	0.084	0.045	0.012	0.011	10.000	10.000	10.000	0.008	0.009	0.343	1.827	10.000	10.000	10.000	10.000	0.017	10.000	10.000	10.000
Omi-02 (1-69)	0.015	0.014	0.009	0.004	0.014	0.013	0.015	0.040	0.002	0.003	0.004	10.000	10.000	0.009	10.000	0.013	0.011	10.000	10.000	10.000	0.052	10.000	0.013	10.000	10.000
Omi-38 (1-69)	0.001	0.009	0.004	0.002	0.004	0.054	10.000	0.027	0.015	0.005	0.004	0.005	10.000	0.011	10.000	10.000	10.000	10.000	10.000	10.000	10.000	0.101	10.000	10.000	10.000
Omi-35 (3-9)	0.057	0.080	0.128	0.136	0.280	0.069	0.262	0.082	0.018	0.094	0.667	1.687	10.000	0.020	10.000	10.000	10.000	10.000	10.000	10.000	10.000	0.039	10.000	10.000	10.000
Omi-25 (3-9)	0.003	0.007	0.059	0.257	0.006	0.046	0.138	0.056	0.005	0.024	0.007	10.000	10.000	0.014	10.000	0.025	0.041	10.000	10.000	10.000	0.040	10.000	0.097	10.000	10.000
Omi-42 (3-9)	0.016	0.024	0.011	0.013	0.019	0.014	0.017	0.031	0.013	0.021	0.009	0.013	0.010	0.003	0.011	0.018	0.010	0.011	0.013	0.021	0.023	0.023	0.024	0.017	0.009
Omi-26 (1-18)	0.005	0.010	0.055	0.214	0.005	0.034	0.055	0.030	0.002	0.013	0.007	10.000	10.000	0.010	10.000	0.006	0.031	10.000	10.000	10.000	0.005	10.000	0.041	10.000	10.000
Omi-32 (3-33)	0.032	0.102	0.460	0.430	0.012	0.024	4.642	1.899	0.010	2.682	0.070	0.035	10.000	0.354	10.000	10.000	10.000	10.000	0.061	10.000	10.000	10.000	10.000	10.000	10.000
Omi-33 (3-33)	0.028	0.057	0.136	0.132	0.011	0.026	0.113	0.681	0.027	0.068	0.008	0.013	10.000	0.053	10.000	10.000	10.000	10.000	0.041	10.000	10.000	0.109	10.000	10.000	10.000
Omi-06 (4-4)	0.007	0.011	0.012	0.010	5.040	0.054	1.505	0.238	0.007	0.039	10.000	10.000	10.000	0.063	10.000	10.000	10.000	10.000	10.000	10.000	10.000	0.420	10.000	10.000	10.000
Omi-23 (4-31)	0.005	0.009	0.020	0.018	0.006	0.044	0.030	0.028	0.005	0.019	0.009	10.000	10.000	0.011	10.000	0.009	0.024	10.000	10.000	10.000	3.113	10.000	0.018	10.000	10.000
Omi-39 (3-43)	0.015	0.039	0.009	0.014	0.012	0.025	10.000	0.073	0.014	0.026	0.018	0.035	10.000	0.027	10.000	10.000	10.000	10.000	10.000	10.000	10.000	0.011	10.000	10.000	10.000
Omi-09 (3-30)	0.004	0.002	1.218	2.373	0.008	0.011	0.017	0.034	0.006	0.008	0.016	0.166	0.108	0.003	0.012	0.133	10.000	0.376	0.215	0.217	0.029	10.000	0.165	10.000	10.000
Omi-08 (1-46)	0.014	0.022	0.007	0.024	0.048	0.008	0.007	1.510	0.008	0.114	0.099	0.086	0.033	0.036	0.027	0.426	10.000	0.192	0.501	0.633	0.584	0.024	0.063	10.000	10.000
Omi-12 (1-58)	0.005	0.003	0.006	0.003	0.003	0.004	0.009	0.010	0.006	0.003	0.002	0.429	0.074	0.003	10.000	0.008	0.004	0.496	5.902	7.949	0.167	10.000	0.004	10.000	10.000

μg/mL
10.000
0.100
0.001

Figure S4 Heat map of IC50 neutralization titres for the panel of BA.1 (Omi) mAb. Pseudoviral neutralization IC50 titres for indicated mAb against a panel of pseudoviruses expressing variant S sequences. Live virus IC50 values against variants found earlier in the pandemic are included for comparison. Data for live virus assays[S3] and pseudoviral data for Victoria, BA.2, BA.2.12.1, BA.2.75, BA.4/5, and BA.4.6 previously reported are included for comparison[S3,S4,S5,S6,S7]. Related to Figure 3.

Figure S4

Table S1. Sources of BA.2 sub-lineage sequences, related to Figure 1.

Lineage	Defining RBD mutations	Example early genome	Submitting scientist, laboratory	Country/Region of earliest sequences	Date of earliest sequences	Pango issue, contributor
BA.4.6	BA.4/5 + R346T	EPI_ISL_12475182	Oliver et al, HOSPITAL UNIVERSITARIO SON ESPASES	Europe/South Africa	April 2022	#741, ryhisner
BA.4.7	BA.4/5 + R346S	EPI_ISL_12644817	Iranzadeh et al. NHLS/UCT	South Africa/Israel	April 2022	#777, FedeGueli
BF.7(BA.5.2.1.7)	BA.4/5 R346T	EPI_ISL_12810243	Coppens et al., Labo Klinische Biologie, UZA	Belgium	May 2022	#827, ryhisner
BQ.1(BA.5.3.1.1.1.1.1)	BA.4/5 K444T, N460K	EPI_ISL_14294806	Howard et al., Centers for Disease Control and Prevention Division of Viral Diseases, Pathogen Discovery	Nigeria	July 2022	#993, FedeGueli
BQ.1.1(BA.5.3.1.1.1.1.1.1)	BQ.1 + R346T	EPI_ISL_14752457	Christensen et al., Houston Methodist Hospital	USA	August 2022	#993, FedeGueli
BA.2.75	BA.2 + G339H, G446S, N460K, R493Q*	EPI_ISL_13302209	Khairnar et al. CSIR-NEERI, Nagpur Covid-19 Testing Lab	India	April 2022	#773, Silcn
BA.2.75.2	BA.2.75 + R346T, F486S	EPI_ISL_14290506	Gupta et al. ILBS/INSACOG	India	July 2022	#966, agamedilab
BN.1(BA.2.75.5.1)	BA.2.75 + R346T, K356T, F490S	EPI_ISL_14601644	Sima et al, Lifebrain Covid Labor GmbH	India	July 2022	#994, corneliusroemer
BJ.1(BA.2.10.1.1)	BA.2 + G339H, R346T, L368I, V445P, G446S, V483A, F490V	EPI_ISL_14166909	Maitra et al. National Institute of Biomedical Genomics – INSACOG	India	June 2022	#915, Silcn
BA.2.10.4	BA.2 + G446S, F486P, R493Q*, S494P	EPI_ISL_13929780	Karyakarte et al. Center for Genomics, Department of Microbiology, BJ Government Medical College and Sassoon Hospitals	India	June 2022	#898, Silcn
BS.1(BA.2.3.2.1)	BA.2 + R346T, L452R, N460K, G476S	EPI_ISL_14853710	Sekizuka et al. Pathogen Genomics Center, National Institute of Infectious Diseases	Japan ex Vietnam	August 2022	#1052, TakaKen6
BA.2.3.20	K444R, N450D, L452M, N460K, E484R, R493Q*	EPI_ISL_14723265	Selway et al, SA Pathology	USA/Singapore/Australia	August 2022	#1013, ryhisner
XBB	BA.2 + R346T, L368I, V445P, G446S, N460K, F486S, F490S, R493Q*	EPI_ISL_14917761	Ngan et al, National Public Health Laboratory, National Centre for Infectious Diseases	India	August 2022	#1058, corneliusroemer

Table S2. Variable gene usage BA.2 antibodies, related to Figure 1.

Ab id.	Protein-Specific	Heavy Chain					Light Chain				
		V-GENE and allele	J-GENE and allele	D-GENE and allele	V-REGION Nb of AA changes	CDR3 length	Light Chain	V-GENE and allele	J-GENE and allele	V-REGION Nb of AA changes	CDR3 length
BA.2-02	RBD	1-69*01 F, or 1-69D*01 F	5*02 F	3-22*01 F	10	19	κ	1-39*01 F, or 1D-39*01 F	4*01 F	7	9
BA.2-03	RBD	1-2*02 F	6*02 F	3-16*02 F	10	23	κ	3-11*01 F	5*01 F	6	9
BA.2-04	RBD	3-53*02 F	6*02 F	3-9*01 F	11	11	κ	1-9*01 F	5*01 F	8	10
BA.2-05	RBD	1-69*06 F, or 1-69*17 F	6*02 F	3-3*02 F	9	17	λ	1-47*01 F	3*02 F	10	11
BA.2-06	RBD	1-69*01 F, or 1-69D*01 F	4*02 F	3-22*01 F	8	20	κ	1-39*01 F, or 1D-39*01 F	1*01 F	4	9
BA.2-07	RBD	1-69*01 F, or 1-69D*01 F	5*02 F	3-22*01 F	13	19	κ	1-39*01 F, or 1D-39*01 F	4*01 F	8	9
BA.2-09	RBD	1-2*02 F	6*02 F	3-16*02 F	11	23	κ	3-11*01 F	5*01 F	8	9
BA.2-10	RBD	3-9*01 F	3*02 F	3-22*01 F	7	18	κ	1-39*01 F, or 1D-39*01 F	3*01 F	8	9
BA.2-11	RBD	1-69*01 F, or 1-69D*01 F	5*02 F	3-22*01 F	9	19	κ	1-39*01 F, or 1D-39*01 F	4*01 F	5	9
BA.2-12	RBD	3-9*01 F	6*02 F	2-21*02 F	8	16	κ	1-39*01 F, or 1D-39*01 F	2*01 F	13	9
BA.2-13	RBD	3-15*01 F	3*01 F	3-10*01 F	9	17	κ	1-39*01 F, or 1D-39*01 F	4*01 F	4	8
BA.2-15	RBD	1-69*01 F, or 1-69D*01 F	4*02 F	3-22*01 F	10	20	κ	1-39*01 F, or 1D-39*01 F	1*01 F	3	9
BA.2-16	RBD	1-69*01 F, or 1-69D*01 F	5*02 F	3-22*01 F	9	19	κ	1-39*01 F, or 1D-39*01 F	4*01 F	7	9
BA.2-17	RBD	1-69*06 F	6*02 F	3-3*01 F	9	19	λ	2-14*01 F	2*01 F, or 3*01 F	8	11
BA.2-19	RBD	3-48*03 F	4*02 F	1-26*01 F	8	13	κ	1-5*03 F	1*01 F	6	10
BA.2-21	RBD	3-9*01 F	4*02 F	5-24*01 ORF	11	16	λ	1-40*01 F	3*02 F	7	11
BA.2-23	RBD	3-53*04 F	6*02 F	1-26*01 F	12	13	κ	1-33*01 F, or 1D-33*01 F	5*01 F	8	9
BA.2-24	RBD	1-69*09 F	4*02 F	3-22*01 F	13	16	κ	1-39*01 F, or 1D-39*01 F	5*01 F	6	9
BA.2-25	RBD	4-59*01 F	4*02 F	5-12*01 F	16	16	κ	2-28*01 F, or 2D-28*01 F	3*01 F	7	9
BA.2-26	RBD	3-66*01 F, or 3-66*04 F	5*01 F, or 5*02 F	2-15*01 F	16	10	κ	1-33*01 F, or 1D-33*01 F	4*02 (F)	10	8
BA.2-28	RBD	3-9*01 F	6*02 F	6-6*01 F	14	19	λ	3-21*02 F	2*01 F, or 3*01 F	6	11
BA.2-30	RBD	4-59*03 F	4*02 F	5-12*01 F	13	16	κ	2-28*01 F, or 2D-28*01 F	3*01 F	6	9
BA.2-33	RBD	4-61*11 (F)	5*02 F	2-15*01 F	16	14	λ	1-47*01 F	2*01 F, or 3*01 F	13	11
BA.2-34	RBD	1-69*09 F	4*02 F	2-21*02 F	11	12	κ	1-5*01 F	1*01 F	4	8
BA.2-36	RBD	4-61*02 F, or 4-61*11 (F)	4*02 F	6-25*01 F	17	11	κ	1-5*01 F	1*01 F, or 4*02 (F)	7	8

Table S2

Table S3. Structural data collection, analysis and model statistics, related to Figure 6.

**Method**	X-ray Crystallography				Cryo-EM
**Structure**	Delta-RBD/BA.2-10/EY6A	Delta-RBD/BA.2-13/C1	Delta-RBD/BA.2-36		Delta-RBD/BA.2-23/BA.2-36/EY6A/Fab-45
**PDB/EMBD ID**	8BBN	8C3V	8BBO		8BCZ, EMD-15971
**Data collection**					
Space group	$P2_12_12_1$	$P2_12_12_1$	$P4_1$	Voltage (kV)	300
Cell dimensions				Frames (EER fractions)	50
a, *b*, *c* (Å)	170.7, 171.7, 177.0	105.9, 160.8, 172.4	99.2, 99.2, 86.1	Dose rate ($e^-/Å^2/s$)	11.5
α, β, γ (°)	90, 90, 90	90, 90, 90	90, 90, 90	Total dose ($e^-/Å^2$)	50
Resolution (Å)	56–3.58 (3.65–3.58)[a]	80–2.74 (2.79–2.74)	54–2.75 (2.80–2.75)	Calibrated pixel size ($Å^2$)	0.7303
R_{merge}	0.467 (---)	0.257	0.460 (---)	Defocus (µm)	0.8-2.6
R_{pim}	0.130 (1.771)	0.072 (1.078)	0.90 (2.066		
I/*s*(*I*)	4.1 (0.3)	7.4 (0.4)	7.5 (0.4)	Movies	20,535
$CC_{1/2}$	0.992 (0.301)	0.995 (0.317)	0.994 (0.390)	Particles (final)	167,492
Completeness (%)	100 (97.2)	100 (99.5)	100 (99.9)	Map resolution (Å)	2.9
Redundancy	13.8 (13.2)	13.7 (14.1)	26.8 (18.3)	Sharpening B-factor ($Å^2$)	90.5
**Refinement**					
Resolution (Å)	56–3.58	67–2.74	50–2.75	Resolution (Å)	2.9
No. reflections	52453/2840	73940/3933	20668/1102	No. protein atoms	8505
R_{work} / R_{free}	0.265/0.313	0.216/0.259	0.224/0.252	*B* factors ($Å^2$)	62
No. atoms				r.m.s. deviations	
Protein	24354	17512	4738	Bond lengths (Å)	0.003
Ligand/ion/water		285	14	Bond angles (°)	0.5
B factors ($Å^2$)				Clash score	6.2
Protein	174	93	81	Ramachandran outlier (%)	0
Ligand/ion/water		101	126	Rotamer outlier (%)	1.2
r.m.s. deviations				d FSC model (0.5)	3.1
Bond lengths (Å)	0.002	0.003	0.002	CC (mask)	0.83
Bond angles (°)	0.5	0.6	0.5		

Table S4. Sample information, related to Star Methods.

	BA.1 infection	BA.2 infection	BA.4/5 infection	BNT162b2 V3+28	
Participants					
Female	6	19	6	8	
Male	3	4	5	10	
Median Age (Y)	31 (Range 21-55)	41 (Range 22-57)	42 (Range 20-94)	45 (Range 30-59)	

Supplemental References

S1. Dejnirattisai, W., Zhou, D., Ginn, H.M., Duyvesteyn, H.M.E., Supasa, P., Case, J.B., Zhao, Y., Walter, T.S., Mentzer, A.J., Liu, C., et al. (2021). The antigenic anatomy of SARS-CoV-2 receptor binding domain. Cell *184*, 2183-2200.

S2. Liu, C., Zhou, D., Nutalai, R., Duyvestyn, H., Tuekprakhon, A., Ginn, H., Dejnirattisai, W., Supasa, P., Mentzer, A., Wang, B., et al. (2021). The Beta mAb response underscores the antigenic distance to other SARS-CoV-2 variants. Cell, Host and Microbe *30*, 53-68.

S3. Nutalai, R., Zhou, D., Tuekprakhon, A., Ginn, H., Supasa, P., Liu, C., Huo, J., Mentzer, A., Duyvesteyn, H.M.E., Dijokaite-Guraliuc, A., et al. (2022). Potent cross-reactive antibodies following Omicron breakthrough in vaccinees. Cell *185*, 2116-2131.

S4. Tuekprakhon, A., Nutalai, R., Dijokaite-Guraliuc, A., Zhou, D., Ginn, H.M., Selvaraj, M., Liu, C., Mentzer, A.J., Supasa, P., Duyvesteyn, H.M.E., et al. (2022). Antibody escape of SARS-CoV-2 Omicron BA.4 and BA.5 from vaccine and BA.1 serum. Cell 185, 2422-2433 e2413. 10.1016/j.cell.2022.06.005.

S5. Dijokaite-Guraliuc, A., Das, R., Nutalai, R., Zhou, D., Mentzer, A.J., Liu, C., Supasa, P., Dunachie, S.J., Lambe, T., Fry, E.E., et al. (2022). Antigenic characterization of SARSCoV-2 Omicron subvariant BA.4.6. Cell Discov 8, 127. 10.1038/s41421-022-00493-0.

S6. Huo, J., Dijokaite-Guraliuc, A., Liu, C., Zhou, D., Ginn, H.M., Das, R., Supasa, P., Selvaraj, M., Nutalai, R., Tuekprakhon, A., et al. (2023). A delicate balance between antibody evasion and ACE2 affinity for Omicron BA.2.75. Cell Rep 42, 111903. 10.1016/j.celrep.2022.111903.

S7. Huo, J., Dijokaite-Guraliuc, A., Nutalai, R., Das, R., Zhou, D., Mentzer, A., Fry, E., Mongkolsapaya, J., Ren, J., Stuart, D., and Screaton, G. (2022). Humoral responses against SARS-CoV-2 Omicron BA.2.11, BA.2.12.1 and BA.2.13 from vaccine and BA.1 serum. Cell Discovery.
