# Supplementary material for: Rapid escape of new SARS-CoV-2 Omicron variants from BA.2-directed antibody responses
Source: Cell Rep. 2023 Mar 7;42(4):112271. doi: 10.1016/j.celrep.2023.112271 (PMC9988707; doi:10.1016/j.celrep.2023.112271)
Supplement: Document S2. Article plus supplemental information [file mmc6.pdf]

Cell Reports

Article

Rapid escape of new SARS-CoV-2 Omicron variants from BA.2-directed antibody responses

Graphical abstract

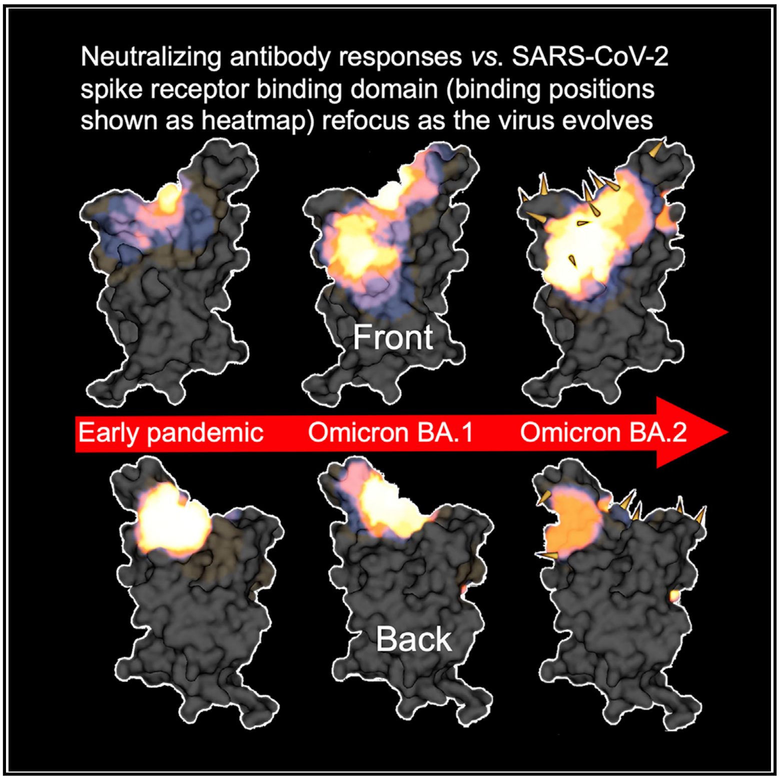

Highlights

- 25 potent RBD mAbs generated from vaccinees suffering BA.2 breakthrough infections
- Potent mAbs bind 3 clusters, 2 correspond to early-pandemic binding hotspots
- RBD mutations in recent variants map close to these binding sites
- Neutralization activity of all but 1 potent mAb is reduced

Authors

Aiste Dijokaite-Guraliuc, Raksha Das, Daming Zhou, ..., Jingshan Ren, David I. Stuart, Gavin R. Screaton

Correspondence

liz@strubi.ox.ac.uk (E.E.F.),
juthathip.mongkolsapaya@well.ox.ac.uk (J.M.),
ren@strubi.ox.ac.uk (J.R.),
dave@strubi.ox.ac.uk (D.I.S.),
gavin.screaton@medsci.ox.ac.uk (G.R.S.)

In brief

Dijokaite-Guraliuc et al. analyze potently neutralizing antibodies from vaccinated individuals with BA.2 breakthrough infections. The antibodies bind 3 sites on the receptor-binding domain, 2 of which are in common with early-pandemic antibodies. Mutations in more recent variants map closely to these sites, leading to reduced neutralization in all but one mAb.

Dijokaite-Guraliuc et al., 2023, Cell Reports *42*, 112271
April 25, 2023 © 2023 The Authors.
https://doi.org/10.1016/j.celrep.2023.112271

Article

Rapid escape of new SARS-CoV-2 Omicron variants from BA.2-directed antibody responses

Aiste Dijokaite-Guraliuc,[1,19] Raksha Das,[1,19] Daming Zhou,[2,3,19] Helen M. Ginn,[4,19] Chang Liu,[1,3,19] Helen M.E. Duyvesteyn,[2] Jiandong Huo,[2] Rungtiwa Nutalai,[1] Piyada Supasa,[1] Muneeswaran Selvaraj,[1] Thushan I. de Silva,[5,6] Megan Plowright,[5,6] Thomas A.H. Newman,[5,6] Hailey Hornsby,[5] Alexander J. Mentzer,[1,7] Donal Skelly,[7,8,9] Thomas G. Ritter,[7] Nigel Temperton,[10] Paul Klenerman,[7,8,11,12] Eleanor Barnes,[7,8,11,12] Susanna J. Dunachie,[7,8,13,14,15] OPTIC consortium, Cornelius Roemer,[16,17] Thomas P. Peacock,[18] Neil G. Paterson,[4] Mark A. Williams,[4] David R. Hall,[4] Elizabeth E. Fry,[2,*] Juthathip Mongkolsapaya,[1,3,*] Jingshan Ren,[2,*] David I. Stuart,[2,3,4,20,*] and Gavin R. Screaton[1,3,*]

[1]Wellcome Centre for Human Genetics, Nuffield Department of Medicine, University of Oxford, Oxford, UK
[2]Division of Structural Biology, Nuffield Department of Medicine, University of Oxford, The Wellcome Centre for Human Genetics, Oxford, UK
[3]Chinese Academy of Medical Science (CAMS) Oxford Institute (COI), University of Oxford, Oxford, UK
[4]Diamond Light Source, Ltd., Harwell Science & Innovation Campus, Didcot, UK
[5]Department of Infection, Immunity and Cardiovascular Disease, University of Sheffield, Sheffield, UK
[6]Sheffield Teaching Hospitals NHS Foundation Trust, Sheffield, UK
[7]Oxford University Hospitals NHS Foundation Trust, Oxford, UK
[8]Peter Medawar Building for Pathogen Research, Oxford, UK
[9]Nuffield Department of Clinical Neurosciences, University of Oxford, Oxford, UK
[10]Viral Pseudotype Unit, Medway School of Pharmacy, University of Kent and Greenwich Chatham Maritime, Kent, UK
[11]NIHR Oxford Biomedical Research Centre, Oxford, UK
[12]Translational Gastroenterology Unit, University of Oxford, Oxford, UK
[13]Centre for Tropical Medicine and Global Health, Nuffield Department of Medicine, University of Oxford, Oxford, UK
[14]Mahidol-Oxford Tropical Medicine Research Unit, Bangkok, Thailand
[15]Department of Medicine, University of Oxford, Oxford, UK
[16]Biozentrum, University of Basel, Basel, Switzerland
[17]Swiss Institute of Bioinformatics, Basel, Switzerland
[18]Department of Infectious Disease, Imperial College London, London, UK
[19]These authors contributed equally
[20]Lead contact
*Correspondence: liz@strubi.ox.ac.uk (E.E.F.), juthathip.mongkolsapaya@well.ox.ac.uk (J.M.), ren@strubi.ox.ac.uk (J.R.), dave@strubi.ox.ac.uk (D.I.S.), gavin.screaton@medsci.ox.ac.uk (G.R.S.)
https://doi.org/10.1016/j.celrep.2023.112271

SUMMARY

In November 2021, Omicron BA.1, containing a raft of new spike mutations, emerged and quickly spread globally. Intense selection pressure to escape the antibody response produced by vaccines or severe acute respiratory syndrome coronavirus 2 (SARS-CoV-2) infection then led to a rapid succession of Omicron sub-lineages with waves of BA.2 and then BA.4/5 infection. Recently, many variants have emerged such as BQ.1 and XBB, which carry up to 8 additional receptor-binding domain (RBD) amino acid substitutions compared with BA.2. We describe a panel of 25 potent monoclonal antibodies (mAbs) generated from vaccinees suffering BA.2 breakthrough infections. Epitope mapping shows potent mAb binding shifting to 3 clusters, 2 corresponding to early-pandemic binding hotspots. The RBD mutations in recent variants map close to these binding sites and knock out or severely knock down neutralization activity of all but 1 potent mAb. This recent mAb escape corresponds with large falls in neutralization titer of vaccine or BA.1, BA.2, or BA.4/5 immune serum.

INTRODUCTION

There have been billions of infections and millions of deaths since severe acute respiratory syndrome coronavirus 2 (SARS-CoV-2) emerged in Wuhan in late 2019. Since its zoonotic jump, SARS-CoV-2 has been under intense selective pressure to adapt to its new human host and evade the immune response.[1] The SARS-CoV-2 RNA polymerase is intrinsically error prone, and it has been estimated that every single-nucleotide change in the SARS-CoV-2 RNA genome will be generated in an infected individual every day.[2] Large-scale genomic sequencing efforts in a number of countries have charted progressive evolution of SARS-CoV-2 (https://www.cogconsortium.uk; https://www.cdc.gov/coronavirus/2019-ncov/variants/variant-classifications.html).

This is an open access article under the CC BY license (http://creativecommons.org/licenses/by/4.0/).

The spike protein (S) mediates cell entry by binding the angiotensin-converting enzyme 2 (ACE2) cellular receptor,[3] and many neutralizing antibodies bind S.[4–7] Mutations in S can therefore impart two important selective advantages: firstly, mutations to the ACE2-binding surface in the receptor-binding domain (RBD) can increase affinity for ACE2, potentially increasing viral transmissibility.[8–11] Secondly, mutations at the site of interaction of neutralizing antibody on S may reduce neutralizing titers, allowing reinfection or breakthrough of vaccines.[12] Structure function analyses of panels of human monoclonal antibodies (mAbs) generated from convalescent SARS-CoV-2 cases have been performed by a number of laboratories, giving insight into the mechanisms of viral neutralization.[4,6,7,13,14] Most potently neutralizing antibodies bind on, or near, the ACE2 interaction surface on the RBD to prevent interaction with ACE2. A second group, exemplified by mAb S309, binds close to the N-linked glycan at residue 343, does not block interaction with ACE2, and may destabilize the S trimer.[15] Note that bebtelovimab (Ly-CoV1404) competes with S309 yet blocks ACE2 binding.[16] A third site of binding of potent mAbs is to the N-terminal domain (NTD) in S1[5,17]; these mAbs do not antagonize ACE2 binding, and the mechanism of neutralization is not yet clear, although steric hindrance of ACE2 interaction at the cell surface has been proposed.[5] A total of five epitopes were defined for early-pandemic RBD-binding mAbs based on a torso analogy and were defined as left shoulder, right shoulder, neck, right flank, and left flank, with the latter two corresponding to less potently neutralizing antibodies.[4]

Early during the pandemic, when population immunity was low, S mutations such as N501Y in Alpha, which increases affinity for ACE2,[10] were likely driven by selection for increased transmissibility,[18] while those occurring on the background of strong herd immunity from vaccines and infection are likely selected for their ability to escape the neutralizing antibody response. SARS-CoV-2 executed a large sequence jump to Omicron BA.1, which carries 30 substitutions plus the deletions of 6 and an insertion of 3 residues compared with the ancestral Wuhan spike sequence, leading to a large antigenic distance from preceding strains.[13,19] Most mutations were clustered in the RBD and NTD and led to large falls or knockout of neutralizing titers of sera from vaccinees or following SARS-CoV-2 infection.[12] In addition, neutralizing activity of several therapeutic mAbs was reduced or knocked out against BA.1[12,20,21].

Omicron emerged as at least 3 distinct lineages in November 2021. BA.1 caused the first Omicron wave, closely followed by a combination of BA.1 sub-lineages BA.1.1 and BA.2 in early 2021 (https://cov-lineages.org/lineage_list, https://nextstrain.org/nextclade/sars-cov-2).[22] BA.2 became globally dominant and continues to spawn a succession of variants: first BA.2.12.1,[23] followed by BA.4 and BA.5,[24] with BA.5 being the most widespread strain globally in June 2022 (https://covspectrum.org/explore/United%20Kingdom/AllSamples/Past6M).

Following BA.5, several new trends were observed in the evolution of Omicron: (1) the emergence of "second-generation" BA.2 variants (including derivatives of BA.5)—variants with long phylogenetic branch lengths, multiple antigenic mutations, and a lack of genetic intermediates, for example BA.2.75, BJ.1, BS.1, BA.2.10.4, and BA.2.3.20,[19] and (2) antigenic drift, seen both in BA.5[24] and within these second-generation BA.2 lineages, notably BQ.1 and BA.2.75 (https://nextstrain.org/nextclade/sars-cov-2/21L) (Figures 1A–1C). Finally, recombination between two of these second-generation variants (BJ.1 and BM.1.1.1) has produced XBB (Figure 1C). Many of these variants show a large degree of convergent evolution in known antigenic RBD residues, and mutations lie in areas that may threaten the binding of neutralizing antibodies, leading to escape from protection afforded by vaccine or previous SARS-CoV-2 infection, including prior Omicron infection. These second-generation BA.2 variants are now dominant globally, with BQ.1 alone accounting for 50% of infections as of December 27, 2022 (https://cov-spectrum.org/explore/World/AllSamples/Past6M) and XBB.1.5 (XBB.1 + F486P currently expanding rapidly in North America; https://cov-spectrum.org/explore/North%20America/AllSamples/Past6M).

In this report, we generate a panel of 25 potent mAbs (IC50 < 100 ng/mL) from vaccinees suffering breakthrough Omicron BA.2 infections. We have determined structures for complexes of four of these antibodies by X-ray crystallography and cryoelectron microscopy (cryo-EM) analysis. To generalize the structural information to all 25 antibodies, we used biolayer interferometry (BLI) competition measurements and prior structures to create an interaction map of the antibody panel; potent mAbs bind to three distinct patches on the RBD, one of which is quite extended and reaches between two of the five epitopes seen in early-pandemic responses, while the other two correspond to diminished responses at hotspots for potent antibody binding in early-pandemic responses. This corresponds to a refocusing of the response, similar to that seen for BA.1.[13] The positions of mutations in the new BA.2 sub-lineages strongly overlap with the positions of antibody binding.[25] Pseudoviral neutralization assays confirmed knockout or severe reduction in neutralization titers for all but one of the antibody panels by one or more variants. There were also large falls in serum neutralization titers from vaccinees or from Omicron-infected individuals. Finally, all BA.2 mAbs showed potent neutralization of an early-pandemic SARS-CoV-2 strain and are likely derived from the memory B cell response to vaccination. Reliance on the recall response may over time narrow the polyclonality of neutralizing antibodies causing increased focusing on a smaller number of epitopes.

RESULTS

Emerging BA.2, BA.4, and BA.5 sub-lineages

At present, a number of lineages are growing rapidly from within both the BA.2 and BA.5 branches. Most striking is the large degree of convergent evolution, particularly at antigenic RBD positions such as 346, 444, 452, 460, 486, 490, 493, and 494. These lineages include examples from the BA.4/5 branches (which contain L452R, F486V, and the reversion R493Q), such as BA.4.6 and BF.7 (R346T); BA.4.7 (R346S); BQ.1 (K444T, N460K); and BQ.1.1 (R346T, K444T, N460K) and from the BA.2.75 branch (which contains G339H, G446S, N460K, and the reversion R493Q), such as BA.2.75.2 (R346T and F486S and BA.2.75 mutations); BN.1 (also known as [aka] BA.2.75.5.1 with R346T, K356T, F490S, and BA.2.75 mutations); and BM.1.1.1 (aka BA.2.75.3.1.1.1 with R346T, F486S, F490S, and

CellPress
OPEN ACCESS

A

Variant	Mutations in RBD area																
BA.2.12.1									L452Q							Q493R	
BA.4									L452R					F486V			
BA.4.6		R346T							L452R					F486V			
BA.4.7		R346S							L452R					F486V			
BA.2.75	G339H						G446S			N460K							
BA.2.75.2	G339H	R346T					G446S			N460K				F486S			
BA.2.3.20					K444R			N450D	L452M	N460K			E484R				
BJ.1	G339H	R346T		L368I		V445P	G446S					V483A			F490V	Q493R	
BA.2.10.4							G446S							F486P			S494P
BQ.1					K444T				L452R	N460K				F486V			
BQ.1.1		R346T			K444T				L452R	N460K				F486V			
BF.7		R346T							L452R					F486V			
BN.1	G339H	R346T	K356T				G446S			N460K					F490S		
BS.1		R346T							L452R	N460K	G476S					Q493R	
XBB		R346T		L368I		V445P	G446S			N460K				F486S	F490S	Q493R	
Frequency	4	9	1	2	3	2	6	1	8	8	1	1	1	8	3	4	1

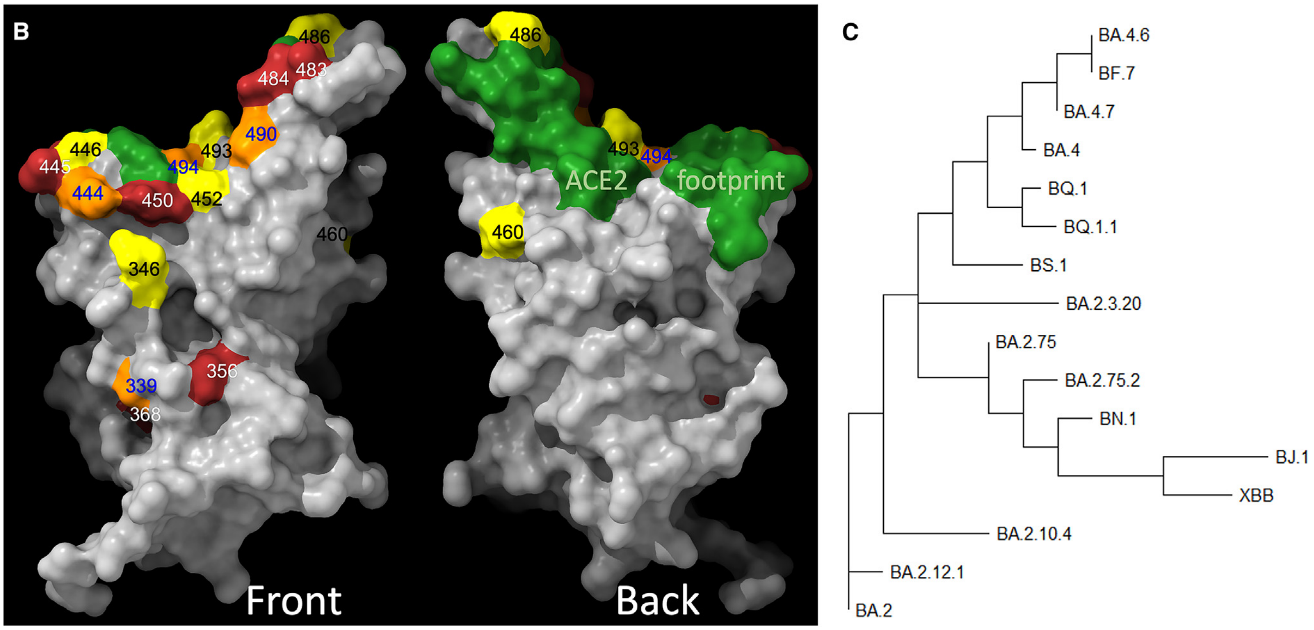

Figure 1. Phylogeny of BA.2 sub-variants
(A) Alignments of mutated RBD amino acid substitutions; these are mutations formed on the BA.2 background, i.e., they are in addition to mutations found in BA.2.
(B) Amino acid substitutions present in sub-variants positioned on the RBD surface. Coloring is according to the frequency count for the change in the various sub-variants, shown in (A). The ACE2 footprint is shown in green.
(C) Phylogenetic tree of a selection of BA.2/4/5 sub-lineages.
See also Tables S1 and S2.

BA.2.75 mutations). There are also examples of several other second-generation BA.2 variant lines such as BJ.1 (aka BA.2.10.1.1; G339H, R346T, L368I, V445P, G446S, V483A, and F490V); BA.2.10.4 (G446S, F486P, S494P, and the R493Q reversion); BS.1 (aka BA.2.3.2.1; R346T, L452R, N460K, G476S, and the Q493R reversion); BA.2.3.20 (K444R, N450D, L452M, N460K, E484R, and the Q493R reversion); and finally a BJ.1 × BM.1.1.1 (aka BA.2.75.3.1.1.1) recombinant, XBB (which, relative to BA.2, contains R346T, L368I, V445P, G446S, N460K, F486S, F490S, and the Q493R reversion). These sequence changes are compiled in Figure 1A and Table S1, and the distribution of the mutations on the surface of the RBD is shown in Figure 1B. The rooted phylogenetic tree[26] (https://nextstrain.org/nextclade/sars-cov-2) shown in Figure 1C provides a helpful grouping of the lineages.

Outside the RBD, the degree of convergent evolution is lesser but still present. Many of the second-generation BA.2 variant lineages contain deletions or mutations in the NTD, often similar to those seen in the variants of concern (VOC), for example Δ∼144 in BJ.1, BS.1, and BA.2.10.4 (previously seen in Alpha and BA.1) and NSP12 G671S in BJ.1, XBB, and BA.2.10.4 (previously seen in Delta).

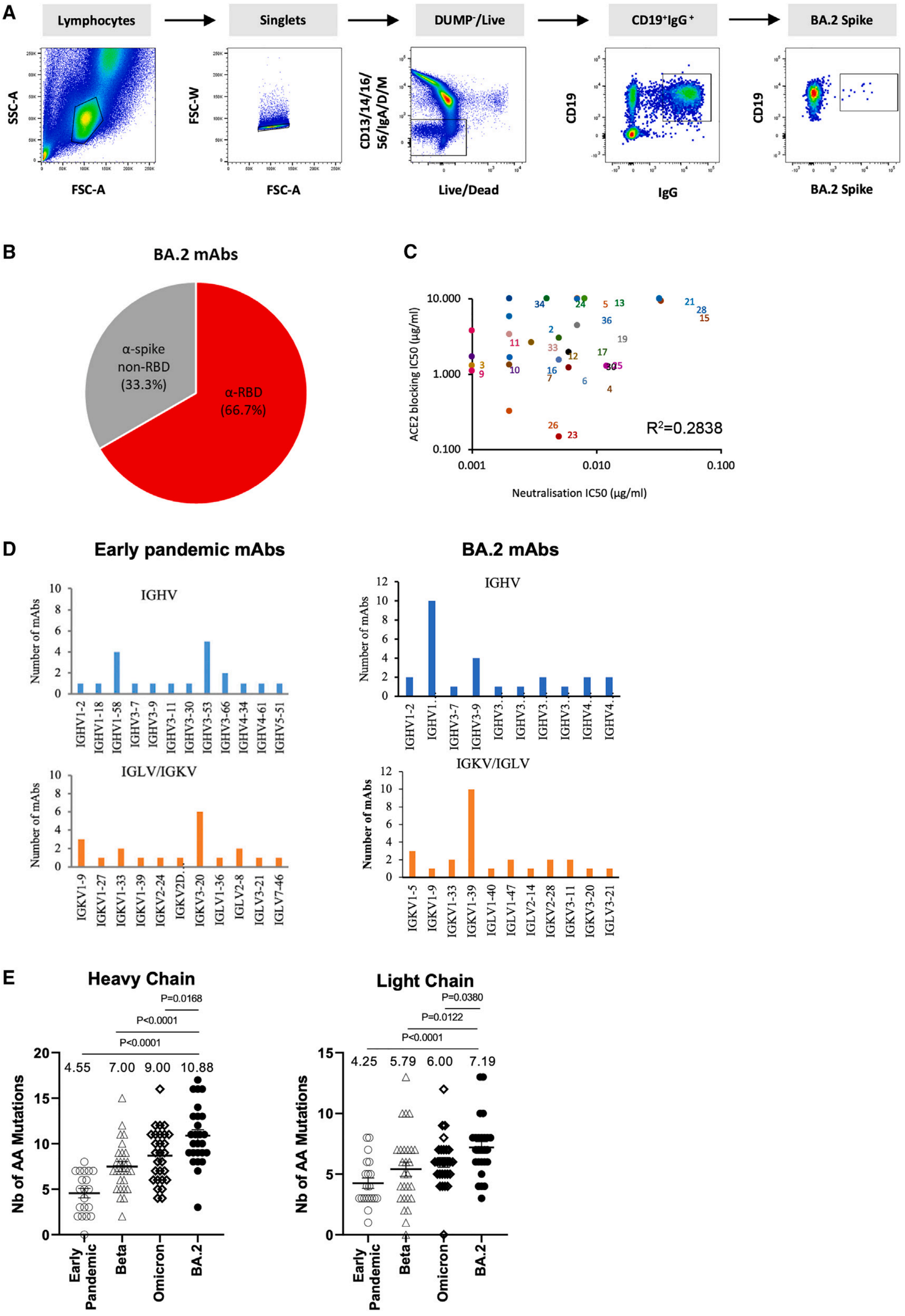

(legend on next page)

Generation of mAbs from vaccinees suffering BA.2 breakthrough infections

Blood was obtained from 7 volunteers, all triple vaccinated >24 days (median 29) from PCR- (n = 1) or lateral flow test-confirmed (n = 6) SARS-CoV-2 infection, during the BA.2 wave in April 2022 (https://cov-spectrum.org/explore/United%20Kingdom/AllSamples/from=2022-04-01&to=2022-04-30/variants?&). To select samples for antibody production, we first performed focus reduction neutralization assays (FRNTs) on BA.2 virus. Three samples with the highest titers were selected for mAb production, and single immunoglobulin G (IgG)-positive B cells were stained and sorted using BA.2 S trimer (Figure 2A). Following a degenerate PCR reaction, heavy and light chains (HC and LCs) were assembled into an expression vector using Gibson assembly, and the products were expressed by transient transfection. Supernatants were tested for reactivity to full-length BA.2 S or BA.2 RBD; in common with the panel of mAbs generated following Omicron BA.1 infection,[13] a high proportion (67%) of mAbs recognized the BA.2 RBD (Figure 2B). Supernatants were also tested in BA.2 neutralization assays, and those showing potent neutralization (FRNT50 < 100 ng/mL) were selected for further study. From 672 sorted cells, 383 antibodies were recovered, leading to the generation of 25 potent mAbs.

We tested the ability of mAbs to block ACE2 interaction with S (Figure 2C); a number of mAbs (BA.2–5, 13, 15, 21, 24, 34, 36) showed little or no ACE2-blocking function. The BA.2 mAbs are somewhat less able to inhibit ACE2 binding than similarly potent early-pandemic mAbs.[4] Heavy- and light-chain gene usage is shown, compared with the gene usage we found in mAbs generated from early-pandemic infection, in Figure 2D and Table S2. 10/25 mAbs belonged to the IGHV1-69 gene family, a further expansion from that found within the Omicron BA.1 panel of mAbs (6/28) previously generated.[13] The public gene family IGHV3-53/66, which has been found on multiple occasions in SARS-CoV-2, was less prominent in the BA.2 set (3/25) than in the BA.1 set (9/28).

Somatic mutation in the BA.2 mAbs was greater than we have seen in any previous set of SARS-CoV-2 mAbs and was especially significant compared with the early-pandemic set (mean number of mutations: 10.9 vs. 4.6 and 7.2 vs. 4.3 for the light and heavy chains, respectively) (Figure 2E).

Neutralization assays show that all potent BA.2 mAbs cross-react with early-pandemic virus Victoria (IC50 < 100 ng/mL) (Figure 3). In contrast, from a previous set of mAbs isolated following primary Beta infection, only 3/18 (16.7%) of the potent Beta neutralizers (IC50 < 100 ng/mL) were able to neutralize Victoria, suggesting that the BA.2 mAbs are likely derived from memory B cells induced by vaccination.[14]

Epitope mapping of the mAb panel

We performed pairwise BLI competition measurements on the 25 potent RBD-binding BA.2 mAbs and several pre-pandemic and BA.1 mAbs of known binding position (Figures 4A and S1A) to obtain a 3D map of the binding positions of antibodies on the RBD[4] (Figure 4B). To understand if antibody responses have changed as SARS-CoV-2 has evolved, we show in Figures 5A and 5B the antibody-binding sites on the RBD surface for four sets of responses following the pandemic, and Figures 5C and 5D and Videos S1, S2, S3, and S4 display these as heat-maps to indicate the concentration of antibody across the RBD surface.

The BA.2 mAbs segregate into 3 adjacent clusters, two of which (left shoulder and neck in our anatomical definition of RBD topology[4]) are hotspots for the binding of potent mAbs against early-pandemic virus,[4,14] while the third cluster, which we term the right chest, spans between the neck and right flank epitopes in early-pandemic responses (note that in the early responses, right flank binders were not found to be potent [IC50 > 100 ng/mL]). In contrast, in early-pandemic and Beta responses, we find that potent antibodies are clustered into the three regions closest to the ACE2-binding site: the right shoulder, the neck, and the left shoulder. Beta responses are somewhat more evenly distributed, probably because of the significant reduction of the IGHV3-53/66 so-called public responses to the left shoulder epitope, which dominate early-pandemic responses (Figures 5C and 5D). Overall, the three BA.2 clusters are very similar to the three clusters found in the BA.1 mAb set[13] (Figures 5C and 5D).

The right chest cluster, the most populous in BA.2, has been characterized by binding of the BA.1 IGHV1-69 gene family antibodies.[13] However, for BA.2, only 4 of the 10 IGHV1-69 antibodies bind here (5, 17, 24, 34), in addition to 3/4 IGHV3-9 antibodies (10, 21, 28), which lie a little below the IGHV1-69 antibodies (Figure 4). The second cluster, neck, harbors the remaining five BA.2 IGHV1-69 antibodies. This cluster also matches a cluster of BA.1 antibodies.[13] The final cluster, left shoulder, includes the remaining IGHV3-9 antibody, BA.2-12, and the 3 IGHV3-53/3–66 antibodies (Figure 4). This cluster is diminished for BA.2 due to the relative paucity of effective IGHV3-53/66 antibodies. Note that in Figure 4, 42 refers to mAb Omi-42 isolated from a BA.1-infected case, which also binds more medially at the back of the RBD and is the only BA.1 mAb able to resist all the variants described in this article.

The relationship between the three BA.2 binding hotspots and the mutations in the recent variants is shown on the heatmap in Figures 5C and 5D. There is a strong correlation between the hotspots of antibody binding and the locations of mutations. In summary, Omicron antibodies show a shift in focus of the

Figure 2. Generation of BA.2 mAbs

(A) Sorting of BA.2-specific B cells.

(B) Proportion of BA.2 mAbs binding to RBD.

(C) ACE2 receptor blocking activity of mAbs.

(D) Gene usage in 25 potent BA.2 mAbs compared with potent antibodies produced following infection with early-pandemic virus, which have been previously reported.[4]

(E) Number of somatic mutations found in BA.2 mAbs compared with sets previously recovered from early-pandemic, Beta, and Omicron (BA.1) infections, which have been reported previously.[4,13,14]

A

Pseudovirus Neutralisation																							
mAbs	Victoria	Alpha	Beta	Gamma	Delta	BA.1	BA.1.1	BA.2	BA.2.12.1	BA.4/5	BA.4.6	BA.2.75	BA.2.75.2	BA.2.3.20	BJ.1	BF.7	BQ.1	BQ.1.1	BS.1	BA.2.10.4	BN.1	XBB	XBB.1
BA.2-24 (1-69)	0.015	0.009	0.002	0.003	10.000	0.010	0.008	0.004	10.000	10.000	10.000	0.002	0.004	10.000	10.000	10.000	10.000	10.000	10.000	0.008	10.000	10.000	10.000
BA.2-17 (1-69)	0.009	0.011	0.010	0.005	10.000	0.010	0.030	0.005	0.052	10.000	10.000	0.011	0.013	0.066	10.000	10.000	10.000	10.000	10.000	0.022	5.279	10.000	10.000
BA.2-34 (1-69)	0.004	0.002	0.001	0.002	0.029	0.033	4.176	0.002	0.333	1.515	10.000	0.007	1.880	10.000	10.000	10.000	10.000	10.000	10.000	0.051	10.000	10.000	10.000
BA.2-05 (1-69)	0.001	0.004	0.006	0.005	0.929	0.030	0.012	0.007	0.108	10.000	10.000	0.015	0.040	10.000	10.000	10.000	10.000	10.000	10.000	0.040	10.000	10.000	10.000
BA.2-15 (1-69)	0.003	0.006	10.000	10.000	0.004	0.062	0.029	0.033	0.210	10.000	10.000	0.070	0.899	10.000	10.000	10.000	10.000	10.000	0.986	10.000	10.000	10.000	10.000
BA.2-06 (1-69)	0.004	0.004	9.816	10.000	0.003	0.006	0.004	0.005	0.100	10.000	10.000	0.011	0.410	10.000	10.000	10.000	10.000	10.000	1.047	10.000	10.000	10.000	10.000
BA.2-02 (1-69)	0.002	0.002	1.609	1.386	0.004	0.002	0.002	0.002	0.049	10.000	10.000	0.001	0.159	3.180	2.306	5.554	7.262	6.416	0.276	10.000	0.599	10.000	10.000
BA.2-11 (1-69)	0.004	0.002	0.690	0.086	0.003	0.002	0.002	0.001	0.019	10.000	10.000	0.003	0.040	10.000	0.084	10.000	10.000	10.000	0.070	10.000	0.165	10.000	10.000
BA.2-16 (1-69)	0.006	0.007	0.155	0.105	0.003	0.003	0.003	0.002	0.011	10.000	10.000	0.004	0.016	1.112	0.068	10.000	10.000	10.000	0.036	4.104	0.197	4.566	4.604
BA.2-07 (1-69)	0.003	0.002	0.001	0.001	0.004	0.001	0.0004	0.002	0.003	0.002	0.003	0.001	0.002	0.004	0.003	0.005	0.009	0.005	0.014	0.004	0.003	0.008	0.011
BA.2-21 (3-9)	0.013	0.012	0.040	0.028	0.008	0.067	0.816	0.032	0.028	0.100	10.000	0.030	0.404	0.221	10.000	10.000	0.234	10.000	10.000	0.096	10.000	10.000	10.000
BA.2-28 (3-9)	0.034	0.010	0.020	0.034	0.007	0.043	0.023	0.032	0.016	0.133	10.000	0.032	1.517	0.767	10.000	10.000	2.553	10.000	10.000	0.054	10.000	10.000	10.000
BA.2-10 (3-9)	0.0003	0.0004	0.0004	0.001	0.001	0.001	0.0004	0.001	0.002	0.001	0.092	0.001	0.176	0.152	10.000	0.223	4.054	10.000	0.493	0.003	2.011	10.000	10.000
BA.2-12 (3-9)	0.003	0.001	0.003	0.001	0.003	0.004	0.003	0.003	0.004	2.731	3.000	0.004	10.000	0.007	0.008	0.531	10.000	7.069	0.019	10.000	0.006	10.000	10.000
BA.2-26 (3-53)	0.005	0.007	0.001	0.001	0.006	0.001	0.001	0.002	0.003	0.003	0.003	10.000	10.000	10.000	0.003	0.004	10.000	10.000	0.815	0.014	10.000	10.000	10.000
BA.2-23 (3-53)	0.012	0.008	0.002	0.003	0.009	0.005	0.005	0.005	0.006	0.005	0.004	0.211	0.199	0.120	0.012	0.003	0.654	0.749	0.095	0.008	5.012	0.915	0.667
BA.2-04 (3-53)	0.003	0.004	0.002	0.002	0.006	0.002	0.001	0.006	0.003	0.009	0.011	0.087	2.103	0.018	0.005	0.009	1.400	1.268	0.088	0.022	0.988	10.000	10.000
BA.2-09 (1-2)	0.007	0.005	10.000	10.000	0.003	0.003	0.003	0.001	0.001	0.558	0.774	0.004	10.000	10.000	1.722	0.283	0.802	0.288	0.004	10.000	2.183	10.000	10.000
BA.2-03 (1-2)	0.003	0.003	1.243	0.079	0.004	0.002	0.002	0.001	0.001	0.015	0.036	0.004	0.829	10.000	0.910	0.013	0.058	0.027	0.007	10.000	0.404	10.000	10.000
BA.2-30 (4-59)	0.008	0.003	0.012	0.009	0.017	1.596	10.000	0.006	0.045	10.000	10.000	0.670	10.000	10.000	10.000	10.000	10.000	10.000	10.000	10.000	10.000	10.000	10.000
BA.2-25 (4-59)	0.007	0.005	0.002	0.005	0.011	0.050	0.034	0.012	0.011	0.034	0.361	0.031	0.251	10.000	10.000	0.220	10.000	10.000	0.408	10.000	10.000	10.000	10.000
BA.2-36 (4-61)	0.005	0.002	0.001	0.004	0.001	0.007	0.466	0.007	0.010	0.064	10.000	0.014	1.315	10.000	10.000	10.000	10.000	10.000	10.000	0.026	10.000	10.000	10.000
BA.2-33 (4-61)	0.004	0.003	0.001	0.005	0.008	0.003	0.002	0.002	0.003	10.000	10.000	0.002	10.000	0.012	0.003	2.099	10.000	10.000	0.007	0.691	0.005	10.000	10.000
BA.2-13 (3-15)	0.012	0.020	0.003	0.003	0.024	0.006	0.474	0.008	0.005	0.007	4.595	0.018	10.000	10.000	10.000	0.341	0.013	10.000	4.698	0.009	10.000	10.000	10.000
BA.2-19 (3-48)	0.004	0.003	0.003	0.003	0.003	0.006	0.005	0.007	0.009	10.000	10.000	0.005	10.000	0.009	0.022	10.000	10.000	10.000	0.925	10.000	0.018	10.000	10.000

μg/mL
10.000
0.100
0.001

B

Pseudovirus Neutralisation																
	Victoria	BA.2	BA.4/5	BA.4.6	BA.2.75	BA.2.75.2	BA.2.3.20	BF.7	BQ.1	BQ.1.1	BJ.1	BS.1	BA.2.10.4	BN.1	XBB	XBB.1
AZD1061	0.002	0.008	0.015	10.000	0.021	10.000	10.000	10.000	10.000	10.000	10.000	10.000	0.072	10.000	10.000	10.000
AZD8895	0.001	1.333	10.000	10.000	0.008	10.000	0.007	10.000	10.000	10.000	0.085	10.000	10.000	0.024	10.000	10.000
AZD7442	0.001	0.008	0.065	10.000	0.017	10.000	0.026	10.000	10.000	10.000	0.228	10.000	0.098	0.068	10.000	10.000
REGN10987	0.002	0.616	10.000	10.000	10.000	10.000	10.000	3.925	10.000	10.000	10.000	10.000	10.000	10.000	10.000	10.000
REGN10933	0.001	10.000	10.000	10.000	10.000	10.000	8.624	10.000	10.000	10.000	10.000	10.000	10.000	1.954	10.000	10.000
ADG20	0.003	10.000	10.000	10.000	10.000	10.000	10.000	10.000	10.000	10.000	10.000	10.000	10.000	10.000	10.000	10.000
Ly-CoV555	0.002	10.000	10.000	10.000	10.000	10.000	10.000	10.000	10.000	10.000	10.000	10.000	10.000	10.000	10.000	10.000
Ly-CoV16	0.014	10.000	10.000	10.000	10.000	10.000	10.000	10.000	10.000	10.000	10.000	10.000	10.000	10.000	10.000	10.000
Ly-CoV1404	0.001	0.001	0.002	0.001	0.002	0.002	0.018	0.002	10.000	10.000	10.000	0.004	0.003	0.004	10.000	10.000
S309	0.079	0.638	0.689	1.029	0.202	0.465	0.977	0.801	0.662	0.751	0.839	2.696	0.410	10.000	0.455	0.142

μg/mL
10.000
0.100
0.001

Figure 3. Heatmap of IC50 neutralization titers
(A) BA.2 mAb panel.
(B) Commercial mAbs. Pseudoviral neutralization IC50 titers for indicated mAb against a panel of pseudoviruses expressing variant S sequences. All assays have been done at least twice. Commercial mAbs against Victoria, BA.2, BA.2.75, BA.4/5, and BA.4.6 previously reported are included for comparison.[24,27,28]
See also Figures S3 and S4.

antibody response, with BA.1 and BA.2 mAb panels converging on similar areas but with BA.2 somewhat more skewed from the early-pandemic response (Figures 5A–5D).

Structural analysis

To obtain more detailed insight into selected BA.2 mAbs, we determined the cryo-EM structure of the complex of the Delta-RBD with BA.2-23 and BA.2-36, along with the early-pandemic mAb-45[4] and EY6A[29] and also X-ray structures of the Delta RBD with BA.2-10 and EY6A, the Delta-RBD with BA.2-13 and C1 nanobody, and the Delta-RBD with BA.2-36 (Figures 6, S1B–S1D, and S2; Table S3).

BA.2-10 (IGHV3-9) binds at the right chest region of the RBD as expected from the competition mapping. It makes extensive interactions via complementarity-determining regions (CDRs) H3, L3, and L1 with residue V445 of the RBD and with R346 via CDRs H1 and H2, explaining its reduced neutralization of variants containing the R346T mutation (e.g., BA.4.6 and BA.2.75.2) and complete knockout by the BJ.1 variant, which contains both the R346T and V445P mutations (Figures 6A–6D).

BA.2-13 (IGHV3-15) also binds to the right chest region but higher and more toward the midline of the RBD than BA.2-10 and in a very different orientation so that its LC contact area on the RBD largely overlaps with the footprint of BA.2-10 HC. However, the contact area of BA.2-10 CDR-H3 overlaps the CDR-H3 contacts of BA.2-13. BA.2-13 interacts with RBD residues 346, 444–445, 450, and 452 (Figures 6E–6I). Variants containing the R346T mutation either seriously reduce or completely knock out neutralization of BA.2-13. BA.2.3.20, which contains K444R, N450D, and L452M mutations, also completely knocks out the activity of BA.2-13. Although BA.2-13 contacts RBD residues 452 and 484, it is not sensitive to mutations L452R or E484K, as it fully neutralizes Beta, Gamma, and Delta variants.

CellPress
OPEN ACCESS

A

Abs	BA2-2	BA2-3	BA2-4	BA2-5	BA2-6	BA2-7	BA2-9	BA2-10	BA2-11	BA2-12	BA2-13	BA2-15	BA2-16	BA2-17	BA2-19	BA2-21	BA2-23	BA2-24	BA2-25	BA2-26	BA2-28	BA2-30	BA2-33	BA2-34	BA2-36	BA2-38
BA2-2	0.16	0.10	0.09	0.11	0.03	0.15	0.06	0.64	0.18	0.20	0.04	0.19	0.18	0.12	0.26	0.15	0.17	0.07	0.21	0.13	0.08	1.20	0.16	0.13	0.14	0.16
BA2-3	0.12	0.07	0.04	0.06	0.00	0.11	-0.02	0.84	0.06	0.10	0.00	0.11	0.08	0.05	0.12	0.58	0.08	0.01	0.15	0.03	0.17	0.19	0.12	0.07	0.11	0.21
BA2-4	0.21	0.17	0.13	1.47	0.15	0.20	0.11	1.07	0.09	0.05	1.61	0.12	0.07	1.39	0.19	1.02	0.06	1.28	1.06	0.03	1.13	0.90	0.05	1.06	1.02	0.77
BA2-5	0.15	0.12	1.49	0.06	0.01	0.19	0.03	0.03	0.05	1.15	0.02	0.10	0.08	0.04	1.54	0.06	1.38	-0.01	0.14	1.64	0.00	0.08	1.30	-0.01	0.13	0.08
BA2-6	0.16	0.10	0.10	0.11	0.03	0.15	0.05	0.62	0.09	0.12	0.05	0.09	0.09	0.06	0.21	0.09	0.10	-0.04	0.15	0.05	0.02	0.17	0.08	0.12	0.17	0.22
BA2-7	0.11	0.00	0.02	-0.01	-0.03	0.02	-0.04	0.61	0.18	0.16	-0.06	0.22	0.18	0.14	0.18	0.03	0.10	-0.07	0.11	0.06	-0.03	0.10	0.02	-0.04	0.07	0.10
BA2-9	0.11	0.02	0.08	0.07	0.01	0.12	0.00	0.75	0.12	0.12	0.04	0.12	0.12	0.10	0.51	0.94	0.24	0.12	0.43	0.22	0.37	0.12	-0.06	-0.05	0.14	0.09
BA2-10	1.08	1.32	1.43	0.00	1.40	1.27	1.60	0.06	1.53	1.05	0.04	0.73	0.63	0.14	1.18	0.10	1.11	0.01	0.12	1.02	0.00	0.09	1.00	0.02	0.07	0.08
BA2-11	0.18	0.06	0.09	0.05	0.09	0.18	0.12	0.69	-0.02	-0.13	0.08	0.01	0.02	-0.03	0.14	0.00	0.04	-0.14	0.11	0.00	-0.04	0.11	0.01	-0.08	0.16	0.15
BA2-12	0.20	0.10	0.05	1.15	0.12	0.16	0.12	1.08	0.06	-0.05	0.88	-0.05	-0.08	1.15	-0.08	1.08	-0.12	2.05	1.18	-0.17	2.01	1.18	-0.33	1.66	1.01	1.39
BA2-13	0.12	0.06	1.41	0.06	0.07	0.17	0.09	0.10	-0.02	1.08	0.06	0.17	0.12	0.08	1.29	0.11	1.33	0.02	0.20	1.29	0.05	0.15	0.96	0.04	0.10	0.13
BA2-15	0.19	0.11	0.12	0.10	0.09	0.22	0.12	0.73	0.01	-0.05	0.17	0.01	0.06	0.00	0.15	-0.02	0.01	-0.09	0.11	0.06	0.00	0.09	0.03	-0.04	0.22	0.16
BA2-16	0.18	0.08	0.07	0.08	0.09	0.18	0.12	0.63	0.02	-0.08	0.12	0.06	-0.12	-0.10	0.18	0.08	0.11	0.00	0.18	0.07	0.03	0.10	0.01	-0.05	0.18	0.15
BA2-17	0.12	0.05	1.39	0.04	0.06	0.14	0.10	0.14	-0.03	1.15	0.08	0.00	-0.10	0.00	1.58	-0.04	1.95	-0.12	0.12	1.76	-0.06	0.05	1.39	-0.02	0.11	0.08
BA2-19	0.26	0.12	0.19	1.54	0.21	0.18	0.51	1.18	0.14	-0.08	1.29	0.15	0.18	1.58	-0.06	1.88	0.20	1.13	1.51	-0.26	2.45	1.48	-0.11	2.02	1.08	1.77
BA2-21	0.15	0.58	1.02	0.06	0.09	0.03	0.94	0.10	0.00	1.08	0.11	-0.02	0.08	-0.04	1.86	-0.17	0.97	0.24	0.09	1.76	-0.13	0.01	1.50	-0.06	0.16	0.02
BA2-23	0.17	0.08	0.06	1.38	0.10	0.10	0.24	1.11	0.04	-0.12	1.33	0.01	0.11	1.95	-0.11	2.04	0.10	1.08	1.30	0.07	0.94	0.99	0.06	1.04	1.05	0.92
BA2-24	0.07	0.01	1.28	-0.01	-0.04	-0.07	0.12	0.01	-0.14	2.05	0.02	-0.09	0.00	-0.12	2.37	-0.21	0.94	0.14	0.02	1.16	0.15	0.27	1.12	0.13	0.06	0.34
BA2-25	0.21	0.15	1.06	0.14	0.15	0.11	0.43	0.12	0.11	1.18	0.20	0.11	0.18	0.12	2.34	0.05	0.97	0.33	0.15	1.53	0.03	0.08	1.62	0.08	0.23	0.14
BA2-26	0.13	0.03	0.03	1.64	0.05	0.06	0.22	1.02	0.00	-0.17	1.29	0.06	0.07	1.76	-0.26	1.76	0.07	1.16	1.53	0.11	1.24	1.33	0.11	1.35	1.11	1.04
BA2-28	0.08	0.17	1.13	0.00	0.02	-0.03	0.37	0.00	-0.04	2.01	0.05	0.00	0.03	-0.06	2.45	-0.13	0.94	0.15	0.03	1.24	0.00	0.20	1.30	0.13	0.09	0.25
BA2-30	1.20	0.19	0.90	0.08	0.17	0.10	0.12	0.09	0.11	1.18	0.15	0.09	0.10	0.05	1.48	0.01	0.99	0.27	0.08	1.33	0.20	0.19	1.19	0.27	0.18	0.15
BA2-33	0.16	0.12	0.05	1.30	0.08	0.02	-0.06	1.00	0.01	-0.33	0.96	0.03	0.01	1.39	-0.11	1.50	0.06	1.12	1.62	0.11	1.30	1.44	0.15	1.32	0.90	1.51
BA2-34	0.13	0.07	1.06	-0.01	0.12	-0.04	-0.05	0.02	-0.08	1.66	0.04	-0.04	-0.05	-0.02	2.02	-0.06	1.04	0.13	0.08	1.35	0.13	0.13	1.48	0.16	0.06	0.12
BA2-36	0.13	0.06	1.00	0.01	0.09	-0.03	-0.06	0.01	-0.05	0.97	0.03	0.00	-0.04	-0.01	2.02	-0.08	1.01	0.14	0.07	1.44	0.09	0.09	1.45	0.10	0.05	0.11
BA2-38	0.16	0.21	0.77	0.08	0.22	0.10	0.09	0.08	0.15	1.39	0.13	0.16	0.15	0.08	1.77	0.02	0.92	0.34	0.14	1.04	0.25	0.23	1.26	0.35	0.18	0.23
Omi-12	-0.02		-0.24	0.93	-0.18	-0.12		0.87	-0.77	-0.89	0.94	-0.53	-0.53	0.64	-0.58	0.61	-0.29	0.77	1.08	-0.13	0.93	0.83	-0.08	0.87	0.86	0.90
Omi-42	0.42	0.22	0.12	1.45	0.23	1.08	0.08	1.43	0.30	-0.05	1.61	0.13	0.45	1.34	0.03	1.40	0.15	1.03	1.22	0.22	1.20	1.26	0.29	1.28	1.21	1.64
EY6A	0.77	0.55	0.71	0.56	0.71	0.61	0.90	0.58	0.81	0.84	0.74	0.84	0.62	0.56	0.77	0.49	0.85	0.62	0.56	0.91	0.74	0.66	0.85	0.77	0.81	0.72
S309	0.67	0.43	0.82	0.19	0.33	0.97	0.02	-0.11	-0.35	0.27	-0.30	-0.38	-0.11	-0.62	0.13	-0.03	0.33	-0.05	-0.43	0.54	-0.01	-0.19	0.46	-0.04	-0.15	-0.20
COVOX-278	-0.22	-0.11	0.79	-0.19	0.03	-0.20	-0.38	-0.13	-0.58	1.24	-0.08	-0.63	-0.38	-0.28	1.49	-0.34	0.97	0.02	-0.20	1.10	0.05	-0.04	1.05	0.07	-0.03	-0.06
COVOX-58	-0.24	0.54	0.91	-0.21	-0.02	-0.25	0.79	-0.25	-0.61	1.48	-0.09	-0.56	-0.37	-0.31	1.41	-0.39	1.17	-0.05	-0.25	1.26	-0.02	-0.11	1.07	-0.04	-0.08	-0.09
COVOX-45	0.78	0.72	0.58	0.44	0.61	0.60	0.93	0.18	0.85	0.82	0.62	0.72	0.59	0.07	0.86	-0.35	0.70	0.90	0.90	0.87	0.10	0.53	0.69	0.69	0.60	0.57
AZD1061	0.25	0.37	1.36	0.24	0.31	0.34	0.22	0.25	0.13	1.81	0.15	0.14	0.07	0.18	2.04	0.13	1.43	0.40	0.18	1.37	0.35	0.25	1.19	0.36	0.30	0.24
AZD8895	0.81	0.45		1.15	0.64	1.04	0.70	1.06	0.56	0.66	1.19	0.51	0.34	0.96	0.59	1.01	0.55	0.89	0.90	0.68	0.91	0.88	0.68	0.94	0.98	0.89

B

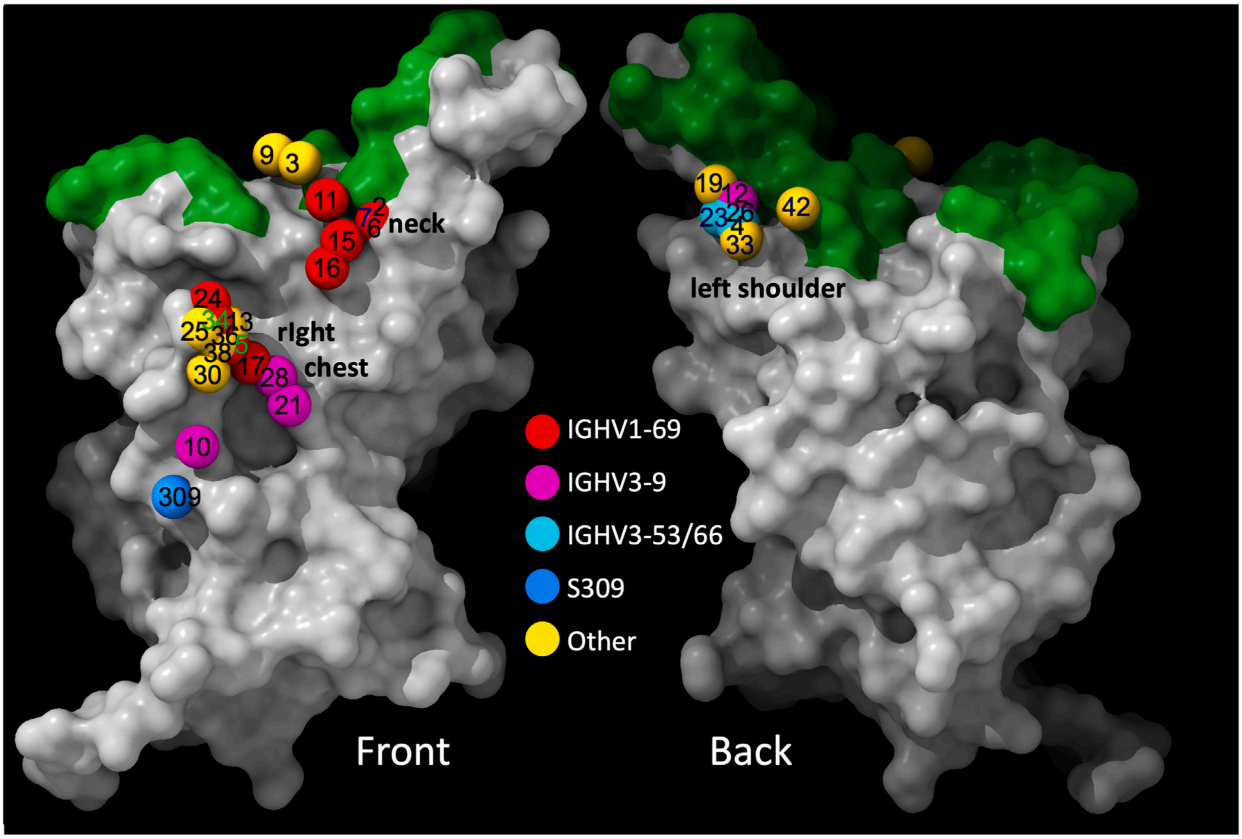

(legend on next page)

BA.2-36 (IGHV4-61) binds to the right chest region in a similar position and orientation to BA.2-13 (Figures 6J–6N). It also contacts RBD residues 346, 444, 450, and 452, explaining its similar variant neutralization profile to BA.2-13, with neutralization seriously reduced or completely knocked out for variants containing the R346T mutation and by BA.2.3.20.

BA.2-23 is an IGHV3-53 family antibody and binds the left shoulder in the expected position. Note that most potent IGHV3-53/3-66 antibodies bind in essentially the same way (although a second binding mode has also been observed),[30] and yet they vary in their sensitivity to mutations, as we have discussed before.[13] For the recent variants, a key mutation is residue 486, where the change from F to V has a negative effect and the change to the even smaller and polar S side chain is more deleterious (Figures S2 and S3; Table S1). The N460K mutation also impacts the binding of this heavy-chain family. Since IGHV3-53/3-66 form a major public response in SARS-CoV-2 infection, it is likely that BA.2.75.2 has acquired F486S and N460K to evade them (Table S1).

Widespread escape from mAb neutralization

To test the resilience of the BA.2 mAbs to newly described BA.2 variants, we constructed a panel of pseudoviruses expressing S gene sequences for the variants and measured neutralization using BA.2 as comparison (Figures 3A and S3A).

On BA.4, the activity of 11/25 mAbs was knocked out, and only 10 retained IC50 titers $\leq$100 ng/mL. Activity of 8/10 IGHV1-69 mAbs was knocked out completely on BA.4, likely due to mutations 452, 486, and 493 (reversion) on the neck and left shoulder of the RBD (Figure 1B). Activity of 3 further mAbs was lost with the addition of the R346T mutation in BA.4.6 including the 2 IGHV3-9 mAbs BA.2-21 and BA.2-28, which map to the chest, overlapping residue 346 (Figure 1B).

BA.2.75 had a minor effect on neutralization, with only BA.2-26 (IGHV3-66) showing near knockout of activity. The effect of BA.2.75.2 was much more marked: the activity of 7/25 mAbs was completely lost, with only 6/25 mAbs retaining IC50 values <100 ng/mL, and the 2 IGHV3-53 mAbs BA.2-04 and BA.2-26 were knocked out, showing that BA.2.75.2 has evolved to evade this very common public response.[7] The mutation F486S is a radical change and, together with N460K, is likely responsible for these effects, mapping close to the affected mAb (Figures 1B and 4).

BA.2.3.20, which has a number of mutations across the top of the shoulders, neck, and at the back of the RBD (Figures 1A and 1B), also caused large disruption: 13/25 mAbs were knocked out, with only 6/25 retaining IC50 activity at <100 ng/mL. Finally, for BJ.1, which has a different set of mutations across the top of the RBD, the activity of 13/25 mAbs was completely knocked out, with only 9/25 retaining IC50 activity at <100 ng/mL.

The activity of most mAbs was reduced or completely knocked out against BQ.1, BQ.1.1, XBB, and XBB.1, while the activity of some mAbs was preserved on BN.1 and BA.2.10.4. Overall, it appears that mutational change in SARS-CoV-2 since the emergence of BA.2 has led to mutations that knock out or severely reduce activity of almost all BA.2-directed mAbs; only BA.2-04, BA.2-07, and BA.2-23 did not suffer a complete knockout of activity with any of the pseudoviruses tested, and only BA.2-07 maintained full activity (Figures 3A and S3).

Finally, we returned to a panel of mAbs made following BA.1 infection[13] and found that there was a similar attrition of mAb activity with the new variants (Figures S3B and S4), with XBB leading to the most extreme escape. Activity of all 9 IGHV3-53/66 mAbs was reduced >100-fold with complete knockout of activity in 5/9 by BA.2.75.2. Only a single mAb, Omi-42, was unaffected by all variants. Omi-42 is unusual, as it binds at the back of the left shoulder of the RBD (Figure 4, right panel)[13] in a region that has not yet been targeted for mutation by the set of newly emerging BA.2 variants, perhaps because of the relative rarity of antibodies binding in this region (Figures 1B and 4).

Escape from therapeutic mAbs

We tested the activity of a panel of mAbs that have been developed for clinical use.[31–34] Many of these have already been severely impacted by a number of variants. Activity of all mAbs was knocked out by one or more variants including LY-CoV1404, which was inactive on BJ.1 and XBB probably due largely to the V445P mutation (Figures S3C and S2I).[16] LY-CoV1404 binds at the side of the right shoulder of the RBD, making extensive interactions with residues K444 and V445 (PDB: 7MMO; Figures S2G–S2I), and the K444T mutation in BQ.1 likely knocks out activity of LY-CoV1404.

Severe knockdown of serum neutralization titers

In a final series of experiments, we tested neutralization on serum collected 28 days following a third dose of Pfizer BNT162b2 vaccine[35] and in cases infected with BA.1, BA.2, and BA.4/5 (the characteristics of these subjects are described in the STAR Methods).

For serum obtained 28 days following BNT162b vaccination, neuralization titers to BA.2.75.2 and XBB showed large reductions (Figure 7A), 16.6- and 20.9-fold ($p < 0.0001$), respectively, compared with BA.2. In contrast to BA.2.75.2, BA.2.75 showed only a modest reduction compared with BA.2. There were also large reductions in titers to BQ.1 and BA.2.3.20, 11.4- and 7.7-fold, respectively ($p \leq 0.0001$) compared with BA.2. For BJ.1, titers were reduced 3.6-fold compared with BA.2 ($p < 0.0001$), but reductions were less marked compared with BA.2.75.2 and BA.2.3.20.

Figure 4. Result of BLI competition mapping of BA.2 mAbs

(A) Competition ratio results (using *Mabscape*; see STAR Methods). Numbers close to 0 represent complete competition between pairs of antibodies, and numbers close to 1 mean no competition. Cells are colored as red, yellow, and green, with the values ranging from 0 to 1. Antibodies with known structures (Omi-12, Omi-42, EY6A, S309, COVOX-45, COVOX-58, COVOX-278, AZD1061, and AZD8895) were used as references.

(B) RBD surface representation with ACE2-binding site in green and balls corresponding to center of gravity of mapped potent BA.2 mAbs colored according to variable gene usage.

See also Figure S1.

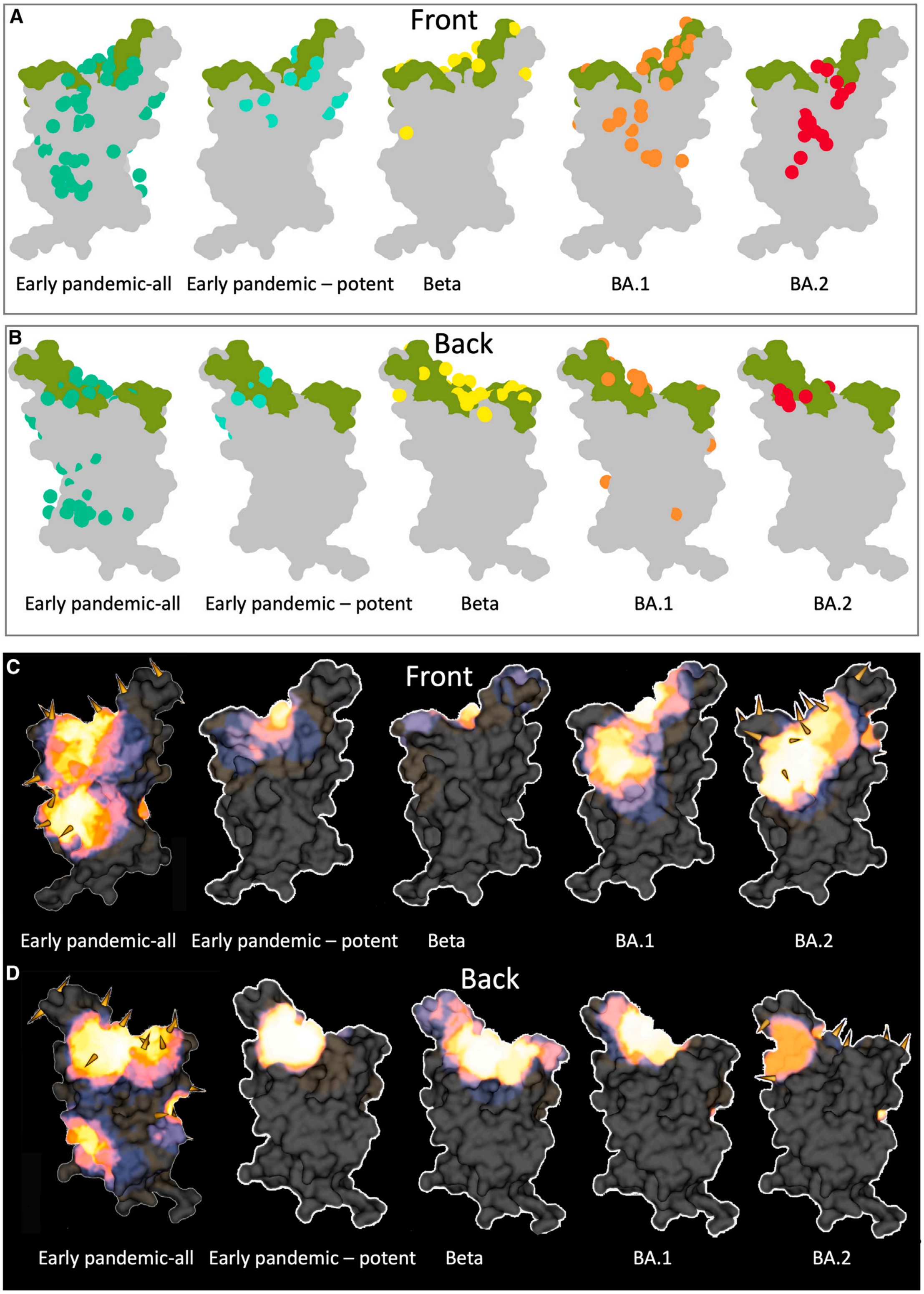

Figure 5. Comparison of mAb binding to RBD

(A and B) Front and back views of *Mabscape* antibody maps from early-pandemic, Beta, BA.1, and BA.2 antibody panels. Early pandemic all represents the full set of antibodies, irrespective of neutralization potency, and all other panels show potent mAbs (IC50 < 100 ng/mL). The RBDs are shown surface rendered (gray) with the ACE2 footprint in green.

(legend continued on next page)

Next, we tested serum taken following BA.1, BA.2, or BA.4/5 infection; all were vaccine breakthrough infections, barring one unvaccinated case of BA.4 (Figures 7B–7D). In all cases, new variant Omicron lineages showed reduced neutralization titers compared with BA.2 and BA.4/5, but the reductions were generally less compared with those seen with BNT162b2 vaccine serum, presumably due to the higher levels of antibody to the Omicron lineage found in these sera. The reduction in neutralization of XBB, which showed the greatest escape from BA.2 mAbs described above, was particularly marked with 22-, 45-, and 21-fold reduction in titers compared with BA.2 using BA.1, BA.2, and BA.4/5 serum, respectively.

Overall, in line with the observations on the set of mAbs described above, there were large reductions in neutralization titers against most BA.2 sub-lineages, particularly BA.2.75.2, BA.2.3.20, BQ.1, and XBB, suggesting that they have been selected to escape pre-existing immunity to vaccines or earlier waves of SARS-CoV-2 infection. Titers will be expected to wane considerably from those shown here for samples taken quite soon after vaccination or infection, meaning the newly emerging BA.2 variants may fuel future waves of SARS-CoV-2 infection; indeed, BQ.1/BQ.1.1 and XBB/XBB.1/XBB.1.5 are becoming the dominant Omicron sub-lineages in several regions (https://cov-spectrum.org/explore/World/AllSamples/Past6M).

DISCUSSION

Before the emergence of Omicron, RBD mutations were small in number: 1 in Alpha, 2 in Delta, and 3 in Beta and Gamma. These may in part have been selected for increased ACE2 receptor binding, although they also led to a degree of antibody evasion, which is most pronounced for Beta.[9,10,36,37] The arrival and rapid spread of Omicron BA.1 in November 2021 surprised many in the field, as it contained so many mutations, particularly in the RBD and NTD.

It is likely that evolution of SARS-CoV-2 Omicron is now primarily driven by extreme pressure to escape antibody responses in vaccinated and/or naturally infected individuals, with compensatory mutations to maintain or increase ACE2 affinity. There has been a succession of changes to the RBD with evidence for convergent evolution. Now that the majority of the population is either vaccinated or has been exposed, herd immunity is exerting huge pressure for immune escape. Many mutations are occurring on the edge of the ACE2-binding footprint to throw off potent antibodies that block ACE2.[3] The RBD shows a remarkable degree of plasticity, seemingly able to accommodate wide-ranging changes while still maintaining sufficient affinity for ACE2, similar to that shown for the human seasonal coronavirus 229E.[38] Taking all the new Omicron variants (Figure 1A), the ~200 residue RBD bears 29 mutated residues compared with the early-pandemic virus; this extraordinary level of variation, focused on the antigenic sites and leading to such effective antigenic escape, would conventionally lead to these viruses being classified as different serotypes.[39] What is even more remarkable, and what may be of some concern, is that this level of variation is focused on the part of the virus whose job it is to attach to cells and initiate infection, raising the possibility of inadvertent receptor switching,[40] which would have completely unpredictable results (already seen to date in a far more limited sense with variation altering the host range of the virus).[41] The plasticity of the RBD at and around the small ACE2-binding site means that rather than being a conserved Achilles heel for SARS-CoV-2, it is proving to be quite the opposite, giving considerable opportunity to throw off huge chunks of the antibody response through a succession of mutations.

We find, by analysis of a panel of antibodies generated from vaccinees suffering BA.2 breakthrough infections, that the focusing of potently neutralizing antibodies has shifted from that observed in early-pandemic responses and resembles that reported for BA.1.[13] BA.2 antibodies cluster tightly in two of the three regions characteristic of potent early-pandemic responses, although mutations have knocked out many of the so-called "public" responses, including gene families IGHV3-53/66, diminishing the potent left shoulder responses. There is a third more extensive binding patch at the front of the RBD, the right chest, which spans between the right flank and neck regions identified in the early-pandemic responses, extending down almost as far as the S309 therapeutic antibody on the right flank. S309 does not compete with ACE2, and a general feature is that many of the potent BA.2 antibodies are much further from the ACE2-binding site and, as a result, are less strongly competitive with ACE2 than early-pandemic antibodies (Figure 2C). In contrast to the potent BA.2 chest binders identified here, the early-pandemic right flank antibodies, which bind nearby, were generally weakly neutralizing. These shifts in binding patterns are seen in the heatmaps of antibody binding between antibody responses produced successively through the pandemic (Figures 5A–5D).

The changed pattern of potent antibody binding is likely because almost all antibodies seem to be derived from responses induced by vaccination (and sometimes also SARS-CoV-2 infection). In support of this, every member of our set of potent antibodies to BA.2 potently cross-reacted with Wuhan-related strains and was likely originally induced by vaccination, since all donors were triple vaccinated. It is not clear whether the cross-reacting antibodies were fully matured before BA.2 infection or whether affinity maturation of lower affinity clones occurred in response to BA.2 infection. The panel probably contains a mix of both, and the strong representation of weaker neutralizing right flank antibodies in the early responses suggests that the chest binders that overlap these may have been matured to higher affinity and selected because this portion of the RBD has been subjected to less mutational change in BA.1 and BA.2.

(C and D) Heatmaps of surface occupation of RBD by early-pandemic, Beta, BA.1, and BA.2 antibody panels by iron heat colors (black > blue > red > orange > yellow > white hot) according to the relative level of antibody contact, calculated for each surface vertex as the number of antibodies within a 10 Å radius. BA.1 mutations are shown by the spikes. Only the early-pandemic-all panels and the sub-variant substitutions discussed here are shown mapped onto BA.2. Early-pandemic, Beta, and BA.1 data are taken from Dejnirattisai et al.,[4] Nutalai et al.,[13] Liu et al.[14]
See also Figure S1.

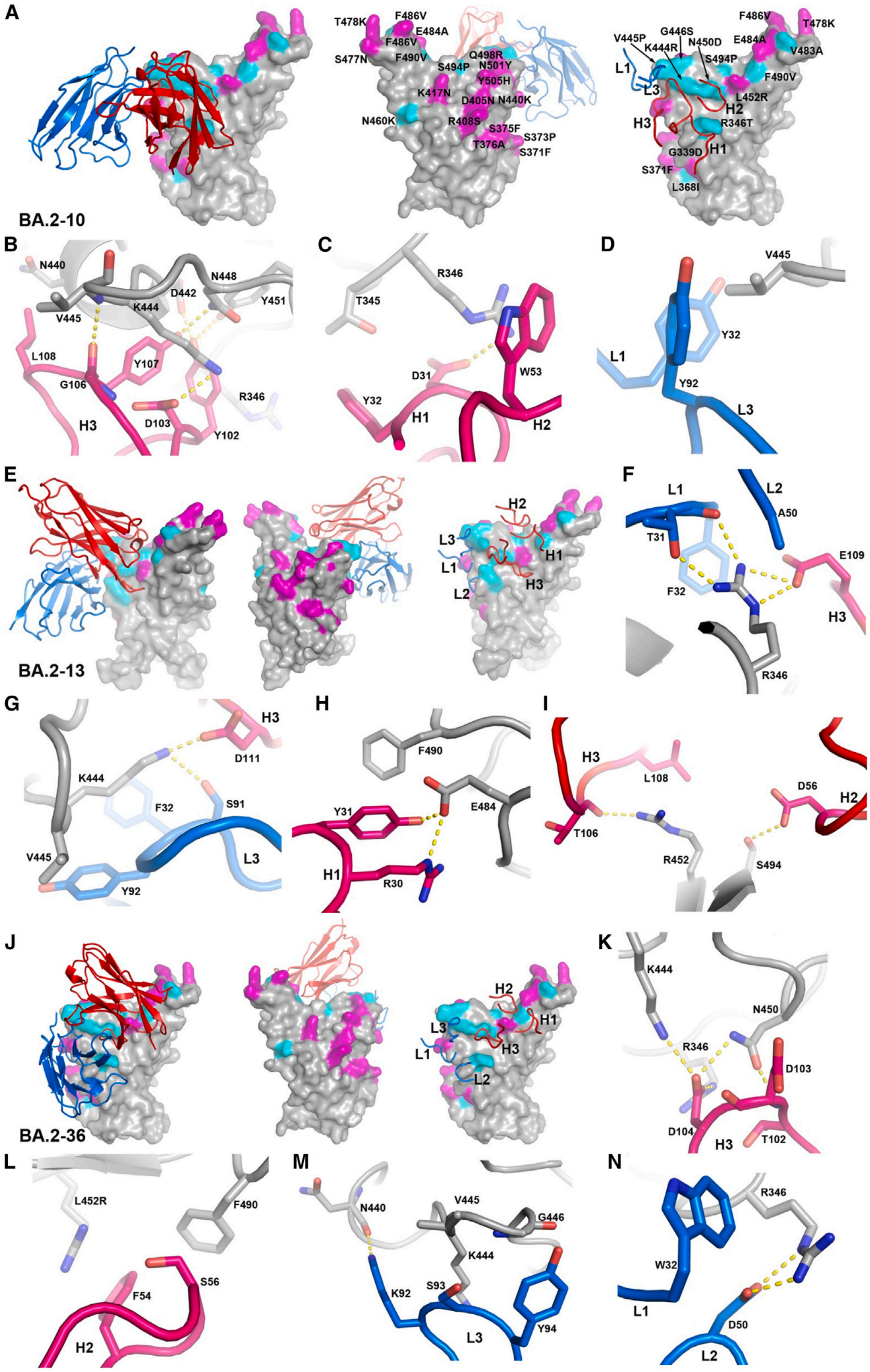

(legend on next page)

The change away from antibodies binding more directly on the ACE2 footprint is highlighted by the substantial role in early responses played by public gene families such as IGHV3-53/66,[7,42] IGHV1-69, IGHV3-9, and IGHV1-58.[13] Thus, in this article, the BA.2 IGHV1-69 mAbs are almost all knocked out by BA.4. Finally, the few members of the most frequently discovered gene family of all, IGHV3-53/66, that are active against BA.2 are severely affected by the F486S mutation in BA.75.2, as are the larger number of IGHV3-53/66 mAbs isolated following BA.1 infection. The refocusing is therefore explained by the evolution of the virus to the point of BA.1 and BA.2; however, there is no reason to think that residues on the right chest are required to be conserved. Indeed, the mutations in the recent sub-lineages are found on the edge or outside of the ACE2 epitope, deployed so as to effectively escape all three of the hotspots we see in BA.1 and BA.2 responses.

In summary, continuous attrition of the serological response and reliance on vaccine-induced clones to repopulate it is likely to reduce the memory pool and may limit the refocusing of the response required in the face of antigenic escape, increasing vulnerability. It is telling that the mutations to BA.2 described here target the regions used to bind 25 potent mAbs, rendering all but BA.2-07 compromised for at least one variant.

The situation is the same for the commercial mAbs, which largely bind to conserved hotspots, leading to failure of all therapeutic candidates to at least one variant. New approaches may be required to discover potent mAbs that have unusual binding sites that are not under such selective pressure if a pipeline of therapeutic/prophylactic candidates is to be maintained.

Now that herd immunity from vaccination and/or natural infection is high, there is intense pressure to escape from the antibody response, meaning it is likely we will see successive waves of infection as the virus continues to evolve. However, background immunity, perhaps led by the T cell response, which is less sensitive to mutations, will probably maintain protection against severe disease for the majority. Further monitoring of the antibody response throughout successive waves will tell us whether there is increasing attrition of polyclonality. However, as observed for BA.1, somatic mutation will probably enable the repair of some antibodies[13]; for instance, it seems unlikely that this is the end of the road for neutralization using the VH3-53/66- or VH1-69-binding sites.

Finally, many people are now receiving bivalent vaccine boosters containing the spike of both Wuhan and one Omicron BA.1 or BA.4/5. The data presented here, where all BA.2 mAbs cross-react with early-pandemic viruses, suggests that the Omicron component may mount a BA.1 or BA.4/5 response using B cell clones recalled from the original vaccine response rather than eliciting a primary response to BA.1 or BA.4. Finally, if recall responses are largely responsible for the population of responses on reinfection, is it possible that the structure of the response to reinfection is in part molded by the sequence of the priming virus or the vaccine reflecting original antigenic sin or imprinting?

Limitations of the study

The neutralization assays presented here are performed *in vitro* and may underestimate *in vivo* neutralization where antibody-dependent cell-mediated cytotoxicity and complement will be present.

CONSORTIA

The members of the OPTIC consortium are Christopher Conlon, Alexandra Deeks, John Frater, Siobhan Gardiner, Anni Jämsén, Katie Jeffery, Tom Malone, Eloise Phillips, Barbara Kronsteiner-Dobramysl, Priyanka Abraham, Sagida Bibi, Teresa Lambe, Stephanie Longet, Tom Tipton, Miles Carrol, and Lizzie Stafford.

STAR★METHODS

Detailed methods are provided in the online version of this paper and include the following:

- KEY RESOURCES TABLE
- RESOURCE AVAILABILITY
  - Lead contact
  - Materials availability
  - Data and code availability
- EXPERIMENTAL MODEL AND SUBJECT DETAILS
  - Bacterial strains and cell culture
  - Sera from BA.1 infected cases, study subjects
  - Sera and PBMC from BA.2 infected study subjects
  - Sera from BA.4/5 infected cases, study subjects
  - Sera from Pfizer vaccinees
- METHOD DETAILS
  - Isolation of BA.2 S-specific single B cells by FACS
  - Cloning and expression of BA.2 S-specific human mAbs
  - ACE2 binding inhibition assay by ELISA
  - Pseudovirus plasmid construction and lentiviral particle production

Figure 6. Structures of Delta-RBD complexes with BA.2-10, BA.2-13, and BA.2-36

(A) Binding position and orientation of BA.2-10 viewed from the front (left panel) and back (middle panel) of the RBD, and positions of the CDRs that have contact with the RBD (right panel). Only Vh (red) and Vl (blue) domains of the Fab are shown as ribbons for clarity. RBD is drawn as gray surface representation with BA.4 mutation sites highlighted in magenta, and the additional mutation sites of all variants shown in Figure 1A are shown in cyan.

(B–D) Details of BA.2-10 and RBD interactions. The side chains of the RBD and Fab Vh and Vl are shown as gray, red, and blue sticks, respectively. The yellow broken bonds represent hydrogen bonds or salt bridges.

(E and J) Complex of (E) Delta-RBD/BA.2-13 and (J) Delta-RBD/BA.2-36. The drawing style and color scheme are as in (A).

(F–I and K–N) Contact details between RBD and BA.2-13 and RBD and BA.2-36, respectively. The drawing style and color scheme are as in (B)–(D). Antibodies bind both Delta and BA.2 RBDs well; however, mutations from early pandemic to Delta are T478K and L452R (in BA.2, the T478K change is present, while L452R is not). Additional changes in BA.2 are G339D, S371F, S373P, S375F, T376A, D405N, R408S, K417N, N440K, S477N, T478K, E484A, Q493R, Q498R, N501Y, and Y505H.

See also Figures S1 and S2 and Table S3.

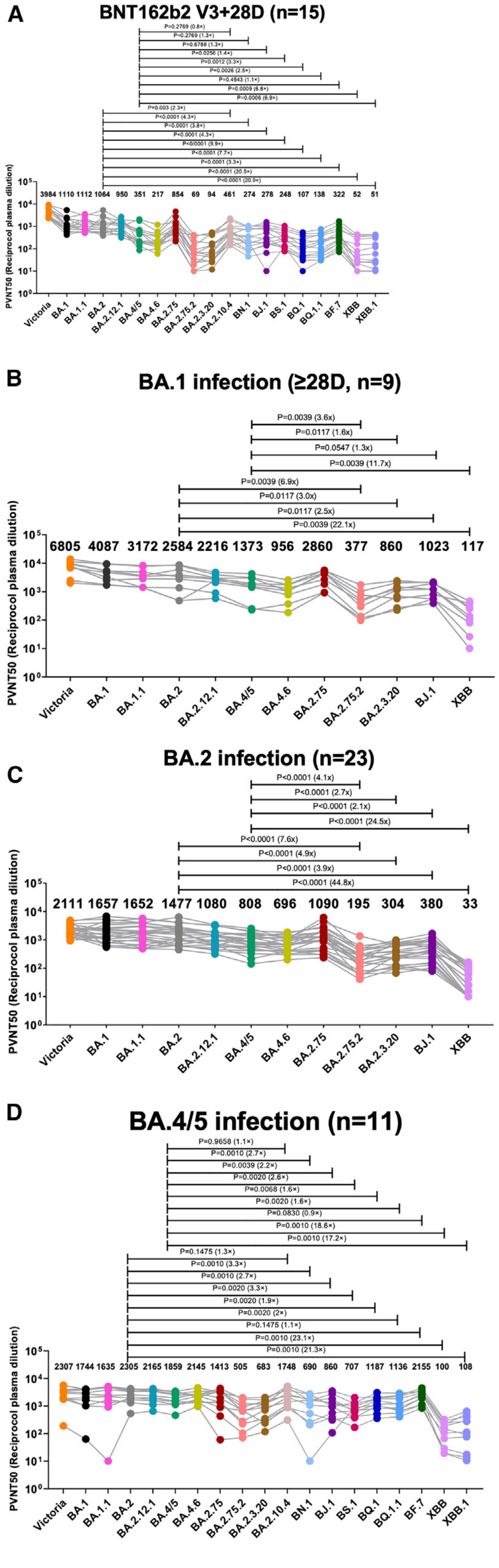

Figure 7. Serum neutralization IC50 titers (fold dilution) of lentivirus pseudotyped with the S gene of the indicated BA.2 sub-lineages

(A and B) Serum obtained 28 days following the third dose of BNT162b2 vaccine (n = 15) or following infection with (B) BA.1 (n = 10 all vaccinated) taken 28–55 days following diagnosis median 41.5.

(C and D) BA.2 (n = 23 all vaccinated) taken 12–43 days following diagnosis median 29 or (D) BA.4/5 (n = 11 all but one vaccinated) taken 23–48 days following diagnosis median 38 days. Geometric mean titers are shown above each column. The single unvaccinated serum shows the lowest reactivity to BA.4/5 in (D). The Wilcoxon matched-pairs signed rank test (C and D) and Mann-Whitney test were used and two-tailed p values calculated. Data for BNT162b2-vaccinated sera and BA.1 infection sera against Victoria, BA.1, BA.1.1, BA.2, BA.4/5, BA.4.6, BA.2.75, and BA.2.12.1 previously reported are included for comparison.[23,24,27,28] All assays have been done with the number of biological replicates indicated in the brackets.

- ○ Pseudoviral neutralization test
- ○ RBD production for structural analysis
- ○ IgG mAbs and Fabs production
- ○ Competition assays of anti-Omicron BA.2 RBD mAbs
- ○ Antibody mapping to RBD surface
- ○ Crystallization, X-ray data collection and structure determination
- ○ Cryo-EM structure determination
- ○ Phylogenetic tree
- ● QUANTIFICATION AND STATISTICAL ANALYSIS

SUPPLEMENTAL INFORMATION

Supplemental information can be found online at https://doi.org/10.1016/j.celrep.2023.112271.

ACKNOWLEDGMENTS

This work was supported by the Chinese Academy of Medical Sciences (CAMS) Innovation Fund for Medical Science (CIFMS), China (grant number: 2018-I2M-2-002), to D.I.S. and G.R.S. We are also grateful for support from Schmidt Futures, the Red Avenue Foundation, and the Oak Foundation. H.M.E.D. and J.R. are supported by Wellcome (203141/Z/16/Z and 101122/Z/13/Z respectively) and D.I.S. and E.E.F. by the UKRI MRC (MR/N00065X/1). D.I.S. and G.R.S. are Jenner Investigators. This is a contribution from the UK Instruct-ERIC Centre. A.J.M. is an NIHR-supported Academic Clinical Lecturer. This work is supported by the Oxford Biomedical Research Centre and an Oxfordshire Health Services Research Committee grant to A.J.M. The Wellcome Center for Human Genetics is supported by the Wellcome Trust (grant 090532/Z/09/Z). The computational aspects of this research were supported by the Wellcome Trust Core Award grant number 203141/Z/16/Z and the NIHR Oxford BRC and we acknowledge the Wellcome Human Genetics Flow Cytometry Facility. Electron microscopy provision was provided through the OPIC electron microscopy facility, a UK Instruct-ERIC Centre, which was founded by a Wellcome JIF award (060208/Z/00/Z) and is supported by a Wellcome equipment grant (093305/Z/10/Z). This work was supported by the UK Department of Health and Social Care as part of the PITCH (Protective Immunity from T cells to Covid-19 in Health workers) consortium, the UK Coronavirus Immunology consortium (UK-CIC), and the Huo Family Foundation. We acknowledge the Diamond Light Source for time on Beamline I03 under proposal lb27009 for COVID-19 Rapid Access. E.B. and P.K. are NIHR Senior Investigators, and P.K. is funded by WT222426/Z/21/Z and NIH (U19 I082360). S.J.D. is funded by an NIHR Global Research Professorship (NIHR300791). The Sheffield Teaching Hospitals Observational Study of Patients with Pulmonary Hypertension, Cardiovascular and Other Respiratory Diseases (STH-ObS) was supported by the British Heart Foundation (PG/11/116/29288). We gratefully acknowledge financial support from the UK Department of Health

via the Sheffield NIHR Clinical Research Facility award to the Sheffield Teaching Hospitals Foundation NHS Trust. T.P.P. is funded by the G2P-UK National Virology Consortium funded by the MRC (MR/W005611/1).

AUTHOR CONTRIBUTIONS

A.D.-G., R.D., C.L., R.N., P.S., M.S., G.R.S., and J.M. generated and characterized mAbs. A.D.-G., R.D., C.L., and R.N. generated pseudoviruses and performed neutralization tests. D.Z., J.R., N.G.P., M.A.W., and D.R.H. prepared the crystals and enabled and performed X-ray data collection. H.M.E.D. performed cryo-EM analyses. J.R., E.E.F., H.M.E.D., and D.I.S. analyzed the structural results. D.Z. and J.H. prepared the RBDs. D.Z. performed BLI competition assays. N.T. provided materials. T.P.P. and C.R. assisted with analysis of new BA.2 sub-lineages, C.R. made the phylogenetic tree. H.M.G. wrote *Mabscape* and performed mapping and cluster analysis, including sequence and antigenic space analyses. T.I.d.S., M.P., T.A.H.N., H.H., A.J.M., D.S., T.G.R., P.K., E.B., and S.J.D. assisted with patient samples and vaccine trials in the OPTIC Healthcare Worker and the Sheffield STH-Obs studies. E.B., S.J.D., and P.K. conceived the study of vaccinated healthcare workers and oversaw study and sample collection/processing. G.R.S., and D.I.S. conceived the study and wrote the initial manuscript draft with other authors providing editorial comments. All authors read and approved the manuscript.

DECLARATION OF INTERESTS

G.R.S. sits on the GSK Vaccines Scientific Advisory Board, consults for AstraZeneca, and is a founding member of RQ Biotechnology. D.I.S. consults for AstraZeneca. Oxford University holds intellectual property related to SARS-CoV-2 mAbs discovered in G.R.S.'s laboratory. S.J.D. is a scientific advisor to the Scottish Parliament on COVID-19.

Received: January 9, 2023
Revised: February 21, 2023
Accepted: March 2, 2023
Published: March 7, 2023

REFERENCES

1. Domingo, E. (2010). Mechanisms of viral emergence. Vet. Res. *41*, 38. https://doi.org/10.1051/vetres/2010010.
2. Sender, R., Bar-On, Y.M., Gleizer, S., Bernshtein, B., Flamholz, A., Phillips, R., and Milo, R. (2021). The total number and mass of SARS-CoV-2 virions. Proc. Natl. Acad. Sci. USA *118*, e2024815118. https://doi.org/10.1073/pnas.2024815118.
3. Lan, J., Ge, J., Yu, J., Shan, S., Zhou, H., Fan, S., Zhang, Q., Shi, X., Wang, Q., Zhang, L., and Wang, X. (2020). Structure of the SARS-CoV-2 spike receptor-binding domain bound to the ACE2 receptor. Nature *581*, 215–220. https://doi.org/10.1038/s41586-020-2180-5.
4. Dejnirattisai, W., Zhou, D., Ginn, H.M., Duyvesteyn, H.M.E., Supasa, P., Case, J.B., Zhao, Y., Walter, T.S., Mentzer, A.J., Liu, C., et al. (2021). The antigenic anatomy of SARS-CoV-2 receptor binding domain. Cell *184*, 2183–2200.e22. https://doi.org/10.1016/j.cell.2021.02.032.
5. Cerutti, G., Guo, Y., Zhou, T., Gorman, J., Lee, M., Rapp, M., Reddem, E.R., Yu, J., Bahna, F., Bimela, J., et al. (2021). Potent SARS-CoV-2 neutralizing antibodies directed against spike N-terminal domain target a single supersite. Cell Host Microbe *29*, 819–833.e7. https://doi.org/10.1016/j.chom.2021.03.005.
6. Barnes, C.O., Jette, C.A., Abernathy, M.E., Dam, K.M.A., Esswein, S.R., Gristick, H.B., Malyutin, A.G., Sharaf, N.G., Huey-Tubman, K.E., Lee, Y.E., et al. (2020). SARS-CoV-2 neutralizing antibody structures inform therapeutic strategies. Nature *588*, 682–687. https://doi.org/10.1038/s41586-020-2852-1.
7. Yuan, M., Liu, H., Wu, N.C., Lee, C.C.D., Zhu, X., Zhao, F., Huang, D., Yu, W., Hua, Y., Tien, H., et al. (2020). Structural basis of a shared antibody response to SARS-CoV-2. Science *369*, 1119–1123. https://doi.org/10.1126/science.abd2321.
8. Gobeil, S.M.C., Janowska, K., McDowell, S., Mansouri, K., Parks, R., Stalls, V., Kopp, M.F., Manne, K., Li, D., Wiehe, K., et al. (2021). Effect of natural mutations of SARS-CoV-2 on spike structure, conformation, and antigenicity. Science *373*, eabi6226. https://doi.org/10.1126/science.abi6226.
9. Dejnirattisai, W., Zhou, D., Supasa, P., Liu, C., Mentzer, A.J., Ginn, H.M., Zhao, Y., Duyvesteyn, H.M.E., Tuekprakhon, A., Nutalai, R., et al. (2021). Antibody evasion by the P.1 strain of SARS-CoV-2. Cell *184*, 2939–2954.e9. https://doi.org/10.1016/j.cell.2021.03.055.
10. Supasa, P., Zhou, D., Dejnirattisai, W., Liu, C., Mentzer, A.J., Ginn, H.M., Zhao, Y., Duyvesteyn, H.M.E., Nutalai, R., Tuekprakhon, A., et al. (2021). Reduced neutralization of SARS-CoV-2 B.1.1.7 variant by convalescent and vaccine sera. Cell *184*, 2201–2211.e7. https://doi.org/10.1016/j.cell.2021.02.033.
11. Zahradník, J., Marciano, S., Shemesh, M., Zoler, E., Harari, D., Chiaravalli, J., Meyer, B., Rudich, Y., Li, C., Marton, I., et al. (2021). SARS-CoV-2 variant prediction and antiviral drug design are enabled by RBD in vitro evolution. Nat. Microbiol. *6*, 1188–1198. https://doi.org/10.1038/s41564-021-00954-4.
12. Dejnirattisai, W., Huo, J., Zhou, D., Zahradník, J., Supasa, P., Liu, C., Duyvesteyn, H.M.E., Ginn, H.M., Mentzer, A.J., Tuekprakhon, A., et al. (2022). SARS-CoV-2 Omicron-B.1.1.529 leads to widespread escape from neutralizing antibody responses. Cell *185*, 467–484.e15. https://doi.org/10.1016/j.cell.2021.12.046.
13. Nutalai, R., Zhou, D., Tuekprakhon, A., Ginn, H.M., Supasa, P., Liu, C., Huo, J., Mentzer, A.J., Duyvesteyn, H.M.E., Dijokaite-Guraliuc, A., et al. (2022). Potent cross-reactive antibodies following Omicron breakthrough in vaccinees. Cell *185*, 2116–2131.e18.
14. Liu, C., Zhou, D., Nutalai, R., Duyvestyn, H., Tuekprakhon, A., Ginn, H., Dejnirattisai, W., Supasa, P., Mentzer, A., Wang, B., et al. (2021). The Beta mAb response underscores the antigenic distance to other SARS-CoV-2 variants Cell. Host and Microbe *30*, 53–68.
15. Pinto, D., Park, Y.J., Beltramello, M., Walls, A.C., Tortorici, M.A., Bianchi, S., Jaconi, S., Culap, K., Zatta, F., De Marco, A., et al. (2020). Cross-neutralization of SARS-CoV-2 by a human monoclonal SARS-CoV antibody. Nature *583*, 290–295. https://doi.org/10.1038/s41586-020-2349-y.
16. Westendorf, K., Žentelis, S., Wang, L., Foster, D., Vaillancourt, P., Wiggin, M., Lovett, E., van der Lee, R., Hendle, J., Pustilnik, A., et al. (2022). LY-CoV1404 (bebtelovimab) potently neutralizes SARS-CoV-2 variants. Cell Rep. *39*, 110812. https://doi.org/10.1016/j.celrep.2022.110812.
17. Chi, X., Yan, R., Zhang, J., Zhang, G., Zhang, Y., Hao, M., Zhang, Z., Fan, P., Dong, Y., Yang, Y., et al. (2020). A neutralizing human antibody binds to the N-terminal domain of the Spike protein of SARS-CoV-2. Science *369*, 650–655. https://doi.org/10.1126/science.abc6952.
18. Liu, Y., Liu, J., Plante, K.S., Plante, J.A., Xie, X., Zhang, X., Ku, Z., An, Z., Scharton, D., Schindewolf, C., et al. (2022). The N501Y spike substitution enhances SARS-CoV-2 infection and transmission. Nature *602*, 294–299. https://doi.org/10.1038/s41586-021-04245-0.
19. van der Straten, K., Guerra, D., van Gils, M.J., Bontjer, I., Caniels, T.G., van Willigen, H.D.G., Wynberg, E., Poniman, M., Burger, J.A., Bouhuijs, J.H., et al. (2022). Antigenic cartography using sera from sequence-confirmed SARS-CoV-2 variants of concern infections reveals antigenic divergence of Omicron. Immunity *55*, 1725–1731.e4. https://doi.org/10.1016/j.immuni.2022.07.018.
20. Cele, S., Jackson, L., Khoury, D.S., Khan, K., Moyo-Gwete, T., Tegally, H., San, J.E., Cromer, D., Scheepers, C., Amoako, D.G., et al. (2022). Omicron extensively but incompletely escapes Pfizer BNT162b2 neutralization. Nature *602*, 654–656. https://doi.org/10.1038/s41586-021-04387-1.
21. McCallum, M., Czudnochowski, N., Rosen, L.E., Zepeda, S.K., Bowen, J.E., Walls, A.C., Hauser, K., Joshi, A., Stewart, C., Dillen, J.R., et al. (2022). Structural basis of SARS-CoV-2 Omicron immune evasion and receptor engagement. Science *375*, 864–868. https://doi.org/10.1126/science.abn8652.

22. Xia, S., Wang, L., Zhu, Y., Lu, L., and Jiang, S. (2022). Origin, virological features, immune evasion and intervention of SARS-CoV-2 Omicron sublineages. Signal Transduct. Target. Ther. *7*, 241. https://doi.org/10.1038/s41392-022-01105-9.
23. Huo, J., Dijokaite-Guraliuc, A., Nutalai, R., Das, R., Zhou, D., Mentzer, A., Fry, E., Mongkolsapaya, J., Ren, J., Stuart, D., and Screaton, G. (2022). Humoral responses against SARS-CoV-2 Omicron BA.2.11, BA.2.12.1 and BA.2.13 from vaccine and BA.1 serum. Cell Discovery *8*, 119. https://doi.org/10.1038/s41421-022-00482-3.
24. Tuekprakhon, A., Nutalai, R., Dijokaite-Guraliuc, A., Zhou, D., Ginn, H.M., Selvaraj, M., Liu, C., Mentzer, A.J., Supasa, P., Duyvesteyn, H.M.E., et al. (2022). Antibody escape of SARS-CoV-2 Omicron BA.4 and BA.5 from vaccine and BA.1 serum. Cell *185*, 2422–2433.e13. https://doi.org/10.1016/j.cell.2022.06.005.
25. Starr, T.N., Greaney, A.J., Hilton, S.K., Ellis, D., Crawford, K.H.D., Dingens, A.S., Navarro, M.J., Bowen, J.E., Tortorici, M.A., Walls, A.C., et al. (2020). Deep mutational scanning of SARS-CoV-2 receptor binding domain reveals constraints on folding and ACE2 binding. Cell *182*, 1295–1310.e20. https://doi.org/10.1016/j.cell.2020.08.012.
26. Turakhia, Y., Thornlow, B., Hinrichs, A.S., De Maio, N., Gozashti, L., Lanfear, R., Haussler, D., and Corbett-Detig, R. (2021). Ultrafast Sample placement on Existing tRees (UShER) enables real-time phylogenetics for the SARS-CoV-2 pandemic. Nat. Genet. *53*, 809–816. https://doi.org/10.1038/s41588-021-00862-7.
27. Dijokaite-Guraliuc, A., Das, R., Nutalai, R., Zhou, D., Mentzer, A.J., Liu, C., Supasa, P., Dunachie, S.J., Lambe, T., Fry, E.E., et al. (2022). Antigenic characterization of SARS-CoV-2 Omicron subvariant BA.4.6. Cell Discov. *8*, 127. https://doi.org/10.1038/s41421-022-00493-0.
28. Huo, J., Dijokaite-Guraliuc, A., Liu, C., Zhou, D., Ginn, H.M., Das, R., Supasa, P., Selvaraj, M., Nutalai, R., Tuekprakhon, A., et al. (2023). A delicate balance between antibody evasion and ACE2 affinity for Omicron BA.2.75. Cell Rep. *42*, 111903. https://doi.org/10.1016/j.celrep.2022.111903.
29. Zhou, D., Duyvesteyn, H.M.E., Chen, C.P., Huang, C.G., Chen, T.H., Shih, S.R., Lin, Y.C., Cheng, C.Y., Cheng, S.H., Huang, Y.C., et al. (2020). Structural basis for the neutralization of SARS-CoV-2 by an antibody from a convalescent patient. Nat. Struct. Mol. Biol. *27*, 950–958. https://doi.org/10.1038/s41594-020-0480-y.
30. Yuan, M., Huang, D., Lee, C.C.D., Wu, N.C., Jackson, A.M., Zhu, X., Liu, H., Peng, L., van Gils, M.J., Sanders, R.W., et al. (2021). Structural and functional ramifications of antigenic drift in recent SARS-CoV-2 variants. Science *373*, 818–823. https://doi.org/10.1126/science.abh1139.
31. Dong, J., Zost, S., Greaney, A., Starr, T.N., Dingens, A.S., Chen, E.C., Chen, R., Case, B., Sutton, R., Gilchuk, P., et al. (2021). Genetic and structural basis for recognition of SARS-CoV-2 spike protein by a two-antibody cocktail. Preprint at bioRxiv. https://doi.org/10.1101/2021.01.27.428529.
32. Weinreich, D.M., Sivapalasingam, S., Norton, T., Ali, S., Gao, H., Bhore, R., Musser, B.J., Soo, Y., Rofail, D., Im, J., et al. (2021). REGN-COV2, a neutralizing antibody cocktail, in outpatients with covid-19. N. Engl. J. Med. *384*, 238–251. https://doi.org/10.1056/NEJMoa2035002.
33. Yuan, M., Zhu, X., He, W.T., Zhou, P., Kaku, C.I., Capozzola, T., Zhu, C.Y., Yu, X., Liu, H., Yu, W., et al. (2022). A broad and potent neutralization epitope in SARS-related coronaviruses. Proc. Natl. Acad. Sci. USA *119*, e2205784119. https://doi.org/10.1073/pnas.2205784119.
34. Sun, Y., and Ho, M. (2020). Emerging antibody-based therapeutics against SARS-CoV-2 during the global pandemic. Antib. Ther. *3*, 246–256. https://doi.org/10.1093/abt/tbaa025.
35. Polack, F.P., Thomas, S.J., Kitchin, N., Absalon, J., Gurtman, A., Lockhart, S., Perez, J.L., Pérez Marc, G., Moreira, E.D., Zerbini, C., et al. (2020). Safety and efficacy of the BNT162b2 mRNA covid-19 vaccine. N. Engl. J. Med. *383*, 2603–2615. https://doi.org/10.1056/NEJMoa2034577.
36. Zhou, D., Dejnirattisai, W., Supasa, P., Liu, C., Mentzer, A.J., Ginn, H.M., Zhao, Y., Duyvesteyn, H.M.E., Tuekprakhon, A., Nutalai, R., et al. (2021). Evidence of escape of SARS-CoV-2 variant B.1.351 from natural and vaccine-induced sera. Cell *184*, 2348–2361.e6. https://doi.org/10.1016/j.cell.2021.02.037.
37. Liu, C., Ginn, H.M., Dejnirattisai, W., Supasa, P., Wang, B., Tuekprakhon, A., Nutalai, R., Zhou, D., Mentzer, A.J., Zhao, Y., et al. (2021). Reduced neutralization of SARS-CoV-2 B.1.617 by vaccine and convalescent serum. Cell *184*, 4220–4236.e13. https://doi.org/10.1016/j.cell.2021.06.020.
38. Eguia, R.T., Crawford, K.H.D., Stevens-Ayers, T., Kelnhofer-Millevolte, L., Greninger, A.L., Englund, J.A., Boeckh, M.J., and Bloom, J.D. (2021). A human coronavirus evolves antigenically to escape antibody immunity. PLoS Pathog. *17*, e1009453. https://doi.org/10.1371/journal.ppat.1009453.
39. Simon-Loriere, E., and Schwartz, O. (2022). Towards SARS-CoV-2 serotypes? Nat. Rev. Microbiol. *20*, 187–188. https://doi.org/10.1038/s41579-022-00708-x.
40. Wang, Q., Ye, S.B., Zhou, Z.J., Li, J.Y., Lv, J.Z., Hu, B., Yuan, S., Qiu, Y., and Ge, X.Y. (2023). Key mutations on spike protein altering ACE2 receptor utilization and potentially expanding host range of emerging SARS-CoV-2 variants. J. Med. Virol. *95*, e28116. https://doi.org/10.1002/jmv.28116.
41. Thakur, N., Gallo, G., Newman, J., Peacock, T.P., Biasetti, L., Hall, C.N., Wright, E., Barclay, W., and Bailey, D. (2022). SARS-CoV-2 variants of concern alpha, beta, gamma and delta have extended ACE2 receptor host ranges. J. Gen. Virol. *103*. https://doi.org/10.1099/jgv.0.001735.
42. He, P., Liu, B., Gao, X., Yan, Q., Pei, R., Sun, J., Chen, Q., Hou, R., Li, Z., Zhang, Y., et al. (2022). SARS-CoV-2 Delta and Omicron variants evade population antibody response by mutations in a single spike epitope. Nat. Microbiol. *7*, 1635–1649. https://doi.org/10.1038/s41564-022-01235-4.
43. Aricescu A.R., Lu W., Jones E.Y. (2006). A time- and cost-efficient system for high-level protein production in mammalian cells. Acta Crystallogr D Biol Crystallogr. *62*, 1243–50. https://doi.org/10.1107/S0907444906029799.
44. Stewart, S.A., Dykxhoorn, D.M., Palliser, D., Mizuno, H., Yu, E.Y., An, D.S., Sabatini, D.M., Chen, I.S., Hahn, W.C., Sharp, P.A., Weinberg, R.A., and Novina, C.D. (2003). Lentivirus-delivered stable gene silencing by RNAi in primary cells. RNA *9*, 493–501. https://doi.org/10.1261/rna.2192803.
45. Nettleship, Joanne, Ren, Jingshan, Rahman, Nahid, Berrow, Nick, Hatherley, Deborah, Barclay, A., and Owens. (2008). Raymond.A pipeline for the production of antibody fragments for structural studies using transient expression in HEK 293T cells. Protein expression and purification *62*, 83–89. https://doi.org/10.1016/j.pep.2008.06.017.
46. Emsley, P., Lohkamp, B., Scott, W.G., and Cowtan, K. (2010). Features and development of coot. Acta Crystallogr. D Biol. Crystallogr. *66*, 486–501.
47. Winter, G., Waterman, D.G., Parkhurst, J.M., Brewster, A.S., Gildea, R.J., Gerstel, M., Fuentes-Montero, L., Vollmar, M., Michels-Clark, T., Young, I.D., et al. (2018). DIALS: implementation and evaluation of a new integration package. Acta Crystallogr. D Struct. Biol. *74*, 85–97. https://doi.org/10.1107/S2059798317017235.
48. Liebschner, D., Afonine, P.V., Baker, M.L., Bunkóczi, G., Chen, V.B., Croll, T.I., Hintze, B., Hung, L.W., Jain, S., McCoy, A.J., et al. (2019). Macromolecular structure determination using X-rays, neutrons and electrons: recent developments in Phenix. Acta Crystallogr. D Struct. Biol. *75*, 861–877. https://doi.org/10.1107/S2059798319011471.
49. Gibson, D.G. (2011). Enzymatic assembly of overlapping DNA fragments. Methods Enzymol. *498*, 349–361. https://doi.org/10.1016/B978-0-12-385120-8.00015-2.
50. Nie, J., Li, Q., Wu, J., Zhao, C., Hao, H., Liu, H., Zhang, L., Nie, L., Qin, H., Wang, M., et al. (2020). Establishment and validation of a pseudovirus neutralization assay for SARS-CoV-2. Emerg. Microbes Infect. *9*, 680–686. https://doi.org/10.1080/22221751.2020.1743767.
51. Ginn, H.M. (2020). Pre-clustering data sets using cluster4x improves the signal-to-noise ratio of high-throughput crystallography drug-screening analysis. Acta Crystallogr. D Struct. Biol. *76*, 1134–1144. https://doi.org/10.1107/S2059798320012619.
52. Walter, T.S., Diprose, J., Brown, J., Pickford, M., Owens, R.J., Stuart, D.I., and Harlos, K. (2003). A procedure for setting up high-throughput nanolitre crystallization experiments. I. Protocol design and validation. J. Appl. Crystallogr. *36*, 308–314. https://doi.org/10.1107/S0021889803001997.

53. Winter, G. (2010). xia2: an expert system for macromolecular crystallography data reduction. J. Appl. Crystallogr. *43*, 186–190.

54. McCoy, A.J., Grosse-Kunstleve, R.W., Adams, P.D., Winn, M.D., Storoni, L.C., and Read, R.J. (2007). Phaser crystallographic software. J. Appl. Crystallogr. *40*, 658–674. https://doi.org/10.1107/S0021889807021206.

55. Stuart, D.I., Levine, M., Muirhead, H., and Stammers, D.K. (1979). Crystal structure of cat muscle pyruvate kinase at a resolution of 2.6 A. J. Mol. Biol. *134*, 109–142. https://doi.org/10.1016/0022-2836(79)90416-9.

56. Punjani, A., Rubinstein, J.L., Fleet, D.J., and Brubaker, M.A. (2017). cryoSPARC: algorithms for rapid unsupervised cryo-EM structure determination. Nat. Methods *14*, 290–296. https://doi.org/10.1038/nmeth.4169.

CellPress
OPEN ACCESS

STAR★METHODS

KEY RESOURCES TABLE

REAGENT or RESOURCE	SOURCE	IDENTIFIER
**Antibodies**		
Fab	Dejnirattisai et al.[4]	N/A
IgG	Dejnirattisai et al.[4] and Liu et al.[4,14]	N/A
Human anti-NP (mAb 206)	Dejnirattisai et al.[4]	N/A
Regeneron mAbs	AstraZeneca	Cat#REGN10933 and REGN10987
AstraZeneca mAbs	AstraZeneca	Cat#AZD1061, AZD8895 and AZD7442
Vir mAbs	Adagio	Cat#S309
Lilly mAbs	Adagio	Cat#Ly-CoV555, Ly-CoV16 and Ly-CoV1404
Adagio mAbs	Adagio	Cat#ADG10, ADG20 and ADG30
Omicron antibodies	Nutalai et al.[13]	N/A
**Bacterial, virus strains, and yeast**		
DH5α bacteria	InVitrogen	Cat#18263012
**Biological samples**		
Serum from Pfizer-vaccinated individuals	University of Oxford	N/A
Serum from AstraZeneca-Oxford-vaccinated individuals	University of Oxford	N/A
Plasma from SARS-CoV-2 patients	John Radcliffe Hospital in Oxford UK, South Africa, and FIOCRUZ (WHO) Brazil	N/A
**Chemicals, peptides, and recombinant proteins**		
His-tagged SARS-CoV-2 RBD	Dejnirattisai et al.[4]	N/A
His-tagged Avi-tagged SARS-CoV-2/BA.2.75 RBD	This paper	N/A
His-tagged SARS-CoV-2/BA.2 + R493Q RBD	This paper	N/A
His-tagged SARS-CoV-2/BA.2 RBD	Nutalai et al.[13]	N/A
His-tagged SARS-CoV-2/BA.4/5 RBD	Tuekprakhon et al.[24]	N/A
His-tagged SARS-CoV-2/Alpha RBD	Supasa et al.[10]	N/A
Human ACE2-hIgG1Fc	Liu et al.[14]	N/A
Phosphate buffered saline tablets	Sigma-Aldrich	Cat#P4417
Dulbecco's Modified Eagle Medium, high glucose	Sigma-Aldrich	Cat#D5796
Dulbecco's Modified Eagle Medium, low glucose	Sigma-Aldrich	Cat#D6046
FreeStyle™ 293 Expression Medium	Gibco	Cat#12338018
L-Glutamine–Penicillin–Streptomycin solution	Sigma-Aldrich	Cat#G1146
GlutaMAX™ Supplement	Gibco	Cat#35050061
UltraDOMA PF Protein-free Medium	Lonza	Cat#12–727F
Opti-MEM™	Gibco	Cat#11058021
Fetal Bovine Serum	Gibco	Cat#12676029
Strep-Tactin®XT	IBA Lifesciences	Cat#2-1206-025
HEPES	Melford	Cat#34587–39108
LB broth	Fisher Scientific UK	Cat#51577–51656
Trypsin-EDTA	Gibco	Cat#2259288

(Continued on next page)

Continued

REAGENT or RESOURCE	SOURCE	IDENTIFIER
TrypLE™ Express Enzyme	Gibco	Cat#12604013
L-Glutamine 200 mM (100X)	Gibco	Cat#2036885
Isopropyl β-d-1-thiogalactopyranoside	Meridian Bioscience	Cat#BIO-37036
Kanamycin	Melford	Cat#K22000
Ampicillin	Sigma-Aldrich	Cat#PHR2838
Agarose	Sigma-Aldrich	Cat#A2929
SYBR™ Safe DNA Gel Stain	Fisher Scientific UK	Cat#S33102
QIAprep Spin Miniprep Kit	Qiagen	Cat#27106X4
QIAquick® PCR & Gel Cleanup Kit	Qiagen	Cat#28704
Phusion™ High-Fidelity DNA Polymerase	Fisher Scientific UK	Cat#F530S
Bright-Glo™ Luciferase Assay System	Promega	Cat#E2620
HIV1 p24 ELISA Kit	Abcam	Cat#ab218268
NaCl	Sigma-Aldrich	Cat#S9888
Sensor Chip Protein A	Cytiva	Cat#29127555
Biotin CAPture Kit, Series S	Cytiva	CAT#28920234
HBS-EP+ Buffer 10×	Cytiva	Cat# BR100669
Regeneration Solution (glycine-HCl pH 1.7)	Cytiva	Cat# BR100838
**Deposited data**		
Crystal structures of: SARS-CoV-2 Delta-RBD/BA.2–36 and SARS-CoV-2 Delta-RBD/EY6A/BA.2–10 and SARS-CoV-2 Delta RBD/BA.2–13/C1	This paper	PDB: 8BBO, PDB:8BBN, PDB:8C3V
CryoEM structure of: SARS-CoV-2 Delta-RBD/BA.2–23/BA.2–36/EY6A/Fab-45	This paper	PDB:8BCZ, EMDB:EMD-15971
**Experimental models: Cell lines**		
HEK293 cells	ATCC	Cat#CRL-3216
Expi293F™ Cells	Gibco,	Cat#A14527
HEK293T/17 cells	ATCC	Cat#CRL-11268™
HEK293T cells	ATCC	Cat#CRL-11268
Vero CCL-81 cells	ATCC	Cat#CCL-81
VeroE6/TMPRSS2 cells	NIBSC	Ref. no. 100978
**Recombinant DNA**		
Vector: pHLsec	Aricescu et al.[43]	N/A
Vector: pNEO	Aricescu et al.[43]	N/A
Vector: pHLsec-SARS-CoV-2 spike of Omicron	Nutalai et al.[13]	N/A
Vector: pOPINTTGneo-BAP-SARS-CoV-2 RBD of BA.2.75	This paper	N/A
Vector: pNEO-SARS-CoV-2 RBD of BA.2	Nutalai et al.[13]	N/A
Vector: pNEO-SARS-CoV-2 RBD of BA.4/5	Tuekprakhon et al.[24]	N/A
Vector: pNEO-SARS-CoV-2 RBD of BA.2 + R493Q	This paper	N/A
Vector: pNEO-SARS-CoV-2 RBD of Alpha	Supasa et al.[10]	N/A
Vector: pCMV-VSV-G	Stewart et al.[44]	Addgene plasmid # 8454
pHR-SIN-ACE2	Alain Townsend, Oxford	N/A
Vector: pOPING-ET	Nettleship et al.[45]	N/A
Vector: pcDNA-SARS-CoV-2 spike of Victoria strain (S247R)	Liu et al.[14]	N/A

(*Continued on next page*)

Continued

REAGENT or RESOURCE	SOURCE	IDENTIFIER
Vector: pcDNA-SARS-CoV-2 spike of BA.1 strain (A67V, Δ69–70, T95I, G142D/Δ143-145, Δ211/L212I, ins214EPE, G339D, S371L, S373P, S375F, K417N, N440K, G446S, S477N, T478K, E484A, Q493R, G496S, Q498R, N501Y, Y505H, T547K, D614G, H655Y, N679K, P681H, N764K, D796Y, N856K, Q954H, N969K, L981F)	Nutalai et al.[13]	N/A
Vector: pcDNA-SARS-CoV-2 spike of BA.1.1 strain (A67V, Δ69–70, T95I, G142D/Δ143-145, Δ211/L212I, ins214EPE, G339D, R346K, S371L, S373P, S375F, K417N, N440K, G446S, S477N, T478K, E484A, Q493R, G496S, Q498R, N501Y, Y505H, T547K, D614G, H655Y, N679K, P681H, N764K, D796Y, N856K, Q954H, N969K, L981F)	Nutalai et al.[13]	N/A
Vector: pcDNA-SARS-CoV-2 spike of BA.2 strain (T19I, Δ24–26, A27S, G142D, V213G, G339D, S371F, S373P, S375F, T376A, D405N, R408S, K417N, N440K, S477N, T478K, E484A, Q493R, Q498R, N501Y, Y505H, D614G, H655Y, N679K, P681H, N764K, D796Y, Q954H, N969K)	Nutalai et al.[13]	N/A
Vector: pcDNA-SARS-CoV-2 spike of BA.2.12.1 strain (T19I, Δ24–26, A27S, G142D, V213G, G339D, S371F, S373P, S375F, T376A, D405N, R408S, K417N, N440K, L452Q, S477N, T478K, E484A, Q493R, Q498R, N501Y, Y505H, D614G, H655Y, N679K, P681H, S704L, N764K, D796Y, Q954H, N969K)	Nutalai et al.[13]	N/A
Vector: pcDNA-SARS-CoV-2 spike of BA.4/5 strain (T19I, Δ24–26, A27S, Δ69-70, G142D, V213G, G339D, S371F, S373P, S375F, T376A, D405N, R408S, K417N, N440K, L452R, S477N, T478K, E484A, F486V, Q498R, N501Y, Y505H, D614G, H655Y, N679K, P681H, N764K, D796Y, Q954H, N969K)	Tuekprakhon et al.[24]	N/A
Vector: pcDNA-SARS-CoV-2 spike of BA.2.75 strain (T19I, Δ24–26, A27S, G142D, K147E, W152R, F157L, I210V, V213G, G257S, D339H, S371F, S373P, S375F, T376A, D405N, R408S, K417N, N440K, G446S, N460K, S477N, T478K, E484A, R493Q, Q498R, N501Y, Y505H, D614G, H655Y, N679K, P681H, N764K, D796Y, Q954H, N969K)	This paper	N/A
Vector: pcDNA-SARS-CoV-2 spike of BA.2 + D339H strain (T19I, Δ24–26, A27S, G142D, V213G, D339H, S371F, S373P, S375F, T376A, D405N, R408S, K417N, N440K, S477N, T478K, E484A, Q493R, Q498R, N501Y, Y505H, D614G, H655Y, N679K, P681H, N764K, D796Y, Q954H, N969K)	This paper	N/A

(*Continued on next page*)

Continued

REAGENT or RESOURCE	SOURCE	IDENTIFIER
Vector: pcDNA-SARS-CoV-2 spike of BA.2 + R493Q strain (T19I, Δ24–26, A27S, G142D, V213G, G339D, S371F, S373P, S375F, T376A, D405N, R408S, K417N, N440K, S477N, T478K, E484A, R493Q, Q498R, N501Y, Y505H, D614G, H655Y, N679K, P681H, N764K, D796Y, Q954H, N969K)	This paper	N/A
Vector: pcDNA-SARS-CoV-2 spike of BA.2 + G446S strain (T19I, Δ24–26, A27S, G142D, V213G, G339D, S371F, S373P, S375F, T376A, D405N, R408S, K417N, N440K, G446S, S477N, T478K, E484A, Q493R, Q498R, N501Y, Y505H, D614G, H655Y, N679K, P681H, N764K, D796Y, Q954H, N969K)	This paper	N/A
Vector: human IgG1 heavy chain	German Cancer Research Center, Heidelberg, Germany (H. Wardemann	N/A
Vector: human lambda light chain	German Cancer Research Center, Heidelberg, Germany (H. Wardemann	N/A
Vector: human kappa light chain	German Cancer Research Center, Heidelberg, Germany (H. Wardemann	N/A
Vector: Human Fab	University of Oxford	N/A
Vector: pJYDC1	Adgene	ID: 162,458
TM149 BirA pDisplay	University of Oxford, NDM (C. Siebold)	N/A
**Software and algorithms**		
COOT	Emsley et al.[46]	https://www2.mrc-lmb.cam.ac.uk/personal/pemsley/coot/
Xia2-dials	Winter et al.[47]	https://xia2.github.io/parameters.html
PHENIX	Liebschner et al.[48]	https://www.phenix-online.org/
PyMOL	Warren DeLano, Schrodinger	https://pymol.org/
Data Acquisition Software 11.1.0.11	Fortebio	https://www.fortebio.com/products/octet-systems-software
Data Analysis Software HT 11.1.0.25	Fortebio	https://www.fortebio.com/products/octet-systems-software
CryoSPARC v4.1.2	Structura Biotechnology Inc.	https://cryosparc.com/
SerialEM (version 3.8.0 beta)	https://bio3d.colorado.edu/SerialEM/	N/A
EPU	Thermo Fisher	https://www.thermofisher.com/uk/en/home/electron-microscopy/products/software-em-3d-vis/epu-software.html
Prism 9.0	GraphPad	https://www.graphpad.com/scientific-software/prism/
Yeast display titration curve fitting were done by the standard non-cooperative Hill equation, fitted by nonlinear least-squares regression with two additional parameters using Python 3.7	Zahradnik et al.[11]	N/A
IBM SPSS Software 27	IBM	https://www.ibm.com

(*Continued on next page*)

Continued

REAGENT or RESOURCE	SOURCE	IDENTIFIER
Mabscape	This paper	https://github.com/helenginn/mabscape https://snapcraft.io/mabscape
Biacore T200 Evaluation Software 3.1	Cytiva	www.cytivalifesciences.com
Other		
X-ray data were collected at beamline I03, Diamond Light Source, under proposal ib27009 for COVID-19 rapid access	This paper	https://www.diamond.ac.uk/covid-19/for-scientists/rapid-access.html
Cryo-EM data were collected at OPIC, Division of Structural Biology, University of Oxford	This paper	https://www.opic.ox.ac.uk
TALON® Superflow Metal Affinity Resin	Clontech	Cat#635668
HiLoad® 16/600 Superdex® 200 pg	Cytiva	Cat#28-9893-35
Superdex 200 increase 10/300 GL column	Cytiva	Cat#28990944
HisTrap nickel HP 5-mL column	Cytiva	Cat#17524802
HiTrap Heparin HT 5-mL column	Cytiva	Cat#17040703
Amine Reactive Second-Generation (AR2G) Biosensors	Fortebio	Cat#18–5092
Octet RED96e	Fortebio	https://www.fortebio.com/products/label-free-bli-detection/8-channel-octet-systems
Buffer exchange system “QuixStand”	GE Healthcare	Cat#56-4107-78
Cartesian dispensing system	Genomic solutions	Cat#MIC4000
Hydra-96	Robbins Scientific	Cat#Hydra-96
96-well crystallization plate	Greiner bio-one	Cat#E20113NN
Crystallization Imaging System	Formulatrix	Cat#RI-1000
Sonics vibra-cell vcx500 sonicator	VWR	Cat#432–0137
Biacore T200	Cytiva	https://www.cytivalifesciences.com/en/us/shop/protein-analysis/spr-label-free-analysis/systems/biacore-t200-p-05644

RESOURCE AVAILABILITY

Lead contact

Resources, reagents and further information requirement should be forwarded to and will be responded by the lead contact, David I Stuart (dave@strubi.ox.ac.uk).

Materials availability

Reagents generated in this study are available from the lead contact with a completed Materials Transfer Agreement.

Data and code availability

- Coordinates are deposited in the PDB: Delta-RBD/BA.2-36, PDB:8BBO. Delta-RBD/EY6A/BA.2-10, PDB:8BBN. Delta-RBD/BA.2-13/C1, PDB:8C3V. Delta-RBD/BA.2-23/BA.2-36/EY6A/Fab-45, PDB:8BCZ and EMDB:EMD-15971.
- This paper does not report original code.
- Any additional information required to reanalyse the data reported in this paper is available from the lead contact upon request.

EXPERIMENTAL MODEL AND SUBJECT DETAILS

Bacterial strains and cell culture

Vero (ATCC CCL-81) and VeroE6/TMPRSS2 cells were cultured at 37°C in Dulbecco’s Modified Eagle medium (DMEM) high glucose (Sigma-Aldrich) supplemented with 10% fetal bovine serum (FBS), 2 mM GlutaMAX (Gibco, 35050061) and 100 U/ml of penicillin–streptomycin. Human mAbs were expressed in HEK293T cells cultured in FreeStyle 293 Expression Medium (Cat# 12338018, Gibco)

at 37°C with 5% CO_2. HEK293T (ATCC CRL-11268) cells were cultured in DMEM high glucose (Sigma-Aldrich) supplemented with 10% FBS, 1% 100X Mem Neaa (Gibco) and 1% 100X L-Glutamine (Gibco) at 37°C with 5% CO_2. To express RBD, RBD variants and ACE2, HEK293T cells were cultured in DMEM high glucose (Sigma) supplemented with 2% FBS, 1% 100X Mem Neaa and 1% 100X L-Glutamine at 37°C for transfection. BA.2 RBD were expressed in HEK293T (ATCC CRL-11268) cells cultured in FreeStyle 293 Expression Medium (Cat# 12338018, Gibco) at 37°C with 5% CO_2. *E.coli DH5α* bacteria were used for transformation and large-scale preparation of plasmids. A single colony was picked and cultured in LB broth at 37 °C at 200 rpm in a shaker overnight.

Sera from BA.1 infected cases, study subjects

Following informed consent, individuals with omicron BA.1 were co-enrolled into the ISARIC/WHO Clinical Characterisation Protocol for Severe Emerging Infections [Oxford REC C, ref. 13/SC/0149] and the "Innate and adaptive immunity against SARS-CoV-2 in healthcare worker family and household members" protocol affiliated to the Gastro-intestinal illness in Oxford: COVID sub study [Sheffield REC, ref. 16/YH/0247] further approved by the University of Oxford Central University Research Ethics Committee. Diagnosis was confirmed through reporting of symptoms consistent with COVID-19 or a positive contact of a known Omicron case, and a test positive for SARS-CoV-2 using reverse transcriptase polymerase chain reaction (RT-PCR) from an upper respiratory tract (nose/throat) swab tested in accredited laboratories and lineage sequence confirmed through national reference laboratories. A blood sample was taken following consent at least 10 days after PCR test confirmation. Clinical information including severity of disease (mild, severe or critical infection according to recommendations from the World Health Organisation) and times between symptom onset and sampling and age of participant was captured for all individuals at the time of sampling (see Table S4).

Sera and PBMC from BA.2 infected study subjects

Following informed consent, healthcare workers with BA.2 infection were co-enrolled under the Sheffield Biobank study (STHObs) (18/YH/0441) during March 2022 (median 22 March, BA.2 sequence was confirmed for all samples taken before 22 March). All individuals had PCR-confirmed symptomatic disease and sequence confirmed BA.2 infection through national UKHSA sequencing data. A blood sample was taken following consent at least 12 days after PCR test confirmation. Clinical information including vaccination history, times between symptom onset and sampling and age of participant was captured for all individuals at the time of sampling (see Table S4).

Sera from BA.4/5 infected cases, study subjects

Following informed consent, individuals with omicron BA.4 or BA.5 were co-enrolled into one or more of the following three studies: the ISARIC/WHO Clinical Characterisation Protocol for Severe Emerging Infections [Oxford REC C, ref. 13/SC/0149], the "Innate and adaptive immunity against SARS-CoV-2 in healthcare worker family and household members" protocol (approved by the University of Oxford Central University Research Ethics Committee), or the Gastro-intestinal illness in Oxford: COVID sub study [Sheffield REC, ref. 16/YH/0247]. Diagnosis was confirmed through reporting of symptoms consistent with COVID-19, hospital presentation, and a test positive for SARS-CoV-2 using reverse transcriptase polymerase chain reaction (RT-PCR) from an upper respiratory tract (nose/throat) swab tested in accredited laboratories and lineage sequence confirmed through national reference laboratories in the United Kingdom. A blood sample was taken following consent at least 14 days after PCR test confirmation. Clinical information including severity of disease (mild, severe or critical infection according to recommendations from the World Health Organisation) and times between symptom onset and sampling and age of participant was captured for all individuals at the time of sampling (see Table S4).

Sera from Pfizer vaccinees

Pfizer vaccine serum was obtained from volunteers who had received three doses of the BNT162b2 vaccine. Vaccinees were Health Care Workers, based at Oxford University Hospitals NHS Foundation Trust, not known to have prior infection with SARS-CoV-2 and were enrolled in the OPTIC Study as part of the Oxford Translational Gastrointestinal Unit GI Biobank Study 16/YH/0247 [research ethics committee (REC) at Yorkshire & The Humber – Sheffield] which has been amended for this purpose on 8 June 2020. The study was conducted according to the principles of the Declaration of Helsinki (2008) and the International Conference on Harmonization (ICH) Good Clinical Practice (GCP) guidelines. Written informed consent was obtained for all participants enrolled in the study. Participants were sampled approximately 28 days (range 25–56) after receiving a third "booster dose of BNT162B2 vaccine. The mean age of vaccinees was 37 years (range 22–66), 21 male and 35 female.

METHOD DETAILS

Isolation of BA.2 S-specific single B cells by FACS

BA.2 S-specific single B cell sorting was performed as previously described.[4] Briefly, PBMC were stained with LIVE/DEAD Fixable Aqua dye (Invitrogen) followed by recombinant trimeric S-twin-Strep of BA.2. Cells were then incubated with CD3-FITC, CD14-FITC, CD16-FITC, CD56-FITC, IgM-FITC, IgA-FITC, IgD-FITC, IgG-BV786 and CD19-BUV395, along with Strep-MAB-DY549 to stain the twin strep tag of the S protein. IgG+ memory B cells were gated as CD19+, IgG+, CD3-, CD14-, CD56-, CD16-, IgM-, IgA- and IgD-, and S+ was further selected and single cells were sorted into 96-well PCR plates with 10 μL of catching buffer (Tris, Nuclease free-H2O and RNase inhibitor). Plates were briefly centrifuged at 2000xg for 1 min and left on dry ice before being stored at −80°C.

Cloning and expression of BA.2 S-specific human mAbs

BA.2 S-specific human mAbs were cloned and expressed as described previously.[4] Briefly, genes for Ig IGHV, Ig Vκ and Ig Vλ were recovered from positive wells by RT-PCR. Genes encoding Ig IGHV, Ig Vκ and Ig Vλ were then amplified using Nested-PCR by a cocktail of primers specific to human IgG. PCR products of HC and LCs were ligated into the expression vectors of human IgG1 or immunoglobulin κ-chain or λ-chain by Gibson assembly.[49] For mAb expression, plasmids encoding HCs and LCs were co-transfected by PEI-transfection into a HEK293T cell line, and supernatants containing mAbs were collected and filtered 4–5 days after transfection, and the supernatants were further characterized or purified.

ACE2 binding inhibition assay by ELISA

MAXISORP immunoplates were coated with 5 μg/mL of purified ACE2-His protein overnight at 4°C and then blocked by 2% BSA in PBS. Meanwhile, mAbs were serially diluted and mixed with 2.5 μg/mL of recombinant BA.1 trimeric S-twin-Strep. Antibody-S protein mixtures were incubated at 37°C for 1 h. After incubation, the mixtures were transferred into the ACE2-coated plates and incubated for 1 h at 37°C. After wash, StrepMAB-Classic (2-1507-001, iba) was diluted at 0.2 μg/mL by 2% BSA and used as primary antibody followed by Goat anti-mouse IgG-AP (#A16093, Invitrogen) at 1:2000 dilution. The reaction was developed by adding PNPP substrate and stopped with NaOH. The absorbance was measured at 405nm. The ACE2/S binding inhibition was calculated by comparing to the antibody-free control well. IC50 was determined using the Probit program from the SPSS package.

Pseudovirus plasmid construction and lentiviral particle production

Pseudotyped lentiviruses expressing SARS-CoV-2 S proteins from ancestral strain (Victoria, S247R), BA.2 and BA.4/5 were constructed as described previously.[13,24,37,50] We applied the same method to construct BA.2.75, BA.2.75.2, BA.2.3.20, BA.2.10.4, BJ.1, BS.1, BN.1, XBB, and XBB.1 by adding more mutations into the BA.2 construct. To generate BA.2.75, we added K147E, W152R, F157L, I210V, G275S, G446S and N460K into BA.2 backbone, also changed 339D in BA.2 S into 339H, and reversed 493R in BA.2 to 493Q as in the ancestral strain. To create BA.2.75.2, we added R346T, F486S and D1199N into BA.2.75 backbone. To create BA.2.3.20, we introduced M153T, N164K, H245N, G257D, K444R, N450D, L452M, and N460K, as well we changed L452R in BA.2 into L452M, and reversed 493R in BA.2 to 493Q as in the ancestral strain. BA.2.10.4 was generated by introducing W64R, G446S, F486P, R493Q, S494P and P1143L into BA.2 backbone, as well as deleting aa142-144 and 243–244. To generate BJ.1, we introduced V83A, deletion at position 145, H146Q, Q183E, R346T, L368I, V445P, G446S, V483A, F490V and S1003I, in addition we changed 213G in BA.2 into 213E and also changed 339D in BA.2 S into 339H in BA.2 backbone. To create BS.1, we added aa144 deletion, G257V, R346T, L452R, G476S, N460K, F486S, F490S, and Q493H reversion into BA.2 backbone. To generate BN.1, the BA.2.75 vector was used as backbone with addition of R346T, K357T and F490S. To construct XBB, we added V83A, aa144 deletion, H146Q, Q183E, V213E, R346T, L368I, V445P, G446S, N460K, F486S, and F490S into BA.2 backbone and also changed 339D in BA.2 to 339H and reversed 493Q to H. XBB.1 was constructed by adding G252V into XBB. The same method was used to construct BF.7, BA4.6, BQ.1 and BQ.1.1 with new RBD mutations from BA.2 lineages. To create BF.7, R346T was added into BA.4 backbone. To generate BA.4.6, we introduced R346T and N658S into BA.4 backbone. BQ.1 was constructed by introducing K444T and N460K into BA.4 backbone, while BQ.1.1 was to add R346T into BQ.1 construct. The resulting pcDNA3.1 plasmid carrying S gene was used for generating pseudoviral particles together with the lentiviral packaging vector and transfer vector encoding luciferase reporter. All the constructs were sequence confirmed.

Pseudoviral neutralization test

The pseudoviral neutralization test has been described previously.[37] Briefly, the neutralizing activity of potent monoclonal antibodies generated from donors who had recovered from BA.1 and BA.2 infections were tested against Victoria, BA.2, BA.2.75, BA.2.75.2, BA.2.3.20, BJ.1, BA.4/5, BA.4.6, BA.4.7 and chimeric BA.4. Four-fold serial diluted mAbs were incubated with pseudoviral particles at 37°C, 5% CO_2 for 1 h. Stable HEK293T/17 cells expressing human ACE2 were then added to the mixture at 1.5×10^4 cells/well. 48 h post infection, culture supernatants were removed and 50 μL of 1:2 Bright-Glo TM Luciferase assay system (Promega, USA) in 1 × PBS was added to each well. The reaction was incubated at room temperature for 5 min and firefly luciferase activity was measured using CLARIOstar (BMG Labtech, Ortenberg, Germany). The percentage neutralization was calculated relative to the control. Probit analysis was used to estimate the dilution that inhibited half maximum pseudotyped lentivirus infection (PVNT50). To determine the neutralizing activity of convalescent plasma/serum samples or vaccine sera, 3-fold serial dilutions of samples were incubated with pseudoviral particles for 1 h and the same strategy as for mAbs was applied.

RBD production for structural analysis

Delta RBD was cloned into pNEO vector as described previously.[37] Protein production was as described by Zhou et al.[36] Briefly, plasmids encoding RBD were transiently expressed in HEK293T (ATCC CRL-11268) cells. The conditioned medium was dialyzed and purified with a 5-mL HisTrap nickel column (GE Healthcare) and further polished using a Superdex 75 HiLoad 16/60 gel filtration column (GE Healthcare).

IgG mAbs and Fabs production

AstraZeneca and Regeneron antibodies were provided by AstraZeneca, Vir, Lilly and Adagio antibodies were provided by Adagio, LY-CoV1404 was provided by LifeArc. For the in-house antibodies, heavy and light chains of the indicated antibodies were transiently transfected into 293T cells and antibody purified from supernatant on protein A as previously described.[13] Fabs were digested from purified IgGs with papain using a Pierce Fab Preparation Kit (Thermo Fisher), following the manufacturer's protocol.

Competition assays of anti-Omicron BA.2 RBD mAbs

Competition assays of anti-Omicron BA.2 RBD mAbs were performed on an Octet Red 96e machine (Sartorius) using Octet Anti-HIS (HIS2) Biosensors (Sartorius). His-tagged Omicron BA.2 RBD dissolved in the running buffer (10 mM HEPES, pH 7.4 and 150 mM NaCl) was used as the ligand and was first immobilized onto the biosensors. The biosensors were then washed with the running buffer to remove unbound RBD. Each biosensor was dipped into different saturating mAbs (Ab1) to saturate the bound RBD, except one biosensor was dipped into running buffer in this step, acting as the reference. Then all biosensors were washed with the running buffer again and dipped into wells containing the same competing antibody (Ab2). The y axis values of signals of different saturating antibodies in this step were divided by the value of the reference channel to get ratio results of different Ab1-Ab2 pairs. Ratio results close to 0 indicated total competition while 1 indicated no competition.

Antibody mapping to RBD surface

All BA.2 antibodies and several antibodies with previously solved structures (mAb-45, -58, −278, EY6A AZD8895, AZD1061)[4] were used in a competition assay prepared for antibody mapping to the RBD surface. Antibody mapping was carried out using *Mabscape*[4] and cluster4x.[51] Mid-point positions of mAb-45, -58, −278, EY6A AZD8895, AZD1061[4] were calculated from crystal structures and used to seed the analysis in 1000 Monte Carlo runs, whereas several known structural positions were not included in the analysis and used as a cross-check. A total of 178 Monte Carlo runs formed a single cluster with the lowest score and these were used to calculate average positions for BA.2 antibodies.

Crystallization, X-ray data collection and structure determination

Purified Delta RBD was deglycosylated with Endo F1 and combined with Fabs EY6A and BA2-10 in a 1:1:1 M ratio with a final concentration of 7 mg mL^{-1} and combined with Fab BA2-36 in a 1:1 M ratio with a final concentration of 13 mg mL^{-1}. The samples were incubated at room temperature for 30 min. Initial screening of crystals was set up in Crystalquick 96-well X plates (Greiner Bio-One) with a Cartesian Robot using the nanoliter sitting-drop vapor-diffusion method, with 100 nL of protein plus 100 nL of reservoir in each drop, as previously described.[52] Good crystals of Delta RBD/EY6A/BA2-10 complex were formed in Hampton Research PEGRx condition 2–18, containing 10% (v/v) 2-propanol, 0.1 M BICINE, pH 8.5 and 30% (w/v) PEG 1500. Crystals of Delta RBD/BA.2–13/C1 nanobody complex were grown in the condition containing 0.1 M BICINE pH 8.5 and 15% (w/v) PEG 1,500. Good crystals of the Delta RBD/BA2-36 complex were formed in Hampton Research PEGRx condition 2–31, containing 2% (v/v) PEG400, 0.1M imidazole pH7.0 and 24% (w/v) PEG MME 5000.

Crystals were mounted in loops and dipped in solution containing 25% glycerol and 75% mother liquor for a second before being frozen in liquid nitrogen. Diffraction data of Delta RBD/EY6A/BA2-10 and Delta RBD/BA2-36 were collected at 100 K at beamline I03 of Diamond Light Source, UK, using the automated queue system that allows unattended automated data collection (https://www.diamond.ac.uk/Instruments/Mx/I03/I03-Manual/Unattended-Data-Collections.html). 3600 diffraction images of 0.1° each were collected from a single crystal of the Delta-RBD/BA.2–10/EY6A complex. 7200 diffraction images were collected from two crystals for Delta-RBD/BA.2–36 complex. 360° of diffraction data for Delta RBD/BA.2–13/C1 were collected at beamline I04. Data were automatically processed with Xia2-dials.[53,47] Each of the structures was determined using molecular replacement with Phaser[54] and a model of our previously determined RBD/Fab structures that has maximum sequence identity with the current structure.[4,13,14] Model rebuilding used COOT[46] and refinement Phenix.[48]

Data collection and structure refinement statistics are given in Table S3. Structural comparisons used SHP[55] and figures were prepared with PyMOL (The PyMOL Molecular Graphics System, Version 1.2r3pre, Schrödinger, LLC).

Cryo-EM structure determination

For Delta-RBD/BA.2–23/BA.2–36/EY6A/mAb-4, delta RBD was incubated with a 1.1 M excess of each Fab on ice for ca. 10 min before application of a 3 μL aliquot of this complex mixture to a freshly glow-discharged (35 s, high with a Plasma Cleaner PDC-002-CE, Harrick Plasma) Quantifoil 2/1 300 mesh grids. Excess liquid was removed by blotting for 6 s with a force of −1 using vitrobot filter paper (grade 595, Ted Pella Inc.) at 4.5°C, 100% reported humidity before plunge freezing into liquid ethane using a Vitrobot Mark IV (Thermo Fisher).

Movies, 20,535 in total, were collected on a Titan Krios operating at 300 kV equipped with a Falcon-IV Selectris at 130 kX magnification, corresponding to a calibrated pixel size of 0.7303 $Å^2$ with a total dose of 50 e−/$Å^2$ using EPU software (ThermoFisher scientific) and defocus range of 0.8–2.6 μm in EER format.

Data were pre-processed on-the-fly in the cryoSPARC live interface, using initial 2D classes from blob-picked particles a template for template picking.[56] 3,529,798, picked particles, were then 2D classified into 250 classes in cryoSPARC v3.3.2 'static' version, and 335,851 particles that were not obviously 'junk' were further classified, resulting in 29,589 particles in classes representing a variety of

views. These were then used as input for ab-initio 3D reconstruction to three classes before heterogeneous refinement using the resulting volume set. One class, containing 167,492 particles, clearly commensurate with an RBD decorated with four Fabs, was then refined using non-uniform refinement before unbinning and further refinement to a final reported resolution of 2.9 Å resolution (−93 reported global b-factor).

Phylogenetic tree

The phylogenetic tree was generated by pruning the Nextclade reference tree (https://nextstrain.org/nextclade/sars-cov-2/21L) which contains one sequence per Pango lineage. The tree was generated with a Snakemake workflow using the Augur toolchain. The workflow is available at: https://github.com/neherlab/nextclade_data_workflows/tree/27bf7e0b4f62cbbbc9a8ac96db1587cd76b3ae10.

The topology of the tree was constrained using the Usher tree that incorporates nearly all sequences available through GISAID.[26] The tree was pruned using a BioPython script and visualized using Figtree.

QUANTIFICATION AND STATISTICAL ANALYSIS

Statistical analyses are reported in the results and figure legends. Neutralization was measured on pseudovirus. The percentage reduction was calculated and IC_{50} determined using the probit program from the SPSS package. The Wilcoxon matched-pairs signed rank test was used for the analysis and two-tailed p values were calculated on geometric mean values.

Cell Reports, Volume *42*

Supplemental information

Rapid escape of new SARS-CoV-2 Omicron variants from BA.2-directed antibody responses

Aiste Dijokaite-Guraliuc, Raksha Das, Daming Zhou, Helen M. Ginn, Chang Liu, Helen M.E. Duyvesteyn, Jiandong Huo, Rungtiwa Nutalai, Piyada Supasa, Muneeswaran Selvaraj, Thushan I. de Silva, Megan Plowright, Thomas A.H. Newman, Hailey Hornsby, Alexander J. Mentzer, Donal Skelly, Thomas G. Ritter, Nigel Temperton, Paul Klenerman, Eleanor Barnes, Susanna J. Dunachie, OPTIC consortium, Cornelius Roemer, Thomas P. Peacock, Neil G. Paterson, Mark A. Williams, David R. Hall, Elizabeth E. Fry, Juthathip Mongkolsapaya, Jingshan Ren, David I. Stuart, and Gavin R. Screaton

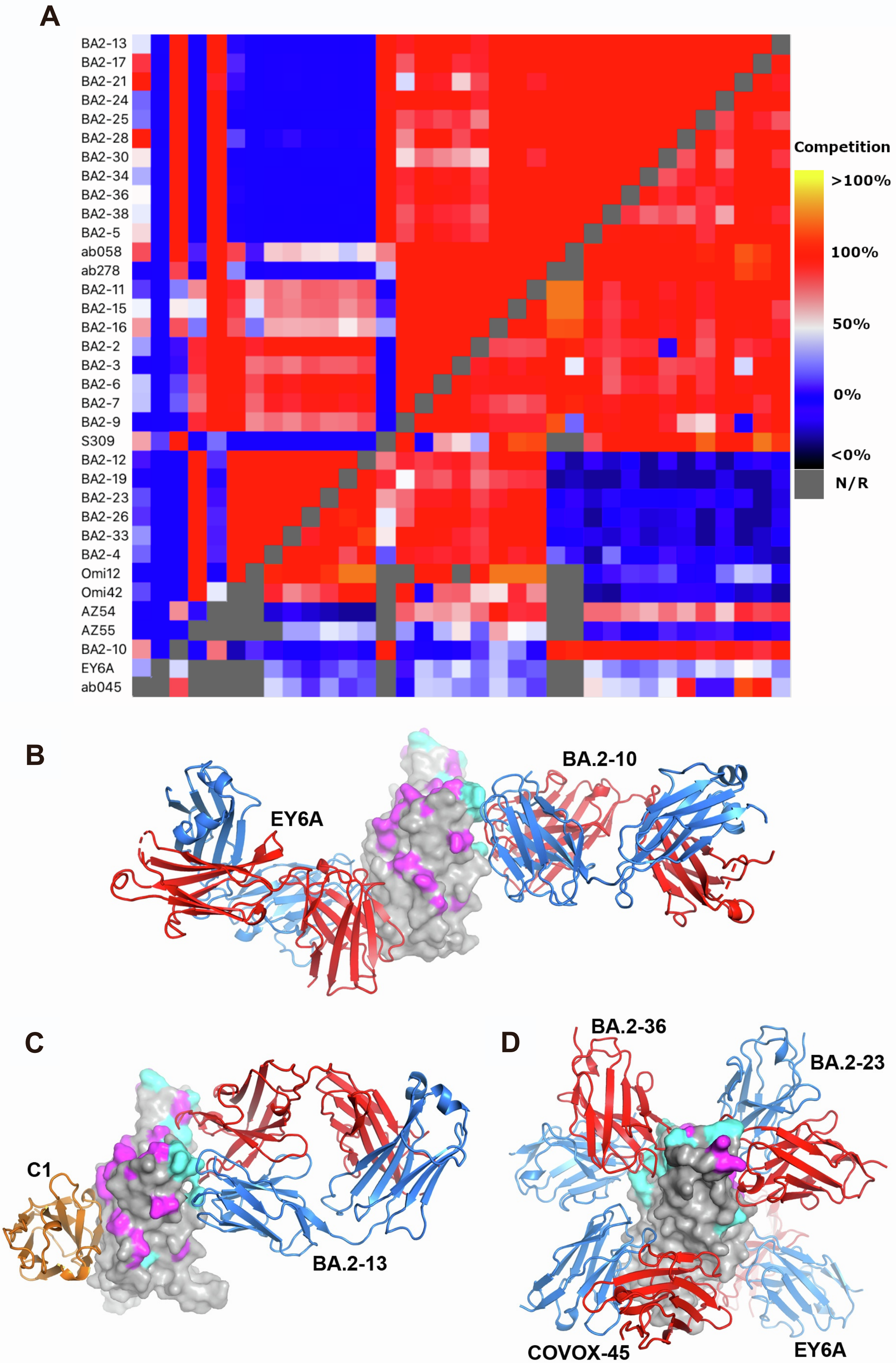

Figure S1. Mapping of all antibodies and ternary and quintuple complex structures. (A) Competition mapping, as measured by BLI (bottom right) and as calculated by *Mabscape* (top left). (B) Delta-RBD with BA.2-10 and EY6A Fabs. (C) Delta-RBD with BA.2-13 Fab and C1 nanobody. (D) Delta-RBD with BA.2-23, BA.2-36, EY6A and COVOX-45 Fabs. RBD is drawn as grey surface representation with BA.4 mutation sites highlighted in magenta and the additional mutation sites of all variants shown in Figure 1A are shown in cyan. Fabs are shown as ribbons with HC in red and LC in blue. The C1 nanobody in (B) is coloured in orange. Related to Figures 4,5 & 6.

Figure S1

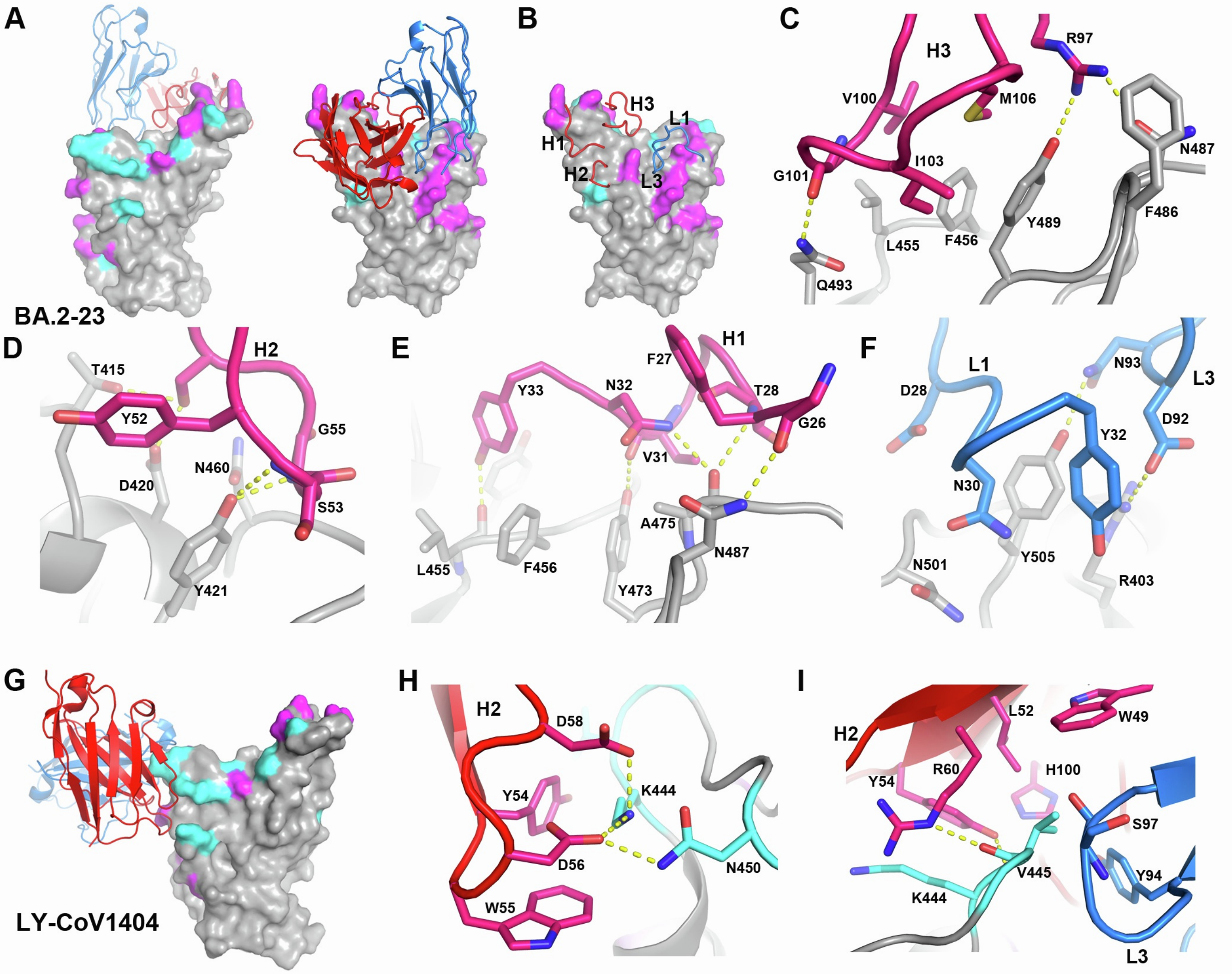

Figure S2. Structure of Delta-RBD and BA.2-23 complex and sensitivity of LY-CoV1404 to Omicron subvariants containing K444T or V450P mutation indicated by the structure of its complex with RBD. (A) overall structure of RBD and BA.2-23 complex. (B)-(F) details of interactions between RBD and BA.2-23. Drawing style and colour scheme are as in Figure 6. (G) Overall structure of RBD/LY-CoV1404 (PDB ID, 7MMO) as viewed from front of the RBD. The drawing style and colour scheme are as in (A). (H) Interactions of K444 of the RBD with CDR-H2 of LY-CoV1404. (I) Contacts between V445 of the RBD and LY-CoV1404. The side chains of the RBD, Fab HC and LC are shown as cyan, red and blue sticks respectively. Hydrogen bonds and salt bridges are shown as yellow broken bonds. Related to Figure 6.

Figure S2

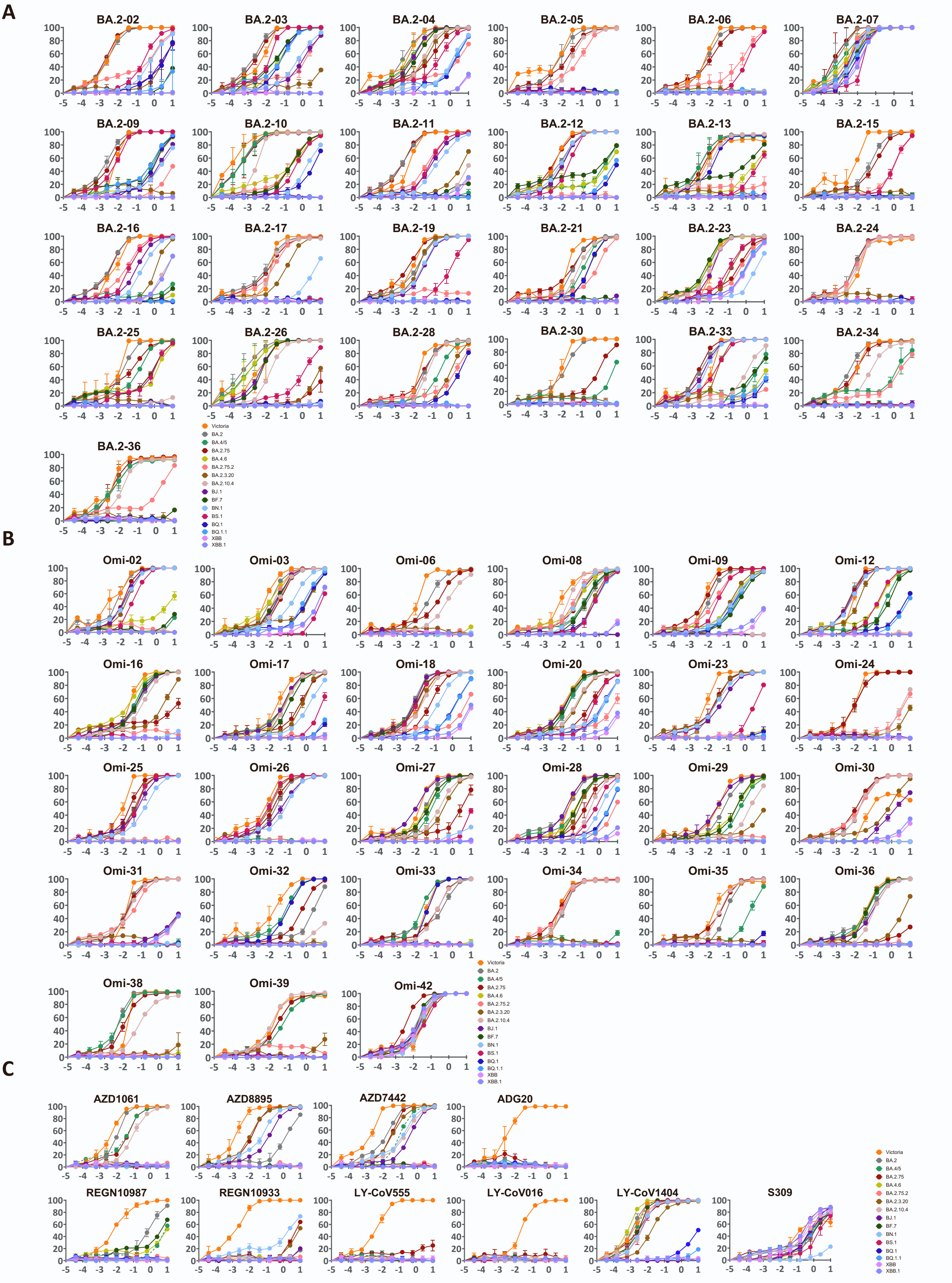

Figure S3. Neutralization assays. Neutralization curves using lentivirus pseudotyped with the S gene of the indicated BA.2 sub-lineages (A) BA.2 mAbs, (B) Omi-mAbs, (C) Commercial mAb. Related to Figure 3. Data for Omi-mAbs and Commercial mAbs against Victoria, BA.2, BA.2.75, BA.4/5, and BA.4.6 previously reported are included for comparison[S4,S5,S6].

Figure S3

Compiled IC50 titres of BA.1 mAb, data for various viruses

mAbs	WT virus								Psudo-virus																	
	Victoria[13]	Alpha[13]	Beta[13]	Gamma[13]	Delta[13]	BA.1[13]	BA.1.1[13]	BA.2[13]	Victoria[24]	BA.2[24]	BA.2.12.1[23]	BA.4/5[24]	BA.4.6[41]	BA.2.75[42]	BA.2.75.2	BA.2.3.20	BJ.1	BF.7	BQ.1	BQ.1.1	BS.1	BA.2.10.4	BN.1	XBB	XBB.1
Omi-29 (3-53)	0.044	0.066	0.048	0.040	0.029	0.036	0.052	0.192	0.014	0.056	0.011	0.396	0.170	10.000	10.000	10.000	0.025	0.266	0.266	10.000	10.000	1.738	10.000	10.000	10.000
Omi-36 (3-66)	0.056	0.047	0.018	0.015	0.026	0.038	0.053	0.105	0.022	0.030	0.051	0.024	0.029	10.000	10.000	10.000	0.045	0.043	10.000	10.000	10.000	0.110	10.000	10.000	10.000
Omi-16 (3-66)	0.016	0.022	0.018	0.022	0.016	0.019	0.027	0.067	0.014	0.034	0.017	0.029	0.007	10.000	10.000	1.075	0.025	0.034	0.034	10.000	10.000	0.073	10.000	10.000	10.000
Omi-27 (3-66)	0.026	0.032	0.019	0.017	0.010	0.091	0.239	0.039	0.008	0.034	0.005	0.069	0.023	6.672	10.000	0.215	0.007	0.032	0.032	10.000	10.000	0.140	10.000	10.000	10.000
Omi-17 (3-66)	0.066	0.098	0.021	0.021	0.074	0.028	0.026	0.095	0.023	0.060	0.022	0.028	0.039	0.255	10.000	0.347	0.030	0.100	0.100	10.000	6.675	0.068	1.294	10.000	10.000
Omi-20 (3-66)	0.012	0.023	0.019	0.019	0.008	0.043	0.032	0.022	0.009	0.015	0.007	0.014	0.008	0.178	10.000	0.030	0.009	0.014	1.222	1.479	0.481	0.041	1.017	10.000	10.000
Omi-28 (3-66)	0.028	0.028	0.019	0.033	0.018	0.032	0.075	0.047	0.022	0.008	0.007	0.028	0.035	0.133	10.000	0.053	0.010	0.056	3.382	10.000	0.555	0.323	1.191	10.000	10.000
Omi-18 (3-53)	0.041	0.038	0.018	0.016	0.025	0.006	0.006	0.007	0.008	0.005	0.002	0.005	0.006	0.035	10.000	0.011	0.005	0.007	1.021	1.143	0.009	0.017	0.134	10.000	10.000
Omi-03 (3-53)	0.007	0.012	0.009	0.004	0.004	0.009	0.015	0.028	0.003	0.008	0.005	0.017	0.006	0.017	0.546	0.020	0.014	0.016	0.681	0.541	6.895	0.024	0.088	4.239	4.876
Omi-24 (1-69)	0.005	0.008	0.006	0.010	10.000	0.007	0.035	0.008	0.005	0.007	10.000	10.000	10.000	0.008	10.000	10.000	10.000	10.000	10.000	10.000	10.000	3.889	10.000	10.000	10.000
Omi-31 (1-69)	0.007	0.020	0.011	0.017	10.000	0.010	0.017	0.083	0.376	0.013	10.000	10.000	10.000	0.014	0.012	10.000	10.000	10.000	10.000	10.000	10.000	0.014	10.000	10.000	10.000
Omi-34 (1-69)	0.003	0.041	0.003	0.008	10.000	0.028	0.074	0.014	0.007	0.009	10.000	10.000	10.000	0.005	0.005	10.000	10.000	10.000	10.000	10.000	10.000	0.011	10.000	10.000	10.000
Omi-30 (1-69)	0.109	0.043	0.028	0.038	10.000	0.058	0.084	0.045	0.012	0.011	10.000	10.000	10.000	0.008	0.009	0.343	1.827	10.000	10.000	10.000	10.000	0.017	10.000	10.000	10.000
Omi-02 (1-69)	0.015	0.014	0.009	0.004	0.014	0.013	0.015	0.040	0.002	0.003	0.004	10.000	10.000	0.009	10.000	0.013	0.011	10.000	10.000	10.000	0.052	10.000	0.013	10.000	10.000
Omi-38 (1-69)	0.001	0.009	0.004	0.002	0.004	0.054	10.000	0.027	0.015	0.005	0.004	0.005	10.000	0.011	10.000	10.000	10.000	10.000	10.000	10.000	10.000	0.101	10.000	10.000	10.000
Omi-35 (3-9)	0.057	0.080	0.128	0.136	0.280	0.069	0.262	0.082	0.018	0.094	0.667	1.687	10.000	0.020	10.000	10.000	10.000	10.000	10.000	10.000	10.000	0.039	10.000	10.000	10.000
Omi-25 (3-9)	0.003	0.007	0.059	0.257	0.006	0.046	0.138	0.056	0.005	0.024	0.007	10.000	10.000	0.014	10.000	0.025	0.041	10.000	10.000	10.000	0.040	10.000	0.097	10.000	10.000
Omi-42 (3-9)	0.016	0.024	0.011	0.013	0.019	0.014	0.017	0.031	0.013	0.021	0.009	0.013	0.010	0.003	0.011	0.018	0.010	0.011	0.013	0.021	0.023	0.023	0.024	0.017	0.009
Omi-26 (1-18)	0.005	0.010	0.055	0.214	0.005	0.034	0.055	0.030	0.002	0.013	0.007	10.000	10.000	0.010	10.000	0.006	0.031	10.000	10.000	10.000	0.005	10.000	0.041	10.000	10.000
Omi-32 (3-33)	0.032	0.102	0.460	0.430	0.012	0.024	4.642	1.899	0.010	2.682	0.070	0.035	10.000	0.354	10.000	10.000	10.000	10.000	0.061	10.000	10.000	10.000	10.000	10.000	10.000
Omi-33 (3-33)	0.028	0.057	0.136	0.132	0.011	0.026	0.113	0.681	0.027	0.068	0.008	0.013	10.000	0.053	10.000	10.000	10.000	10.000	0.041	10.000	10.000	0.109	10.000	10.000	10.000
Omi-06 (4-4)	0.007	0.011	0.012	0.010	5.040	0.054	1.505	0.238	0.007	0.039	10.000	10.000	10.000	0.063	10.000	10.000	10.000	10.000	10.000	10.000	10.000	0.420	10.000	10.000	10.000
Omi-23 (4-31)	0.005	0.009	0.020	0.018	0.006	0.044	0.030	0.028	0.005	0.019	0.009	10.000	10.000	0.011	10.000	0.009	0.024	10.000	10.000	10.000	3.113	10.000	0.018	10.000	10.000
Omi-39 (3-43)	0.015	0.039	0.009	0.014	0.012	0.025	10.000	0.073	0.014	0.026	0.018	0.035	10.000	0.027	10.000	10.000	10.000	10.000	10.000	10.000	10.000	0.011	10.000	10.000	10.000
Omi-09 (3-30)	0.004	0.002	1.218	2.373	0.008	0.011	0.017	0.034	0.006	0.008	0.016	0.166	0.108	0.003	0.012	0.133	10.000	0.376	0.215	0.217	0.029	10.000	0.165	10.000	10.000
Omi-08 (1-46)	0.014	0.022	0.007	0.024	0.048	0.008	0.007	1.510	0.008	0.114	0.099	0.086	0.033	0.036	0.027	0.426	10.000	0.192	0.501	0.633	0.584	0.024	0.063	10.000	10.000
Omi-12 (1-58)	0.005	0.003	0.006	0.003	0.003	0.004	0.009	0.010	0.006	0.003	0.002	0.429	0.074	0.003	10.000	0.008	0.004	0.496	5.902	7.949	0.167	10.000	0.004	10.000	10.000

μg/mL
10.000
0.100
0.001

Figure S4 Heat map of IC50 neutralization titres for the panel of BA.1 (Omi) mAb. Pseudoviral neutralization IC50 titres for indicated mAb against a panel of pseudoviruses expressing variant S sequences. Live virus IC50 values against variants found earlier in the pandemic are included for comparison. Data for live virus assays[S3] and pseudoviral data for Victoria, BA.2, BA.2.12.1, BA.2.75, BA.4/5, and BA.4.6 previously reported are included for comparison[S3,S4,S5,S6,S7]. Related to Figure 3.

Figure S4

Table S1. Sources of BA.2 sub-lineage sequences, related to Figure 1.

Lineage	Defining RBD mutations	Example early genome	Submitting scientist, laboratory	Country/Region of earliest sequences	Date of earliest sequences	Pango issue, contributor
BA.4.6	BA.4/5 + R346T	EPI_ISL_12475182	Oliver et al, HOSPITAL UNIVERSITARIO SON ESPASES	Europe/South Africa	April 2022	#741, ryhisner
BA.4.7	BA.4/5 + R346S	EPI_ISL_12644817	Iranzadeh et al. NHLS/UCT	South Africa/Israel	April 2022	#777, FedeGueli
BF.7(BA.5.2.1.7)	BA.4/5 R346T	EPI_ISL_12810243	Coppens et al., Labo Klinische Biologie, UZA	Belgium	May 2022	#827, ryhisner
BQ.1(BA.5.3.1.1.1.1.1)	BA.4/5 K444T, N460K	EPI_ISL_14294806	Howard et al., Centers for Disease Control and Prevention Division of Viral Diseases, Pathogen Discovery	Nigeria	July 2022	#993, FedeGueli
BQ.1.1(BA.5.3.1.1.1.1.1.1)	BQ.1 + R346T	EPI_ISL_14752457	Christensen et al., Houston Methodist Hospital	USA	August 2022	#993, FedeGueli
BA.2.75	BA.2 + G339H, G446S, N460K, R493Q*	EPI_ISL_13302209	Khairnar et al. CSIR-NEERI, Nagpur Covid-19 Testing Lab	India	April 2022	#773, Silcn
BA.2.75.2	BA.2.75 + R346T, F486S	EPI_ISL_14290506	Gupta et al. ILBS/INSACOG	India	July 2022	#966, agamedilab
BN.1(BA.2.75.5.1)	BA.2.75 + R346T, K356T, F490S	EPI_ISL_14601644	Sima et al, Lifebrain Covid Labor GmbH	India	July 2022	#994, corneliusroemer
BJ.1(BA.2.10.1.1)	BA.2 + G339H, R346T, L368I, V445P, G446S, V483A, F490V	EPI_ISL_14166909	Maitra et al. National Institute of Biomedical Genomics – INSACOG	India	June 2022	#915, Silcn
BA.2.10.4	BA.2 + G446S, F486P, R493Q*, S494P	EPI_ISL_13929780	Karyakarte et al. Center for Genomics, Department of Microbiology, BJ Government Medical College and Sassoon Hospitals	India	June 2022	#898, Silcn
BS.1(BA.2.3.2.1)	BA.2 + R346T, L452R, N460K, G476S	EPI_ISL_14853710	Sekizuka et al. Pathogen Genomics Center, National Institute of Infectious Diseases	Japan ex Vietnam	August 2022	#1052, TakaKen6
BA.2.3.20	K444R, N450D, L452M, N460K, E484R, R493Q*	EPI_ISL_14723265	Selway et al, SA Pathology	USA/Singapore/Australia	August 2022	#1013, ryhisner
XBB	BA.2 + R346T, L368I, V445P, G446S, N460K, F486S, F490S, R493Q*	EPI_ISL_14917761	Ngan et al, National Public Health Laboratory, National Centre for Infectious Diseases	India	August 2022	#1058, corneliusroemer

Table S2. Variable gene usage BA.2 antibodies, related to Figure 1.

Ab id.	Protein-Specific	Heavy Chain					Light Chain				
		V-GENE and allele	J-GENE and allele	D-GENE and allele	V-REGION Nb of AA changes	CDR3 length	Light Chain	V-GENE and allele	J-GENE and allele	V-REGION Nb of AA changes	CDR3 length
BA.2-02	RBD	1-69*01 F, or 1-69D*01 F	5*02 F	3-22*01 F	10	19	K	1-39*01 F, or 1D-39*01 F	4*01 F	7	9
BA.2-03	RBD	1-2*02 F	6*02 F	3-16*02 F	10	23	K	3-11*01 F	5*01 F	6	9
BA.2-04	RBD	3-53*02 F	6*02 F	3-9*01 F	11	11	K	1-9*01 F	5*01 F	8	10
BA.2-05	RBD	1-69*06 F, or 1-69*17 F	6*02 F	3-3*02 F	9	17	λ	1-47*01 F	3*02 F	10	11
BA.2-06	RBD	1-69*01 F, or 1-69D*01 F	4*02 F	3-22*01 F	8	20	K	1-39*01 F, or 1D-39*01 F	1*01 F	4	9
BA.2-07	RBD	1-69*01 F, or 1-69D*01 F	5*02 F	3-22*01 F	13	19	K	1-39*01 F, or 1D-39*01 F	4*01 F	8	9
BA.2-09	RBD	1-2*02 F	6*02 F	3-16*02 F	11	23	K	3-11*01 F	5*01 F	8	9
BA.2-10	RBD	3-9*01 F	3*02 F	3-22*01 F	7	18	K	1-39*01 F, or 1D-39*01 F	3*01 F	8	9
BA.2-11	RBD	1-69*01 F, or 1-69D*01 F	5*02 F	3-22*01 F	9	19	K	1-39*01 F, or 1D-39*01 F	4*01 F	5	9
BA.2-12	RBD	3-9*01 F	6*02 F	2-21*02 F	8	16	K	1-39*01 F, or 1D-39*01 F	2*01 F	13	9
BA.2-13	RBD	3-15*01 F	3*01 F	3-10*01 F	9	17	K	1-39*01 F, or 1D-39*01 F	4*01 F	4	8
BA.2-15	RBD	1-69*01 F, or 1-69D*01 F	4*02 F	3-22*01 F	10	20	K	1-39*01 F, or 1D-39*01 F	1*01 F	3	9
BA.2-16	RBD	1-69*01 F, or 1-69D*01 F	5*02 F	3-22*01 F	9	19	K	1-39*01 F, or 1D-39*01 F	4*01 F	7	9
BA.2-17	RBD	1-69*06 F	6*02 F	3-3*01 F	9	19	λ	2-14*01 F	2*01 F, or 3*01 F	8	11
BA.2-19	RBD	3-48*03 F	4*02 F	1-26*01 F	8	13	K	1-5*03 F	1*01 F	6	10
BA.2-21	RBD	3-9*01 F	4*02 F	5-24*01 ORF	11	16	λ	1-40*01 F	3*02 F	7	11
BA.2-23	RBD	3-53*04 F	6*02 F	1-26*01 F	12	13	K	1-33*01 F, or 1D-33*01 F	5*01 F	8	9
BA.2-24	RBD	1-69*09 F	4*02 F	3-22*01 F	13	16	K	1-39*01 F, or 1D-39*01 F	5*01 F	6	9
BA.2-25	RBD	4-59*01 F	4*02 F	5-12*01 F	16	16	K	2-28*01 F, or 2D-28*01 F	3*01 F	7	9
BA.2-26	RBD	3-66*01 F, or 3-66*04 F	5*01 F, or 5*02 F	2-15*01 F	16	10	K	1-33*01 F, or 1D-33*01 F	4*02 (F)	10	8
BA.2-28	RBD	3-9*01 F	6*02 F	6-6*01 F	14	19	λ	3-21*02 F	2*01 F, or 3*01 F	6	11
BA.2-30	RBD	4-59*03 F	4*02 F	5-12*01 F	13	16	K	2-28*01 F, or 2D-28*01 F	3*01 F	6	9
BA.2-33	RBD	4-61*11 (F)	5*02 F	2-15*01 F	16	14	λ	1-47*01 F	2*01 F, or 3*01 F	13	11
BA.2-34	RBD	1-69*09 F	4*02 F	2-21*02 F	11	12	K	1-5*01 F	1*01 F	4	8
BA.2-36	RBD	4-61*02 F, or 4-61*11 (F)	4*02 F	6-25*01 F	17	11	K	1-5*01 F	1*01 F, or 4*02 (F)	7	8

Table S2

Table S3. Structural data collection, analysis and model statistics, related to Figure 6.

**Method**	X-ray Crystallography			Cryo-EM	
**Structure**	Delta-RBD/BA.2-10/EY6A	Delta-RBD/BA.2-13/C1	Delta-RBD/BA.2-36		Delta-RBD/BA.2-23/BA.2-36/EY6A/Fab-45
**PDB/EMBD ID**	8BBN	8C3V	8BBO		8BCZ, EMD-15971
**Data collection**					
Space group	$P2_12_12_1$	$P2_12_12_1$	$P4_1$	Voltage (kV)	300
Cell dimensions				Frames (EER fractions)	50
a, *b*, *c* (Å)	170.7, 171.7, 177.0	105.9, 160.8, 172.4	99.2, 99.2, 86.1	Dose rate ($e^-/Å^2/s$)	11.5
α, β, γ (°)	90, 90, 90	90, 90, 90	90, 90, 90	Total dose ($e^-/Å^2$)	50
Resolution (Å)	56–3.58 (3.65–3.58)[a]	80–2.74 (2.79–2.74)	54–2.75 (2.80–2.75)	Calibrated pixel size ($Å^2$)	0.7303
R_{merge}	0.467 (---)	0.257	0.460 (---)	Defocus (μm)	0.8-2.6
R_{pim}	0.130 (1.771)	0.072 (1.078)	0.90 (2.066		
I/*s*(*I*)	4.1 (0.3)	7.4 (0.4)	7.5 (0.4)	Movies	20,535
$CC_{1/2}$	0.992 (0.301)	0.995 (0.317)	0.994 (0.390)	Particles (final)	167,492
Completeness (%)	100 (97.2)	100 (99.5)	100 (99.9)	Map resolution (Å)	2.9
Redundancy	13.8 (13.2)	13.7 (14.1)	26.8 (18.3)	Sharpening B-factor ($Å^2$)	90.5
**Refinement**					
Resolution (Å)	56–3.58	67–2.74	50–2.75	Resolution (Å)	2.9
No. reflections	52453/2840	73940/3933	20668/1102	No. protein atoms	8505
R_{work} / R_{free}	0.265/0.313	0.216/0.259	0.224/0.252	*B* factors ($Å^2$)	62
No. atoms				r.m.s. deviations	
Protein	24354	17512	4738	Bond lengths (Å)	0.003
Ligand/ion/water		285	14	Bond angles (°)	0.5
B factors ($Å^2$)				Clash score	6.2
Protein	174	93	81	Ramachandran outlier (%)	0
Ligand/ion/water		101	126	Rotamer outlier (%)	1.2
r.m.s. deviations				d FSC model (0.5)	3.1
Bond lengths (Å)	0.002	0.003	0.002	CC (mask)	0.83
Bond angles (°)	0.5	0.6	0.5		

Table S4. Sample information, related to Star Methods.

	BA.1 infection	BA.2 infection	BA.4/5 infection	BNT162b2 V3+28	
Participants					
Female	6	19	6	8	
Male	3	4	5	10	
Median Age (Y)	31 (Range 21-55)	41 (Range 22-57)	42 (Range 20-94)	45 (Range 30-59)	

Supplemental References

S1. Dejnirattisai, W., Zhou, D., Ginn, H.M., Duyvesteyn, H.M.E., Supasa, P., Case, J.B., Zhao, Y., Walter, T.S., Mentzer, A.J., Liu, C., et al. (2021). The antigenic anatomy of SARS-CoV-2 receptor binding domain. Cell *184*, 2183-2200.

S2. Liu, C., Zhou, D., Nutalai, R., Duyvestyn, H., Tuekprakhon, A., Ginn, H., Dejnirattisai, W., Supasa, P., Mentzer, A., Wang, B., et al. (2021). The Beta mAb response underscores the antigenic distance to other SARS-CoV-2 variants. Cell, Host and Microbe *30*, 53-68.

S3. Nutalai, R., Zhou, D., Tuekprakhon, A., Ginn, H., Supasa, P., Liu, C., Huo, J., Mentzer, A., Duyvesteyn, H.M.E., Dijokaite-Guraliuc, A., et al. (2022). Potent cross-reactive antibodies following Omicron breakthrough in vaccinees. Cell *185*, 2116-2131.

S4. Tuekprakhon, A., Nutalai, R., Dijokaite-Guraliuc, A., Zhou, D., Ginn, H.M., Selvaraj, M., Liu, C., Mentzer, A.J., Supasa, P., Duyvesteyn, H.M.E., et al. (2022). Antibody escape of SARS-CoV-2 Omicron BA.4 and BA.5 from vaccine and BA.1 serum. Cell 185, 2422-2433 e2413. 10.1016/j.cell.2022.06.005.

S5. Dijokaite-Guraliuc, A., Das, R., Nutalai, R., Zhou, D., Mentzer, A.J., Liu, C., Supasa, P., Dunachie, S.J., Lambe, T., Fry, E.E., et al. (2022). Antigenic characterization of SARSCoV-2 Omicron subvariant BA.4.6. Cell Discov 8, 127. 10.1038/s41421-022-00493-0.

S6. Huo, J., Dijokaite-Guraliuc, A., Liu, C., Zhou, D., Ginn, H.M., Das, R., Supasa, P., Selvaraj, M., Nutalai, R., Tuekprakhon, A., et al. (2023). A delicate balance between antibody evasion and ACE2 affinity for Omicron BA.2.75. Cell Rep 42, 111903. 10.1016/j.celrep.2022.111903.

S7. Huo, J., Dijokaite-Guraliuc, A., Nutalai, R., Das, R., Zhou, D., Mentzer, A., Fry, E., Mongkolsapaya, J., Ren, J., Stuart, D., and Screaton, G. (2022). Humoral responses against SARS-CoV-2 Omicron BA.2.11, BA.2.12.1 and BA.2.13 from vaccine and BA.1 serum. Cell Discovery.
